# Putative PINK1/Parkin activators lower the threshold for mitophagy by sensitizing cells to mitochondrial stress

**DOI:** 10.1126/sciadv.ady0240

**Published:** 2025-08-27

**Authors:** William M. Rosencrans, Ryan W. Lee, Logan McGraw, Ian Horsburgh, Ting-Yu Wang, Baiyi Quan, Diana Huynh, Jennifer A. Johnston, David C. Chan, Tsui-Fen Chou

**Affiliations:** ^1^ Division of Biology and Biological Engineering, California Institute of Technology, Pasadena, CA, USA.; ^2^ National Institute of Neurological Disorders and Stroke, NIH, Bethesda, MD, USA.; ^3^ NysnoBio, Mill Valley, CA, USA.

## Abstract

The PINK1/Parkin pathway targets damaged mitochondria for degradation via mitophagy. Genetic evidence implicates impaired mitophagy in Parkinson’s disease, making its pharmacological enhancement a promising therapeutic strategy. Here, we characterize two mitophagy activators: a novel Parkin activator, FB231, and the reported PINK1 activator MTK458. Both compounds lower the threshold for mitochondrial toxins to induce PINK1/Parkin-mediated mitophagy. However, global proteomics revealed that FB231 and MTK458 independently induce mild mitochondrial stress, resulting in impaired mitochondrial function and activation of the integrated stress response, effects that result from PINK1/Parkin-independent off-target activities. We find that these compounds impair mitochondria by distinct mechanisms and synergistically decrease mitochondrial function and cell viability in combination with classical mitochondrial toxins. Our findings support a model whereby weak or “silent” mitochondrial toxins potentiate other mitochondrial stressors, enhancing PINK1/Parkin-mediated mitophagy. These insights highlight important considerations for therapeutic strategies targeting mitophagy activation in Parkinson’s disease.

## Teaser

Drugs targeting mitochondrial removal show unexpected mechanisms with implications for Parkinson’s therapeutics.

## INTRODUCTION

Parkinson’s disease (PD) is the second most common neurodegenerative disease. Affecting more than 10 million people worldwide, there exists no treatment that corrects the underlying mechanism (*1*). The disease is predominantly characterized by loss of neurons in the dopamine-producing substantia nigra (*2*). PD is characterized at the molecular level by accumulation of protein aggregates known as Lewy bodies as well as mitochondrial dysfunction (*2*). Multiple lines of evidence implicate mitochondrial defects in the initial pathogenesis of the disease (*3*). Mitochondrial toxins such as rotenone, paraquat, and 1-methyl-4-phenyl-1,2,3,6-tetrahydropyridine (MPTP) are associated with development of PD (*4*). Moreover, postmortem samples from patients with idiopathic PD demonstrate defects in mitochondrial function (*5*).

The strongest tie between mitochondrial dysfunction and PD is genetic evidence demonstrating that the loss of mitochondrial quality control pathways cause familial forms of PD. Mutations in the Parkin and PINK1 proteins are the most common form of autosomal recessive PD (*6*). Parkin is an E3 ligase that functions together with the ubiquitin kinase PINK1 to flag damaged mitochondria for removal via a selective form of autophagy known as mitophagy. Under healthy conditions, PINK1 is continually degraded and kept at very low levels. PINK1 is translated in the cytosol and imported into the mitochondria via the translocase of the outer mitochondrial membrane (TOM) and the translocase of the inner membrane (TIM). It is then cleaved by the proteases MPP and PARL and released back into the cytosol (*7*). Cleavage reveals a potent n-degron, resulting in rapid proteasomal degradation of PINK1 (*8*). In conditions of mitochondrial stress, including mitochondrial membrane potential (MMP) loss or protein aggregation, PINK1 fails to import and instead remains stabilized on the TOM complex leading to its accumulation, dimerization, and activation (*9*–*13*). Active PINK1 phosphorylates free monomeric as well as outer mitochondrial membrane (OMM) protein conjugated ubiquitin on serine-65 (pUb) (*14*, *15*). Accumulation of the unique pUb signal recruits cytosolic Parkin to the mitochondria where it is also phosphorylated by PINK1 (*14*, *16*, *17*). Binding of the pUb partially activates Parkin from its autoinhibited state by allosterically releasing the inhibiting ubiquitin-like domain (Ubl) from RING1 (*18*–*22*). PINK1 can then phosphorylate Parkin at the Ubl, freeing Parkin’s catalytic cysteine 431 to bind ubiquitin on E2-ubiquitin conjugating enzymes (*23*).

Parkin generates ubiquitin chains including, notably K63-linked Ub, on the OMM, which in turn can be phosphorylated by PINK1, leading to further Parkin recruitment in a feed-forward mechanism (*9*). This process can be opposed by deubiquitinases such as USP30, which remove ubiquitin from the OMM (*24*). Accumulation of the K63-Ub chains recruits a series of autophagy adaptor proteins including NDP52 and OPTN that initiate the formation of the autophagosome at the damaged mitochondria via recruitment of the autophagy initiation complex (*25*–*27*). This feed-forward mechanism leads to a strong switch-like behavior that can rapidly clear damaged mitochondria upon PINK1 accumulation (*28*, *29*).

Beyond the subset of patients with familial PD with PINK1/Parkin mutations, there is evidence suggesting that the pathway may be impaired in the idiopathic PD population. Increased levels of pUb are found in the brains and plasma of patients with PD, suggesting an impairment in mitophagy completion downstream of PINK1 (*30*, *31*). Moreover, mitochondrial toxins have been used as potent pharmacological models of PD for the last 30 years, since the discovery that mitochondrial toxin MPTP could directly cause Parkinsonism in humans (*32*). Enhancement of PINK1/Parkin mitophagy represents a strategy toward improving mitochondrial quality control and enabling a disease-modifying strategy in PD (*33*).

Multiple small-molecule compounds have been developed with the reported ability to enhance PINK1/Parkin mitophagy (*34*). These include USP30 inhibitors that have shown promise in enhancing mitophagy and protecting cells in PD models (*35*). A PINK1-activating compound, MTK458, was recently reported by the companies Mitokinin and Abbvie (*30*). A potential derivative of MTK458, ABBV1088, is in phase 1 clinical trials toward eventual treatment of PD (*34*). MTK458 was shown to enhance mitophagy in contexts of mitochondrial stress, but no direct measurement of PINK1 binding has been shown. PINK1-inhibiting compounds have also been described (*36*). Parkin-targeting compounds have remained hitherto unsuccessful in inducing mitophagy in wild-type (WT) cell models. Biogen reported a direct Parkin-activating compound that showed potent in vitro Parkin activation but failed to enhance mitophagy in vivo (*37*). Recent work on the Biogen compounds suggests that they act as “molecular glues” between Parkin and pUb and can restore activity to two rare point mutants of Parkin in cells (*38*). These results demonstrate the potential for small molecule–based Parkin activators but highlight that potent in vitro activation does not necessarily translate to in vivo augmentation of the PINK1/Parkin pathway.

In this work, we describe the compound FB231 that enhances Parkin activity in vitro. Developing a high-throughput assay for mitophagy induction, we quantitatively compare FB231 and the recently reported PINK1 activator MTK458 and show that both compounds reduce the threshold for mitophagy induction via the PINK1/Parkin pathway across cell types. We demonstrate that these activators can be used in combination to synergistically enhance mitophagy. Using label-free proteomics and biochemistry, we show that FB231 causes activation of the integrated stress response (ISR) and perturbation to iron-dependent pathways. We unexpectedly find that these compounds act as weak mitochondrial toxins that sensitize mitochondria to damage by classical mitochondrial stressors and lead to activation of the mitochondrial ISR (mitoISR) and PINK1 accumulation. By limiting mitochondrial function, these compounds ultimately impair cell growth and sensitize cells to death from mitochondrial toxins. These data suggest a hitherto unknown common pitfall for current pharmacological screens, whereby weak mitochondrial toxins can act as potent PINK1/Parkin activators.

## RESULTS

### Identification of a putative Parkin-activating compound through in vitro activity assays

We had previously identified small molecules capable of activating Parkin (*39*). The compound used in this study, FB231, was subsequently identified in a high-throughput assay for Parkin activators based on Parkin ubiquitylation activity. In our scheme, we used a His_6_-tagged full-length Parkin construct (FL-Parkin) and a derivatized ubiquitin suicide probe [vinyl-sulfone modified ubiquitin (Ub-VS)] as described in Riley *et al.* (*23*). Because the Ub-VS probe selectively reacts with the activated conformation to form a covalent bond, we used this assay to screen small molecules capable of activating Parkin. Binding of the probe to Parkin was measured by a Förster resonance energy transfer (FRET) readout. We used the sensitive Europium-Cryptate (Eu) + d2 FRET pair, by using Europium tagged anti–hemagglutinin (HA) antibody that binds the Ub-VS probe and a d2-tagged anti-His_6_ antibody that binds to the tagged Parkin (fig. S1A) (*38*, *40*).

A potent compound identified in this screen, CMPD001, had already been flagged as a potential activator of Parkin in a previous screen (compound X) and patented (*39*, *41*). CMPD001 has an EC_30_ of 3.9 μM in the UB-VS assay (fig. S1C) and was therefore used as an internal control for assessing the activity of subsequent compounds. We also used a second assay, TR-FRET, that measured Parkin autoubiquitination by the covalent attachment of Europium tagged Ub. CMPD001 showed similar potency in this autoubiquitination assay (fig. S1D).

**Fig. 1.**
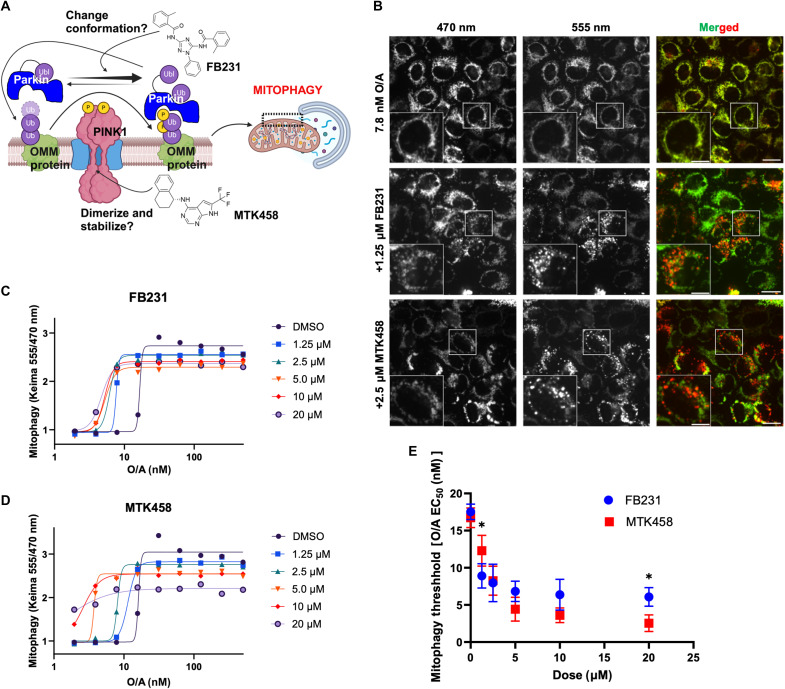
PINK1/Parkin activators lower the threshold for mitophagy induction. (**A**) Diagram detailing the proposed mechanism of FB231 and MTK458 in activation of the PINK1/Parkin pathway. (**B**) Confocal imaging of mt-Keima–expressing HeLa cells after treatment with 7.8 nM O/A alone, 7.8 nM O/A and 1.25 μM FB231, or 7.8 nM O/A and 2.5 μM MTK458 for 6 hours. Scale bars (overviews), 20 μm; (insets), 10 μm. (**C**) mt-Keima assay of YFP-Parkin mt-Keima HeLa cells treated with varying doses of O/A and different doses of the Parkin activator FB231 for 6 hours, each colored uniquely. Data are normalized to dimethyl sulfoxide (DMSO) alone at 6 hours. Solid lines are fits to the Hill equation to determine the EC_50_. (**D**) As in (C), with the compound MTK458. (**E**) Mitophagy induction threshold, or the O/A EC_50_, is calculated for each dose of FB231 (blue) and MTK458 (red). Symbols are means ± SD from three independent experiments; *P ≤ 0.05, two-way analysis of variance (ANOVA).

We pursued derivatives of CMPD001 that could improve its pharmacokinetic properties. FB231 is a derivative of CMPD001 with two methyl substitutions at the ortho position of both benzamides (Fig. 1B). It showed improved drug metabolism and pharmacokinetic properties across a range of in vitro and in vivo assays, especially a longer half-life (fig. S1B and table S4). FB231 had slightly less potency than CMPD001 in the UB-VS assay and no activity in the autoubiquitination assay (fig. S1D). Despite unclear activity in vitro, preliminary work in cell models suggested that FB231 could accentuate PINK1/Parkin mitophagy in cells exposed to low levels of mitochondrial toxins (*41*). These results were similar to data reported for the clinically relevant reported PINK1 activator, MTK458 (*30*). Given the improved pharmacology profile, preliminary cellular activity, and potential for clinical relevance, we next sought to rigorously determine whether these compounds had the ability to alter mitophagy in a cellular context.

### Putative Parkin and PINK1 activators reduce the threshold for mitophagy activation by antimycin/oligomycin

In the context of mitochondrial stress, the PINK1/Parkin pathway becomes activated by stabilization and retention of PINK1 at the OMM through its interaction with the TOM complex (*9*). This complex can be stabilized by membrane depolarization and mitochondrial protein aggregation (*9*, *12*). In cell culture models, membrane depolarization is readily accomplished through chemical manipulation. Ionophores including carbonyl cyanide *m*-chlorophenyl hydrazone (CCCP) and carbonyl cyanide *p*-trifluoromethoxyphenylhydrazone (FCCP) permit protons to directly pass through the inner mitochondrial membrane. Alternatively, toxins targeting complexes of the electron transport chain are also commonly used to activate PINK1. These include complex III inhibitor antimycin A and complex V inhibitor oligomycin A. Although having no activity on its own, FB231 was shown to accelerate mitophagy in cells exposed to CCCP (*41*). The reported PINK1 activator MTK458 was screened in the presence of FCCP and oligomycin (*30*). MTK458 is hypothesized to stabilize the active form of PINK1, leading to enhanced mitophagy in the presence of stress (Fig. 1A). We therefore compared FB231 to MTK458 as two compounds that sensitize cells to mitophagy in the presence of additional mitochondrial stress.

We first sought to establish a consistent regimen for testing mitophagy-activating compounds. We chose to use a cocktail of equimolar oligomycin/antimycin (O/A) widely used in mitophagy studies rather than ionophores CCCP/FCCP, which have been reported to have more pleiotropic effects (*25*, *42*). O/A is usually used at micromolar-level doses to maximally induce PINK1/Parkin mitophagy. Because WT HeLa cells are devoid of Parkin, we used HeLa cells expressing yellow fluorescent protein (YFP)–Parkin and the mitophagy reporter mt-Keima. Mt-Keima is a mitochondrial-targeted, pH-sensitive fluorescent protein used to monitor mitophagy by distinguishing mitochondria in acidic autolysosomes from those in the neutral cytoplasm. It shifts its fluorescence excitation when exposed to different pH levels, enabling ratiometric quantification of mitophagy (*43*).

We used high-content confocal microscopy to determine the extent of mitophagy with varying combinatorial doses of O/A with the experimental compounds MTK458 and FB231. Pan-caspase inhibitor quinolyl-valyl-*O*-methylaspartyl-[-2,6-difluorophenoxy]-methyl ketone (QVD) was also added to cells to prevent apoptosis. Mitolysosomes could be observed as puncta expressing high 555-nm fluorescence with low 470-nm fluorescence (Fig. 1B and fig. S2) (*25*).

In the absence of PINK1/Parkin activators, 6 hours of O/A treatment showed a threshold of 15 nM for inducing mitophagy and quickly approached saturation by 31.25 nM (Fig. 1, C and D). We monitored mitophagy with varying concentrations of O/A in the presence of either FB231 or MTK458. We found that both FB231 and MTK458 lowered the O/A threshold for induction of mitophagy (Fig. 1, C to E). However, in the absence of O/A, both compounds fail to induce mitophagy at any dose tested, up to 20 μM. For varying doses of the activators, we calculated the median effective concentration (EC_50_) of O/A, referred to hereafter as the mitophagy induction threshold (Fig. 1E). FB231 reduced the mitophagy induction threshold of O/A from ~16.6 to 7.6 nM at 1.25 μM of the compound, plateauing the threshold to ~5 nM O/A at 5 μM FB231. Increasing doses of FB231 has no effect on the maximum mitophagy level. MTK458 was able to lower the threshold to a greater extent, dropping mitophagy induction threshold to 1.7 nM at 20 μM.

### FB231 and MTK458 potently activate the PINK1/Parkin pathway in combination with mitochondrial toxins

**Fig. 2.**
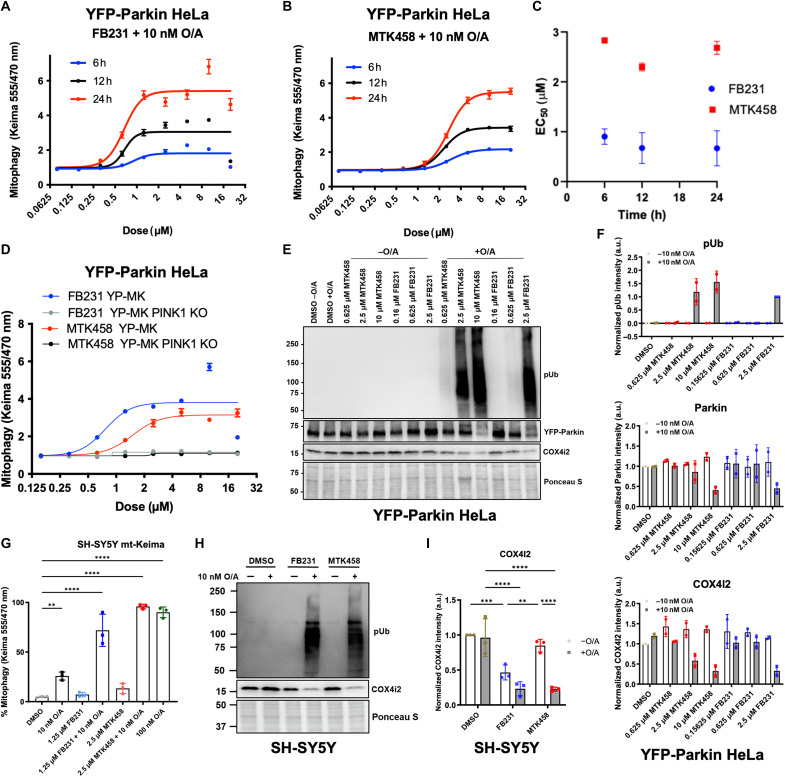
Parkin/PINK1 activators enable potent activation of the PINK1/Parkin pathway. (**A**) YFP-Parkin/mt-Keima–expressing HeLa cells were treated with O/A and varying doses of FB231, and the mt-Keima signals were measured at 6, 12, and 24 hours after treatment (*N* = 3). Solid lines represent fits to the Hill equation to determine EC_50_. (**B**) As in (A) with MTK458. (**C**) EC_50_ of FB231 and MTK458 are calculated for each time point (*N* = 3). Error represents the SD of residuals of each point from fits to the Hill equation. (**D**) As in (A) with YFP-Parkin/mt-Keima–expressing WT and PINK1 KO HeLa cells. (**E**) Immunoblots of mitophagy biomarkers pUb, YFP-Parkin, and COX4I2 in YFP-Parkin/mt-Keima–expressing HeLa cells treated with varying doses of FB231 or MTK458 with or without 10 nM O/A for 6 hours. (**F**) Normalized densitometry analysis of (E), (*N* = 2). (**G**) Flow cytometry analysis of mt-Keima–expressing SH-SY5Y cells treated with different combinations of O/A, FB231, and MTK458 for 24 hours (*N* = 3). Data represent the proportion of cells undergoing mitophagy as indicated by the ratio of mt-Keima 555/470 nM emission for at least 15 K cells. (**H**) Immunoblot of COX4I2 in SH-SY5Y cells treated with or without 10 nM O/A for 16 hours alone or in combination with 10 μM FB231 or 5 μM MTK458. (**I**) Normalized densitometry analysis of COX4I2 in (H), (*N* = 3). All cells were administered with 20 μM Q-VD-OPh to prevent cell death. Ponceau S stain was used as total protein loading control. Data are presented as means ± SD; **P ≤ 0.01, ***P ≤ 0.001, ****P ≤ 0.0001 [(G) one-way ANOVA; (I) two-way ANOVA]. a.u., arbitrary units.

Next, we sought to directly compare FB231 and MTK458 at a constant dose of O/A. We chose 10 nM O/A, which failed to induce mitophagy alone, but showed robust mitophagy when cotreated with the activators. We measured YFP-Parkin cells at 6, 12, and 24 hours after treatment with O/A and pan-caspase inhibitor QVD. We found that both compounds induced mitophagy in a dose-dependent and time-dependent manner (Fig. 2, A and B). FB231 demonstrated higher potency than MTK458, with an EC_50_ of 0.67 μM compared to 2.7 μM in MTK458 after 24 hours of cotreatment with 10 nM O/A (Fig. 2C). Both compounds showed similar time-dependent mitophagy activation. At the highest doses tested (10 and 20 μM), FB231 appeared to deviate from the expected response and reduce mitophagy at 20 μM. These results suggest a potential off-target effect not seen in MTK458.

To confirm that MTK458 and FB231 act through the PINK1/Parkin pathway, we tested whether both compounds would induce mitophagy in YFP-Parkin–expressing PINK1 knockout (KO) cells. We found that PINK1 KO abolished MTK458/FB231-induced mitophagy, indicating that the mitophagy-inducing effects of both compounds are strictly dependent on the PINK1/Parkin pathway (Fig. 2D).

To biochemically confirm PINK1/Parkin activation, we performed a Western blot for phospho-S65–ubiquitin (pUb). pUb is a readout of both PINK1 and Parkin activation and has been reported to be a potential biomarker of PD (*30*). We treated cells with and without 10 nM O/A and/or in combination with varying doses of FB231 and MTK458 and blotted for pUb. Ten nanomolar O/A alone and either activator compound alone demonstrated no pUb signal at any dose tested, whereas cotreatment of O/A with FB231 or MTK458 induced robust pUb accumulation (Fig. 2E). We also blotted for Parkin, which is both autoubiquitinated and degraded during the process of mitophagy. We observed increases in a higher molecular weight Parkin band and loss of Parkin signal upon MTK458/FB231 cotreatment with O/A. In addition, we observed loss of the inner mitochondrial complex IV subunit 4 isoform 2 (COX4I2) upon cotreatment with MTK458/FB231 and O/A in a dose-dependent manner (Fig. 2, E and F).

These blots independently confirm the activation of PINK1/Parkin as the source of mitophagy in the mt-Keima assay. Loss of COX4I2 could be partially prevented by the addition of the V-ATPase inhibitor bafilomycin A, which inhibits degradation by the lysosome in the final step of autophagy (fig. S3, A and B). Having shown that FB231 and MTK458 could induce mitophagy in the YFP-Parkin–expressing HeLa cell line, we sought to determine whether this effect was conserved in a cell line expressing endogenous levels of Parkin. SH-SY5Y is a neuroblastoma cell line often used as a PD model since they display some neuronal phenotypes and can be induced to produce tyrosine hydrolase, a marker of dopaminergic neurons (*44*). They naturally express Parkin and undergo PINK1/Parkin-dependent mitophagy in response to mitochondrial damage (*45*). We first tested whether FB231 or MTK458 could induce mitophagy in these cells upon cotreatment with O/A. We measured mitophagy using mt-Keima analyzed by flow cytometry. We treated cells with 1.25 μM FB231 or 2.5 μM MTK458 with or without 10 nM O/A for 24 hours; we also tested 100 nM O/A as a positive mitophagy control. As in YFP-Parkin–expressing HeLa cells, FB231 alone had no effect on mitophagy. We observe a slight induction of mitophagy upon MTK458 alone, or 10 nM O/A alone, compared to dimethyl sulfoxide (DMSO)–treated samples (Fig. 2G). Combination of either drug with 10 nM O/A led to a robust mitophagy signal. Next, we treated SH-SY5Y cells with 10 μM FB231 or 5 μM MTK458 with or without 10 nM O/A for 16 hours. Blotting for pUb, we found that FB231, MTK458, and 10 nM O/A alone had no signal, whereas combination of O/A with either FB231 or MTK458 resulted in a strong pUb signal as well as loss of the inner mitochondrial membrane protein COX4I2 (Fig. 2, H and I).

### FB231 and MTK458 can synergistically enhance mitophagy

**Fig. 3.**
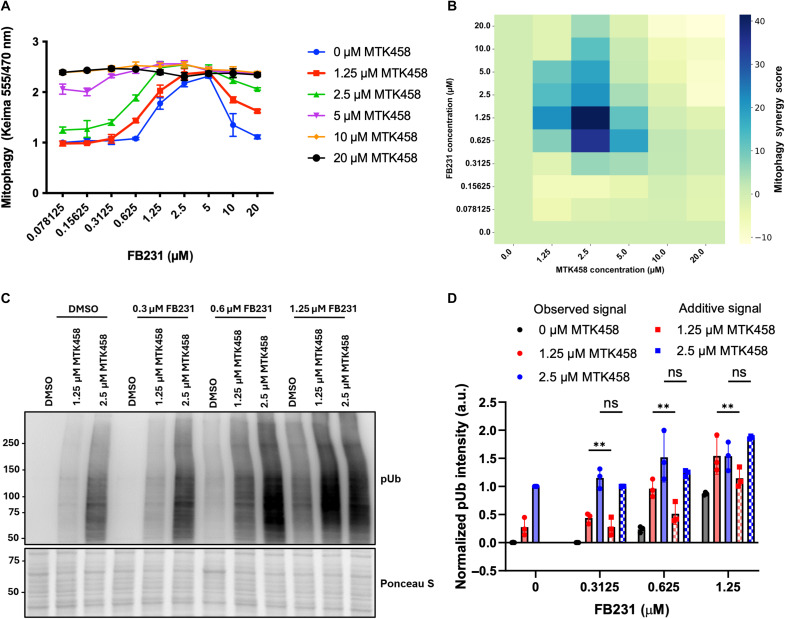
FB231 and MTK458 can be used to synergistically enhance mitophagy. (**A**) YFP-Parkin/mt-Keima–expressing HeLa cells were treated with 10 nM O/A and varying combinatorial doses of FB231 and MTK458 for 6 hours, whereupon mt-Keima signal was measured. Each combinatorial dose is represented as colored points connected with solid lines. Three cell wells are measured for each point. (**B**) ZIP Synergy score calculated for each dose of FB231 and MTK458 from (A). Positive synergy (blue) peak is observed in the region around 1.25 μM FB231 and 2.5 μM MTK458. Negative synergy (yellow) is observed at the highest doses of FB231. (**C**) YFP-Parkin/mt-Keima HeLa cells were treated with combinatorial doses identified in (B) to induce synergy for 6 hours and analyzed via Western blot. Western blotting detected pUb for cells treated with varying doses of FB231 or MTK458 and with or without 10 nM O/A. Ponceau stain is used as a total protein loading control. (**D**) Normalized densitometry analysis of (C). Solid colors represent measured pUb signal compared to the additive signal expected if the combination of the compounds were purely additive (checkered). Data in (D) are means ± SD from three independent experiments; **P ≤ 0.01 (two-way ANOVA).

PINK1/Parkin-modulating compounds have been developed with the goal of creating disease-modifying treatments for PD. A critical aspect of moving these compounds to the clinic is the ability to selectively target these pathways without inducing off-target effects. Minimizing the effective therapeutic dose of such compounds minimizes the chance that these compounds induce side effects due to lower-affinity interactions with off-target proteins. Both MTK458 and FB231 were designed to target the PINK1/Parkin pathway at distinct steps. We hypothesized that cotreatment could achieve an additive or potentially synergistic effect. To test this hypothesis, we performed a dose-combination screen of FB231 and MTK458 to identify potential synergistic regimes in the mt-Keima assay at 10 nM O/A. We found that certain combinations of FB231 and MTK458 produced exceptionally strong mitophagy induction (Fig. 3A). We performed a synergy analysis using the SynergyFinder tool with the zero-interaction potential (ZIP) model of synergy. The ZIP model fits a four-point logistic equation to the dose response data for each compound alone. The synergy score readout describes the percentage of activity that cannot be attributed to a linear combination of each compound at a measured dose. We observed an area of positive synergy between 0.625 to 1.25 μM FB231 and around 2.5 μM MTK231, with a max synergy score of 40, indicating that 40% of the effect size was beyond a linear combination of either drug’s effect (Fig. 3B). Concentrations of FB231 above 5 μM in combination with MTK458 above 5 μM were found to be antisynergistic. These results indicate that both compounds can be used in parallel to synergistically enhance mitophagy but suggest caution in the limits of this approach.

We subsequently measured levels of pUb at the synergistic regime via Western blot. (Fig. 3C). We found that, as in the mt-Keima assay, combinations of MTK458 and FB231 enhanced the pUb signal. To estimate synergy, the observed pUb signal was compared to the expected output if the individual pUb signals at the corresponding activator concentrations were added. Synergy in the pUb signature was observed at 0.625 μM FB231 and 1.25 μM MTK458, slightly lower in dosage than the regime observed in the mt-Keima assay (Fig. 3D). These data provide additional evidence for synergy between FB231 and MTK458 and suggest that they enhance mitophagy through distinct mechanisms.

### FB231 alone does not activate mitophagy upon artificial recruitment of Parkin to the mitochondria

One possibility to consider is that FB231 fails to induce mitophagy on its own, since active Parkin is not intrinsically localized to the mitochondria where it can ubiquitinate substrates to trigger mitophagy. To assess this possibility, we sought to reroute Parkin to the mitochondria and check whether addition of FB231 could induce mitophagy. We used the FKBP-FRB chemically induced dimerization system to recruit FKBP–green fluorescent protein (GFP)–tagged Parkin to FRB-tagged mitochondria in combination with either a dose titration of FB231 or O/A (fig. S4A). FKBP-FRB dimerization has been previously used to induce mitophagy through recruitment of various autophagic factors (*26*). We treated cells with an ethanol control or the dimerizing drug rapalog to induce FKBP-Parkin recruitment to the mitochondria. The success of this system was confirmed by observing colocalization of GFP with the mt-Keima reporter (fig. S4B). FB231 failed to induce significant increases in mitophagy with or without rapalog at any dose, although a slight positive trend in mitophagy was observed at doses above 5 μM (fig. S4C). Rapalog-induced Parkin recruitment to the mitochondrial surface failed to reduce the threshold for O/A-induced mitophagy in the dosing regime tested in this assay as it did not alter the levels of mitophagy observed at any dose of O/A (fig. S4D). These data indicate that FB231 is not capable of inducing mitophagy on its own, regardless of Parkin localization.

### Proteomic analysis identifies induction of the ISR by FB231 and MTK458

**Fig. 4.**
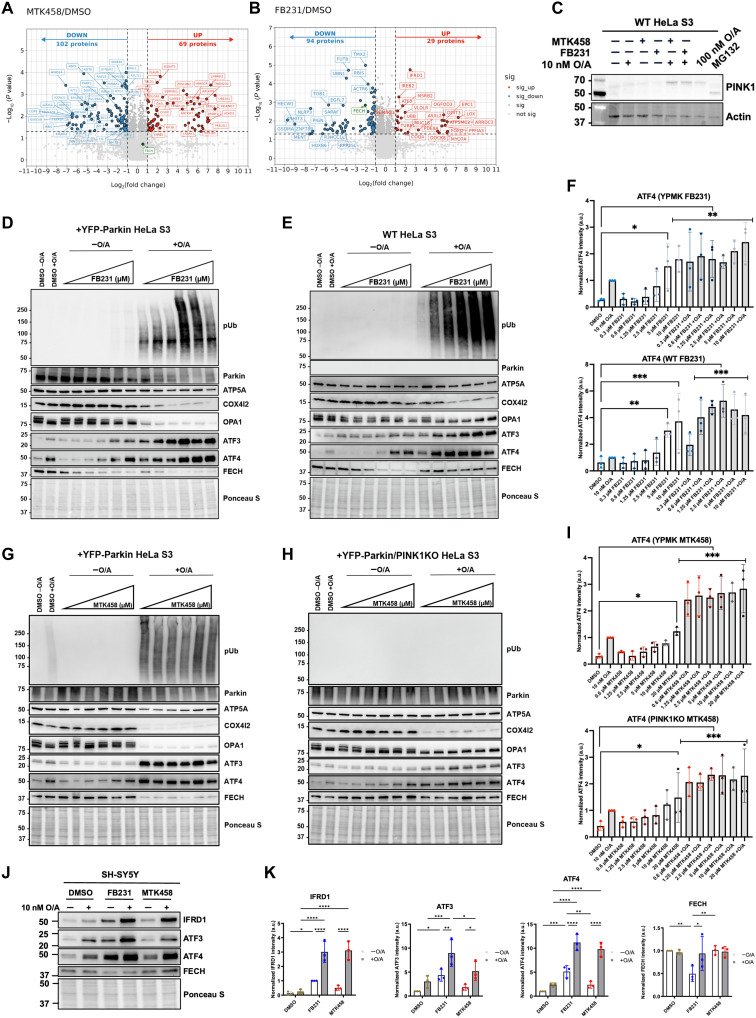
Label free proteomics identifies the ISR as off-target effects of FB231 and MTK458. (**A** and **B**) Volcano plots of label-free proteomics of YFP-Parkin–expressing HeLa cells treated for 12 hours with DMSO, (A) 20 μM MTK458, or (B) 10 μM FB231. Proteins with lower expression levels compared to DMSO (log_2_-fold-change < −1, *P* < 0.05) in blue; proteins with higher expression compared to DMSO (log_2_-fold-change >1, *P* < 0.05) in red. (**C**) Immunoblot of PINK1 in WT HeLa cells treated with various combinations of O/A, FB231, MTK458, and MG132 proteasome inhibitor for 16 hours. β-Actin was used as protein loading control. (**D**) Immunoblots of proteins involved in mitophagy, mitochondrial stress, and ISR in YFP-Parkin–expressing HeLa cells treated with 10 nM O/A and 0.3 to 10 μM FB231 for 16 hours. (**E**) As in (D) with WT HeLa cells. (**F**) Normalized densitometry analysis of ATF4 ISR biomarker in (D) and (E), (*N* = 3). (**G**) As in (D) with 0.6 to 20 μM MTK458. (**H**) As in (G) for YFP-Parkin/PINK1KO HeLa cells. (**I**) Normalized densitometry analysis of ATF4 in (G) and (H), (*N* = 3). (**J**) Immunoblots of IFRD1, ATF3, ATF4, and FECH in WT SH-SY5Y cells treated with 10 nM O/A, 10 μM FB231, and 5 μM MTK458 for 16 hours. (**K**) Normalized densitometry analysis of (J), (*N* = 3). All cells were administered with 20 μM Q-VD-OPh to prevent cell death. Ponceau S stain was used as a total protein loading control, unless stated otherwise. Data are presented as means ± SD; *P ≤ 0.05, **P ≤ 0.01, ***P ≤ 0.001, ****P ≤ 0.0001 (two-way ANOVA).

Reduction of mitophagy at high doses of FB231 suggests potential detrimental effects in cells (Fig. 2A). A slight increase in mitophagy was observed in SH-SY5Y cells treated with MTK458 alone (Fig. 2G). We performed whole-cell label-free proteomics to explore possible off-target effects in YFP-Parkin–expressing HeLa cells treated with 20 μM MTK458 or 10 μM FB231 (Fig. 4, A and B). The effects of MTK458 appeared widespread with no clear altered pathway evidence by the lack of significant Gene Ontology (GO) terms. However, perturbations in mitochondrial proteins including loss of COA5, HIGD1A, and up-regulation of fatty acid metabolism genes ACADS (Fig. 4A). FB231 alone induced a stress response–like signature that included the up-regulation of ATF3, IFRD1, and VLDLR (Fig. 4B) (*46*). Moreover, there was up-regulated iron response element–binding protein 2 (IREB2) and down-regulated iron-related mitochondrial protein ferrochelatase (FECH), suggesting a potential off-target effect on iron-related pathways based on GO molecular function term analysis (fig. S5) (*47*–*49*).

We validated a subset of these hits in an orthogonal manner using Western blotting. We treated both YFP-Parkin–expressing cells and the background WT HeLa cells (which do not express any endogenous Parkin) with 10 μM FB231, 5 μM MTK458, with or without 10 nM O/A. We found that FB231 alone led to induction of ATF3 and its activator in the ISR, ATF4, as well as IFRD1 (fig. S6, A to D). FB231 treatment alone also led to loss of FECH in WT and YFP-Parkin cells. These results confirm that FB231 indeed activated the ISR and perturbed an iron-dependent pathway. Addition of O/A alone also induced the induction of ISR and loss of FECH in YFP-Parkin HeLa cells. This effect was enhanced by cotreatment with either mitophagy activator.

Given the lack of Parkin in WT HeLa cells, these data indicate that the effects of FB231 on the ISR and iron-related pathway response are independent of Parkin modulation. We therefore blotted for PINK1 in WT HeLa cells and found that in combination with 10 nM O/A, both FB231 and MTK458 lead to the stabilization of full-length PINK1 (Fig. 4C). Although Parkin is the intended target of FB231, these data suggest that HeLa cells experience mitochondrial stress (as reflected by PINK1 stabilization) upon FB231 treatment, regardless of whether Parkin is present or not.

To determine whether the effect of MTK458 and FB231 on the ISR was independent of PINK1 activation and whether there is a therapeutic window in which mitophagy could be enhanced while minimizing the ISR, we tested both FB231 and MTK458 in a six-point dosing curve alone or in the presence of O/A. We performed this experiment in YFP-Parkin–expressing HeLa, WT HeLa, and YFP-Parkin/PINK1 KO Hela. In YFP-Parkin–expressing WT cells, cotreatment of O/A with FB231/MTK458 resulted in depletion of ATP5A, a biochemical readout for mitochondrial mass (Fig. 4, D and G). Loss of ATP5A was blocked by loss of Parkin or PINK1 (Fig. 4, E and H, and fig. S7, A and B), indicating that mitophagy is dependent on the PINK1/Parkin pathway. Notably, FB231 induced pUb accumulation in WT HeLa cells that lack Parkin, indicating that FB231 is activating PINK1 (Fig. 4E). Consistent with the idea that PINK1 is the only known Ub kinase, both compounds failed to induce pUb signal in PINK1 KO cells (Fig. 4H and fig. S7A).

We found other evidence of cellular stress upon treatment with these compounds. Both FB231 and MTK458 greatly enhanced the cleavage of the stress-sensitive mitochondrial fusion protein, OPA1, into lower molecular weight isoforms in the presence of 10 nM O/A (Fig. 4, D to H, and fig. S7, A to B). These effects were found in all tested HeLa cell lines, regardless of PINK1 or Parkin expression. OPA1 cleavage is indicative of mitochondrial stress and can be induced by a wide range of mitochondrial toxins (*50*). At this same dosing regime, we observe induction of the ISR protein ATF4 and its downstream target ATF3 (Fig. 4, F and I). We observed induction of the ISR proteins within 6 hours of treatment with activators and 10 nM O/A (fig. S6, E and F). Notably, the induction of ATF3/4 appeared enhanced in the YFP-Parkin HeLa cells. We wondered whether this observation could indicate that PINK1 activation by MTK458 could explain at least part of the ISR induction. We tested this by using higher doses of O/A in YFP-Parkin–expressing HeLa, WT HeLa, and YFP-Parkin/PINK1 KO Hela (fig. S7C). We found that loss of PINK1 or Parkin attenuated the ISR response as measured by ATF3 accumulation after 16 hours (fig. S7D). These data indicate that the PINK1/Parkin pathway can enhance the ISR response induced by mitochondrial toxins, and this effect was not unique to FB231 or MTK458.

We also sought to confirm whether the effects on the ISR and iron-related pathways were conserved in the endogenous Parkin-expressing SH-SY5Y cells. Blotting for ATF3, ATF4, and IFRD1, we found that all three proteins were up-regulated upon FB231 treatment alone (Fig. 4, J and K). O/A treatment enhanced the expression of these stress markers. As in WT HeLa, MTK458 in combination with O/A enhanced the expression of ISR markers ATF4 and IFRD1 compared to O/A treatment alone (Fig. 4, J and K). This confirms the finding that MTK458 may enhance the ISR response upon cotreatment with low doses of O/A. Treatment with FB231 alone reduced expression of FECH, but this was partially mitigated upon cotreatment with O/A (Fig. 4, J and K). This may be due to stimulation of FECH expression upon O/A treatment in this cell type.

Together, these markers indicate the induction of a potent mitochondrial stress, leading to the activation of the ISR at or below the doses of the compounds that we and other groups have observed to induce mitophagy. These data indicate that FB231 and MTK458 both may act as weak mitochondrial toxins independently of Parkin or PINK1.

### Mitophagy activators trigger the DELE1/HRI-dependent ISR independent of PINK1/Parkin

**Fig. 5.**
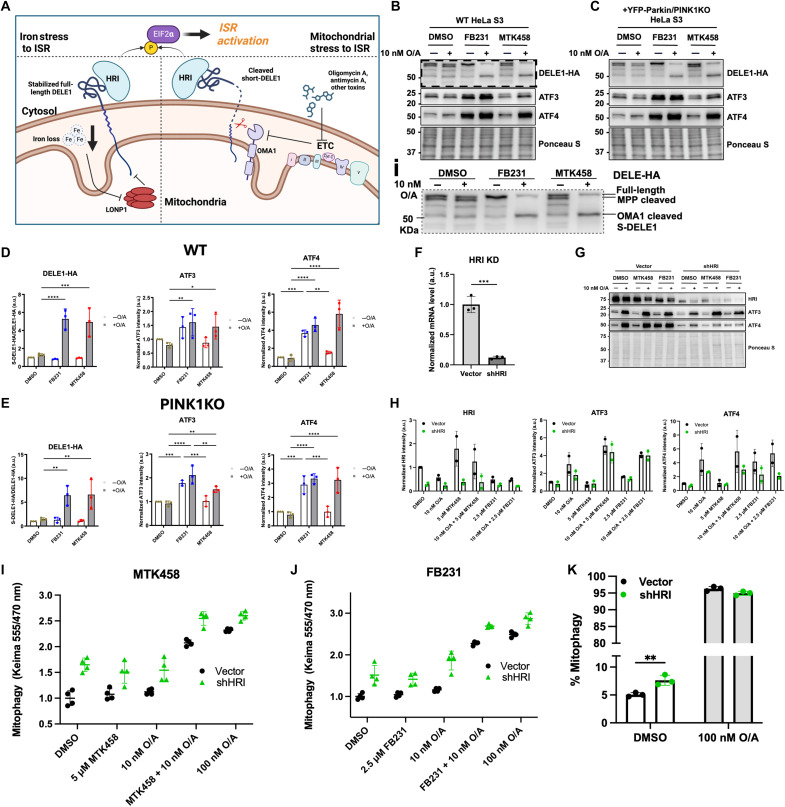
FB231 and MTK458 induce mitophagy independently of the ISR. (**A**) Diagram detailing the activation of the ISR upon exposure to mitochondrial toxins. (**B** and **C**) Immunoblots of ISR biomarkers in (B) WT or (C) YFP-Parkin/PINK1KO HeLa cells treated with 10 nM O/A, 10 μM FB231, and 10 μM MTK458. Inset (i) depicts full-length, MPP-cleaved, and OMA1-cleaved DELE1-HA in (B). (**D** and **E**) Normalized densitometry analysis for (B) and (C), respectively (*N* = 3). (**F**) Effectiveness of short hairpin RNA (shRNA) KD of HRI by quantitative polymerase chain reaction analysis. (*N* = 3). (**G**) Immunoblots of ISR biomarkers in YFP-Parkin–expressing HeLa cells expressing an HRI-targeting shRNA or the same plasmid vector lacking the shRNA-producing region treated with 10 nM O/A, 2.5 μM FB231, or 5 μM MTK458 with and without O/A for 16 hours. (**H**) Normalized densitometry analysis of integrative stress response biomarkers in (G), (*N* = 2). (**I** and **J**) mt-Keima assay of HRI KD YFP-Parkin/mt-Keima–expressing HeLa cell lines treated with O/A and (I) MTK458 or (J) FB231 for 6 hours (*N* = 4). (**K**) Flow cytometry analysis of mt-Keima–expressing HeLa cells treated with DMSO or O/A for 24 hours (*N* = 3). Data represent the proportion of cells undergoing mitophagy as indicated by the ratio of mt-Keima 555/470 nM emission for at least 30 K cells. All cells, except in (C), were administered with 20 μM Q-VD-OPh to prevent cell death. Ponceau S stain was used as a total protein loading control. Data are presented as mean ± SD; *P ≤ 0.05, **P ≤ 0.01, ***P ≤ 0.001, ****P ≤ 0.0001 [(D), (E), and (K): two-way ANOVA; (F) Student’s *t* test.

It is now known that mitochondrial stress can be relayed to the cytosol by the recently described DELE1-HRI pathway, converging on activation of EIF2a (*51*, *52*). In this pathway, the short-lived mitochondrial protein DELE1 can be activated either by cleavage by OMA1, allowing it to be released into the cytosol, or by iron loss, which stabilizes the full-length form of DELE1 at the OMM, analogous to PINK1 stabilization (*53*). In both cases, DELE1 can activate HRI in the cytosol to phosphorylate EIF2a, thereby turning on the ISR through global translational repression and selective translation of ATF4 (Fig. 5A). We sought to determine whether ISR activation by FB231 and MTK458 is mediated by the DELE1-HRI pathway. We first blotted for endogenous DELE1 in a previously developed 293T line containing endogenous HA-tagged DELE1 with combinations of MTK458 and FB231 with and without 10 nM O/A (fig. S7E) (*54*). We observed the presence of cleaved, lower–molecular weight form of DELE1 upon cotreatment with MTK458/FB231 and 10 nM O/A. Given the low signal-to-noise of these endogenous DELE1 blots, we next transiently expressed DELE1-HA in WT HeLa cells and treated cells with MTK458/FB231/10 nM O/A combinations for 16 hours. We now observed the presence of multiple DELE1-HA bands (Fig. 5B). FB231 alone predominantly lead to the accumulation of full-length DELE1 previously associated with iron depletion [Fig. 5B, inset (i)]. Upon cotreatment of MTK458 or FB231 with 10 nM O/A, we observed the additional accumulation of the lower molecular weight, presumably OMA1-cleaved, DELE1 species and ATF3/4 accumulation (Fig. 5, B and D). We next performed this experiment in the YFP-Parkin/PINK1 KO HeLa cells and observed identical results, confirming independence of this phenomenon from the PINK1/Parkin pathway (Fig. 5, C and E). Our work and recent preprints have suggested that the DELE1-HRI pathway may in some cases reduce PINK1-driven mitophagy and enhance other forms of mitophagy (*55*–*57*). We knocked down HRI to confirm that this pathway was responsible for ISR induction and whether it had any effect on the mitophagy observed (Fig. 5F). We found that knockdown (KD) of HRI attenuated ATF4/ATF3 induction by FB231 alone and by FB231/MTK458 in combination with 10 nM O/A (Fig. 5, G and H). We found that mitophagy induction by FB231 and MTK458 was unaffected by HRI KD, indicating that ISR induction is discrete from mitophagy induction in this context (Fig. 5, I and J). However, we observed some increases in basal levels of mitophagy upon HRI KD (Fig. 5K).

### FB231 induces mitophagy through an iron chelation–based mechanism

**Fig. 6.**
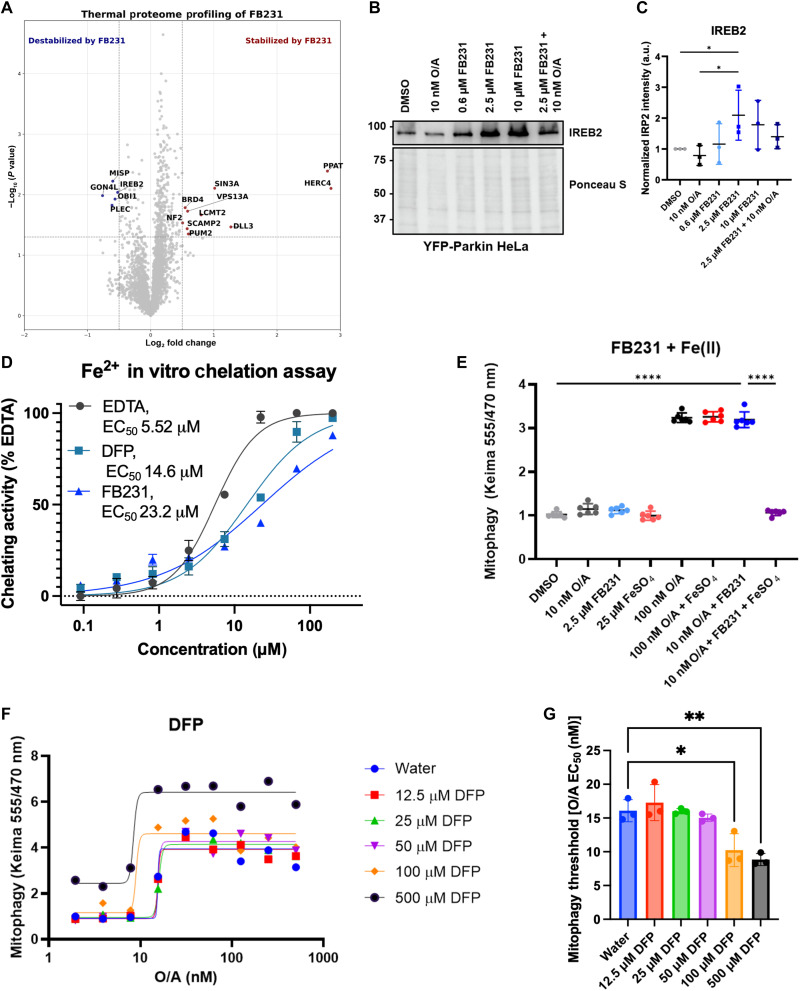
FB231 induces mitophagy through an iron-chelation–based mechanism. (**A**) Volcano plot of TPP of YFP-Parkin–expressing HeLa cell lysate treated with 40 μM FB231 compared to equivalent DMSO-treated lysate. Significantly destabilized proteins compared to DMSO, with fold change < −1 and *P* < 0.05 indicated in blue. Proteins significantly stabilized compared to DMSO, fold-change >1 and *P* < 0.05 indicated in red. Gene names of significantly altered proteins are labeled. (**B**) Immunoblots of iron-sensitive protein IREB2 in YFP-Parkin–expressing HeLa cells treated with various doses of FB231 or in combination with 10 nM O/A. (**C**) Normalized densitometry analysis of (B), (*N* = 3). (**D**) In vitro ferrous ion (Fe^2+^) chelation assay using colorimetric analysis of ferrozine. EDTA, DFP, and FB231 chelation ability is normalized to the maximum chelating activity observed for EDTA. Points represent means and SD (*N* = 3) for individual well measurements, and solid lines represent corresponding fits of the data to a four-parameter Hill equation use to determine the EC_50_. (**E**) mt-Keima–based mitophagy assays for YFP-Parkin HeLa cells treated alone or in combinations of FB231, 10 nM O/A, 25 μM FeSO_4_, and 100 nM O/A for 24 hours (*N* = 6). (**F**) mt-Keima–based mitophagy assay of YFP-Parkin/mt-Keima–expressing HeLa cells treated with varying doses of O/A and previously reported mitochondrial toxin DFP for 24 hours. (**G**) Mitophagy induction threshold, the EC_50_ of O/A for mitophagy, is calculated for each dose of DFP (*N* = 3). All cells were administered with 20 μM Q-VD-OPh to prevent cell death. Data are presented as means ± SD; *P ≤ 0.05, **P ≤ 0.01, ****P ≤ 0.0001 (one-way ANOVA).

Having determined that FB231 and MTK458 induce mitochondrial damage leading to the ISR and independent of PINK1, we sought to determine the direct target of these molecules using thermal proteome profiling (TPP). This technique uses mass spectrometry–based proteomics in cell lysate treated with high doses of a compound and subjected to a heat gradient. Proteins whose heat stability are altered by the compound directly or a change in binding partners can be detected by altered abundance, enabling unbiased proteome-wide discovery of potential drug targets. We performed TPP for FB231 and MTK458 in YFP-Parkin–expressing HeLa lysate. Parkin and PINK1 were not detected in any samples, and, therefore, no determination about direct binding could be made. MTK458 had no clear hits that could directly explain its effects on mitochondria (fig. S8). Top hits for FB231 stabilization included phosphoribosyl pyrophosphate amidotransferase, and the HECT-domain containing E3 ligase, HERC4 (Fig. 6A). The stabilization of HERC4 could indicate a general interaction of FB231 with HECT domain ligases, which, like Parkin, form a transient thioester intermediate with Ub (*58*). FB231 also leads to the destabilization of the iron responsive protein IREB2, which we had also found up-regulated upon FB231 treatment (Fig. 6, A to C). IREB2, also known as IRP2, is known to bind iron as a molecular glue that links it to E3 ligase FBXL5 (*59*). IREB2 can also be activated by iron loss independent of FBXL5 by an unknown mechanism (*60*). Confirming the mass spectrometry results, we found that treatment with FB231 lead to up-regulation of IREB2 in a dose dependent manner (Fig. 6, B and C). Ten nanomolar O/A was found to slightly reduce IREB2 accumulation. Thus, several observations suggested that FB231 may be acting as an iron chelator: the TPP and total proteome showing changes in IREB2, the loss of FECH, and the stabilization of full-length DELE1. We confirmed that FB231 could chelate Fe^2+^ ions using a ferrozine-based colorimetric assay (Fig. 6D). FB231 showed comparable chelation ability to the widely used chelator, deferiprone (DFP) (*61*). To test whether ferrous iron had any effect on FB231’s mitophagy induction, we cotreated 2.5 μM FB231 with or without 10 nM O/A and with the addition of 25 μM FeSO_4_. Mitophagy induction by FB231 + 10 nM O/A was completely blocked by iron supplementation (Fig. 6E). In contrast, iron had no effect on mitophagy induced by 100 nM O/A. These experiments strongly suggest that FB231’s mechanism of action is via chelation of iron. To test whether this was a general feature of iron chelators, we tested whether the stronger chelator DFP could alter the O/A-induced mitophagy threshold. High doses of DFP alone induces a distinct PINK1/Parkin-independent mitophagy (Fig. 6F). We found that 500 μM DFP induced moderate mitophagy, which could be enhanced by O/A. Cotreatment of 500 μM DFP with O/A led to increased mt-Keima signal beyond what was observed in O/A or DFP mitophagy alone, indicating an additive contribution from both mitophagy pathways (Fig. 6F). Upon treatment with O/A, we observe potent mitophagy induction at doses above 15 nM. Cotreatment of DFP with O/A significantly reduced the mitophagy induction threshold of O/A starting at 100 μM DFP (Fig. 6G). These findings demonstrate that an iron loss can potentiate the PINK1/Parkin pathway.

### FB231 and MTK458 inhibit mitochondrial function through distinct mechanisms

**Fig. 7.**
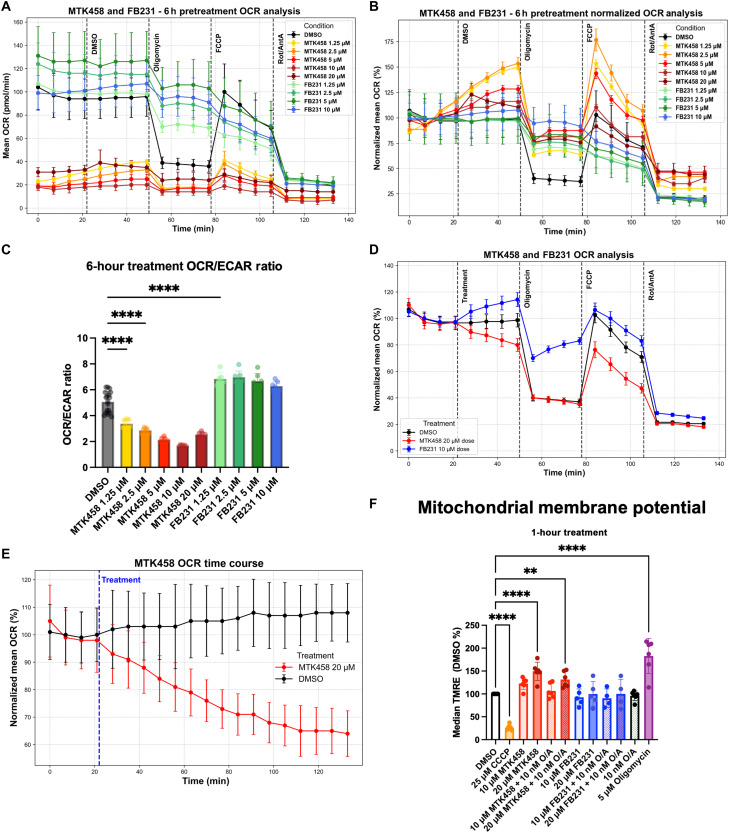
FB231 and MTK458 inhibit mitochondrial function through distinct mechanisms. (**A**) Raw Seahorse XF OCR plots for WT HeLa cells treated for 6 hours with increasing concentrations of MTK458 (yellow-orange-red), FB231 (green-blue), or DMSO control (black). Cells were treated with inhibitors at the time indicated by the vertical dotted line. (**B**) OCR plots in (A) normalized to the average of the first four time points for each treatment condition. Data are given as the % of the initial average. (**C**) Initial OCR/ECAR ratio for cells treated for 6 hours with varying doses of MTK458, FB231, or DMSO control. (**D**) Normalized OCR plot of WT HeLa cells treated in real time with MTK458, FB231, and DMSO followed by mitochondrial inhibitors at the indicated times (*N* = 4). (**E**) Normalized OCR plot of WT HeLa cells treated with either 20 μM MTK458 or DMSO and measured every 7-min for ~2 hours. (**F**) Flow cytometry analysis of MMP measured by TMRE median fluorescence in cells treated with indicated compound cocktails for 1 to 2 hours before analysis. Each colored dot represents the average of three cell-well replicates from at least four separate experiments. Dotted bars represent the addition of 10 nM O/A. All data from Seahorse experiments represents the means of at least four separate wells. Data are presented as means ± SD; **P ≤ 0.01, ****P ≤ 0.0001 (one-way ANOVA).

To explore the effect of MTK458 and FB231 on mitochondrial function, we used Seahorse extracellular flux assays to evaluate mitochondrial oxidative phosphorylation activity. With 6 hours of treatment, MTK458 caused strong reductions in the oxygen consumption rate (OCR), whereas the extracellular acidification rate (ECAR) was largely unaffected and resulted in a lowered OCR/ECAR ratio (Fig. 7, A to C). MTK458 reduced sensitivity to oligomycin, but cells were still affected by decoupler FCCP and electron-transport chain (ETC) inhibitors rotenone and antimycin A. In contrast, after treatment with FB231, OCR was elevated and showed less sensitivity to oligomycin and no sensitivity to FCCP (Fig. 7, A to C). We hypothesized that this may be explained by FB231 causing electron leak across the inner membrane.

The data for MTK458 were particularly unexpected since other groups have found MTK458 to have little to no effect on OCR, except at 25 μM MTK458 (*62*). In those experiments, OCR was measured for 20 min after drug injection. We found that MTK458 induced only a slight reduction in basal OCR within the 20-min observation period after drug injection (Fig. 7D).

Trends in MTK458 and FB231 appeared to approach data measurements observed in the 6-hour pretreatments (Fig. 7A). When we extended the measurements to 2 hours, we observed 20 μM MTK458 to slowly and progressively reduce the basal OCR rate compared to the untreated control, which remained stable (Fig. 7E). These results explain the apparent incongruity between our pretreatment regimen and the injection experiments performed by another group (*62*).

We next measured MMP using the potentiometric dye tetramethylrhodamine, ethyl ester, perchlorate (TMRE) via flow cytometry. Cells were treated for between 1 and 2 hours with varying combinations of the experimental drugs and 10 nM O/A, as well as depolarizer CCCP and oligomycin as minimum and maximum controls. We found that MTK458 led to significant hyperpolarization of MMP at 20 μM, which was partially dissipated by 10 nM O/A (Fig. 7F). FB231 had more subtle effects. We observed a slight, but insignificant, reduction of MMP. The notable result of MTK458 on MMP together with Seahorse data indicates that it may inhibit complex V. The slow kinetics of OCR reduction suggests that it is either a weak inhibitor or acts through an indirect mechanism. In these mitochondrial function experiments, we did not test the effects of MTK458 in PINK1 KO cells. It remains to be determined whether these effects are dependent on PINK1 status or not.

### FB231 and MTK458 reduce cell viability in the presence of mitochondrial stress

**Fig. 8.**
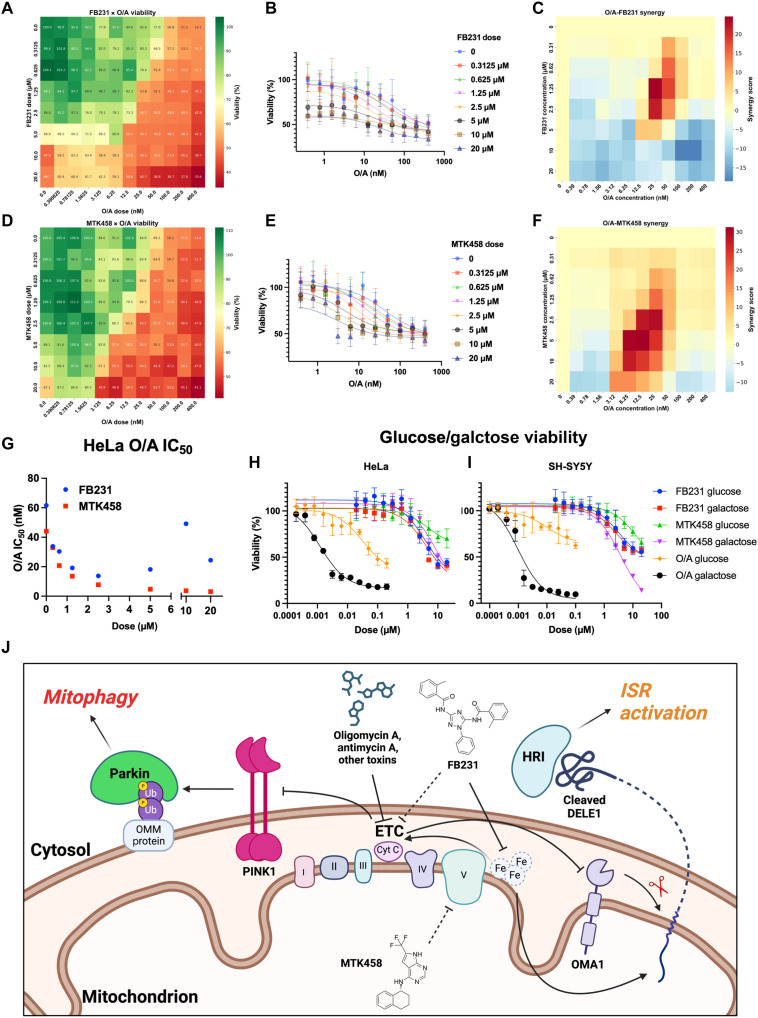
FB231 and MTK458 sensitize cells to mitochondrial stress. (**A**) Heatmap showing cell viability in WT HeLa cells treated with combinatorial doses of FB231 and O/A for 24 hours (*N* = 8). (**B**) WT HeLa viability dose response curves for O/A at increasing doses of FB231 (*N* = 8). Solid lines are fits to a Hill equation to determine the IC_50_. (**C**) Synergy score heatmap of O/A and FB231 combinations on cell viability. (**D**) As in (A) for cells treated with MTK458 and O/A. (**E**) As in (B) for MTK458 (*N* = 8). (**F**) As in (C) for MTK458. (**G**) Plot of calculated O/A viability IC_50_ at different doses of FB231 (blue) or MTK458 (red). (**H**) Dose-viability plot of WT HeLa cells in glucose-containing media or galactose-containing media treated with increasing doses of O/A, FB231, or MTK458 for 24 hours (*N* = 8). Solid lines represent fits the Hill equation. (**I**) As in (H) for WT SH-SY5Y cells (*N* = 8). (**J**) Diagram detailing the activation of PINK1/Parkin-mediated mitophagy and ISR upon exposure to various mitochondrial toxins. Data are presented as means ± SD.

Our evidence suggests that both compounds act independently of PINK1/Parkin to enhance O/A-induced mitophagy by acting as weak mitochondrial toxins. We next asked whether FB231 and MTK458 would enhance toxicity or protect cells from the effects of O/A. To assess this question, we cotreated various doses of FB231 or MTK458 with O/A for 24 hours and measured cell viability through the CellTiter-Glo assay in WT HeLa cells. Both FB231 and MTK458 alone caused loss of viability at high doses. FB231 was more toxic, resulting in ~50% viability at 10 μM and above (Fig. 8A). Significant toxicity in MTK458 alone was only observed at 20 μM, with a reduction to ~70% viability. Increasing doses of O/A led to 50% reduction in viability with a median inhibitory concentration (IC_50_) of 48 nM. Combinations of either FB231 or MTK458 with O/A enhanced viability loss, suggesting a potential synergistic interaction (Fig. 8, A to D). Using the ZIP synergy model, we found that both FB231 and MTK458 showed dosage regimes exhibiting positive synergy with O/A (Fig. 8, E and F). FB231 had a narrow synergistic regime between 25 and 100 nM O/A and centered at 1.25 μM FB231. At higher doses of FB231, negative synergy was observed. MTK458 demonstrated higher levels of synergy across a wide range of doses with peak synergy observed at 12.5 nM O/A and 5 μM MTK458. As another way to visualize the synergistic effect of these compounds on O/A’s potency in the context of cell viability, we calculated the O/A IC_50_ at each dose of either activator (Fig. 8G). Increasing the dose of FB231 or MTK458 corresponded to a lower O/A IC_50_. At the highest doses of FB231, this trend diverged as O/A was antisynergistic in this regime. Because CellTiter-Glo relies on cellular adenosine 5′-triphosphate (ATP) levels to quantify viability, we also imaged cells treated with FB231/MTK458 and O/A combinations with propidium iodide staining, observing similar synergistic regimes of cell killing (fig. S9, A and B). These results indicate that rather than protect cells from mitochondrial stress, both activators enhanced susceptibility to mitochondrial toxins.

It is well known that in conditions of high glucose used in normal cell culture media, cells can use glycolysis to provide most of their ATP needs, rendering them insensitive to mitochondrial defects. This is known as the Crabtree effect (*63*). Replacing glucose with galactose forces cells to rely on mitochondrial oxidative phosphorylation for ATP, effectively sensitizing them to mitochondrial damage. This effect was apparent upon O/A treatment, where a significant loss of viability and a reduced O/A IC_50_ was apparent in galactose media (Fig. 8H). MTK458 had been previously tested in the glucose/galactose assay in SK-OV-3 cells where enhanced susceptibility in galactose over glucose at high doses had been observed (*30*). In WT HeLa cells, we found that galactose significantly enhanced susceptibility to MTK458, resulting in ~40% viability in galactose compared to more than ~70% in glucose media at 20 μM. FB231 viability-dose response was virtually unchanged in galactose versus glucose media, suggesting that FB231’s cell killing function was not necessarily due to an inhibition of mitochondrial function. This result was similar to that seen for DFP (fig. S9C). We also performed the glucose/galactose assay in SY-SY5Y cells and found that they were more sensitive to MTK458 (Fig. 8I). To determine whether MTK458’s mitochondrial toxicity was related to a direct interaction with PINK1, we repeated the O/A dose-combination and glucose/galactose viability assays in the YFP-Parkin PINK1KO HeLa cell line. MTK458 showed potent synergy with O/A, and reduced cell viability was observed in galactose versus glucose (fig. S10, A to E). Together, these data suggest that both MTK458 and FB231 act as weak mitochondrial toxins, whose enhancement of mitophagy is due to a synergistic interaction with mitochondrial toxins such as O/A, independent of PINK1. Synergistic inhibition of mitochondrial function in combination with mitochondrial toxins leads to mitophagy through PINK1 stabilization and induction of the mitoISR through DELE1 cleavage and HRI activation (Fig. 8J).

## DISCUSSION

Cellular screens for mitophagy activators are plagued by hits that damage the mitochondria to induce mitophagy (*64*, *65*). Identifying lead compounds based on in vitro assays directly targeting PINK1 or Parkin promises to alleviate this problem. However, our work cautions that compounds with mild mitochondrial toxicity can nevertheless arise from such screens and appear to activate mitophagy.

We believe that this challenge arises from the unique dynamics of the PINK1/Parkin pathway. The PINK1/Parkin pathway displays potent switch-like behavior, which, at its root, is caused by the input threshold being dependent on PINK1 accumulation and a feed-forward step dependent on Parkin activation. The system avoids activation of mitophagy in response to minor fluctuations of mitochondrial function but potently clears mitochondria in conditions of sustained damage. The PINK1 accumulation threshold and feed-forward dynamics mean that the pathway is exquisitely sensitive to combinations of mitochondrial inhibitors. FB231 and MTK458 at their maximum doses do not inhibit mitochondrial function beyond the threshold of PINK1 accumulation needed to induce mitophagy, and, therefore, neither drug in isolation behaves as a mitophagy-inducing agent.

**Fig. 9.**
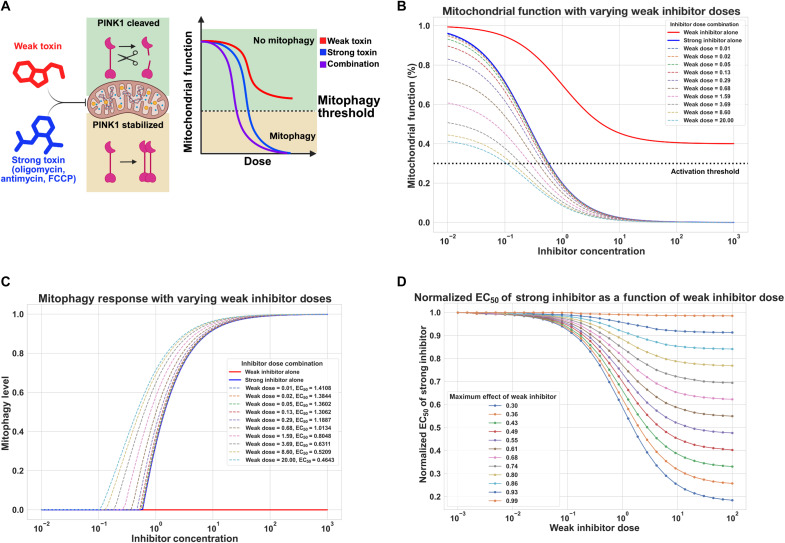
Mathematical modeling of mitochondrial inhibitors combinations on the induction of mitophagy. (**A**) Simplified model of mitochondrial toxin combinations altering mitochondrial function and mitophagy levels via PINK1 accumulation. (**B**) Mitochondrial function parameter as function of a weak inhibitor (red) with subthreshold mitochondrial toxicity, and a strong inhibitor (blue). The effect of the inhibitor is modeled as a Hill curve, with the response of combinations of inhibitors being multiplied together. Colored dashed curves represent the inhibition of mitochondrial function observed at a range of doses of the strong inhibitor, when cotreated with a constant dose of the weak inhibitor. (**C**) Apparent mitophagy as a function of inhibitor dose. Mitophagy is modeled as piecewise function dependent on the induction threshold. When mitochondrial function is above the threshold, mitophagy is set at 0. At or below the threshold, mitophagy is modeled as a power-law of the proportion of mitochondrial function below the threshold. The effect of the weak inhibitor is shown in red; the strong inhibitor in blue. Dashed curves represent the mitophagy dose response for the strong inhibitor at varying doses of weak inhibitor. The 50% mitophagy threshold (EC_50_) is reduced at increasing doses of the weak inhibitor. (**D**) The relative mitophagy induction threshold of the strong inhibitor, (EC_50_), is calculated as a function of the weak inhibitor dose. EC_50_ is normalized to that of the strong inhibitor alone. Each color represents the EC_50_ of the strong inhibitor calculated for the weak inhibitor’s maximum inhibitory effect on mitochondrial function.

We can use a simplified mathematical model to further rationalize how these subthreshold toxins can enhance mitophagy in the presence of a classical toxin or stressor (Fig. 9A). Each toxin inhibits a parameter we denote as “mitochondrial function.” Inhibition of mitochondrial function will not manifest as mitophagy until the level of PINK1 accumulation sufficient to recruit Parkin is breached; this occurs at the “mitophagy threshold.” At doses beyond the mitophagy threshold, mitophagy levels will be inversely proportional to the mitochondrial function but quickly saturate. In the model, the weak inhibitor causes limited damage to the mitochondria but never induces mitophagy (Fig. 9, B to C). A classical, or “strong inhibitor” potently inhibits mitochondrial function and results in mitophagy at a sufficient dose (Fig. 9B). When combined, there is an enhanced inhibition of mitochondria, and the dose of the strong inhibitor needed to induce mitophagy is reduced as a function of the weak inhibitor concentration (Fig. 9C). Despite the mild effects of the weak inhibitor alone, weak inhibitors can drastically alter the mitophagy induction threshold, or EC_50_, of the strong inhibitor (Fig. 9D). This model can reproduce the general features of the threshold data observed for FB231 and MTK458 (Fig. 1, C to E).

The totality of our results suggests that the ability of FB231 and MTK458 to enhance O/A-induced mitophagy is not due to direct activation of Parkin or stabilization of PINK1, respectively. FB231 induces mitochondrial disruption that is independent of Parkin expression, as evidenced by pUb accumulation, DELE1 accumulation, OPA1 cleavage, and COX4I2 loss in WT HeLa cells, which lack Parkin expression (Fig. 4, G and H). Moreover, these effects occur at doses around 1 μM, six times lower than the observed EC_50_ of FB231 on purified Parkin (Fig. 7, A and B, and figs. S7A and S1C). MTK458, in combination with low doses of O/A, similarly induces mitochondrial stress independently of PINK1, evidenced by enhancement of OPA1 cleavage and COX4I2 loss in PINK1 KO cells (Fig. 4, C and D). MTK458 has an inhibitory effect on oxygen consumption and sensitizes cells to both galactose and O/A, indicating clear mitochondrial toxicity (Figs. 7 and 8). The precursor to MTK458, kinetin, was initially thought to act as a potent substrate for PINK1, replacing ATP in the ATP-binding site and enhancing PINK1 activity (*66*). A recent study indicated that kinetin does not bind to *Pediculus humanus corporis* PINK1 or human PINK1 due to a steric clash, suggesting that kinetin does not enhance PINK1 kinase activity through the originally proposed mechanism (*67*). No effect on PINK1 phosphorylation activity was seen in a second study on isolated mitochondria (*36*). MTK458 alone has minimal effect on cell line growth, and slow inhibition of mitochondrial function in the Seahorse assay, explaining why MTK458 evaded detection as a mitochondrial toxin. In this study, we identify a mechanism of action (MOA) for MTK458, yet the direct target of MTK458 remains unclear.

Our study highlights an important challenge toward identifying activators of the PINK1/Parkin mitophagy pathway. We show that two putative activators have sufficient mitochondrial toxicity to plausibly explain their mitophagy-inducing properties when administered in conjunction with classical mitochondrial toxins. Our results suggest that development of more sensitive methods to detect subtle mitochondrial stress may be a potentially effective strategy for counter-screening mitotoxic compounds in future drug discovery campaigns. Both MTK458 and FB231 generate mitochondrial damage that stabilizes PINK1 and activates the more sensitive DELE1-HRI pathway. Moreover, ATF4 induction by MTK458 and FB231 was observed at doses below induction of mitophagy. High-throughput and sensitive ATF4 reporters have long been in use and represent another appealing strategy to identify subtle mitochondrial toxins (*68*).

A major question given our results clarifying the mechanism of action of MTK458 and FB231 is whether they may be clinically useful in PD. Given that mitochondrial toxins have been shown to directly cause and are associated with the development of PD, a weak toxin such as MTK458 or FB231 would be expected to have no benefit at best, or at worst, promote PD progression. DFP is another weak mitochondrial toxin that behaves similarly to FB231 and was previously shown to enhance the progression of PD in a large phase 2 clinical trial (*61*). However, the unique action of FB231 to induce an iron stress response at low doses suggests that it may be useful in other indications such as oncology. Given Parkin’s role as a cytoplasmic stress response sensor, it may be that mitochondrial damaging compounds can bind directly to Parkin as part of an activation sensing mechanism. In this case, many compounds that activate Parkin may also function as weak mitochondrial toxins, as we have seen in multiple screens that yielded diverse chemotypes.

An ideal mitophagy activator would enhance cell viability in response to mitochondrial toxins or genetic defects in mitochondrial function. A recent preprint demonstrates that a hypoxia-inducible factor 1α stabilizing drug, which induces mitophagy through the BNIP3/NIX receptor pathway, could protect neurons from mitochondrial toxins in PINK1/Parkin null cells (*69*). These results provide hope that alternative mitophagy inducers may yet demonstrate clinically relevant protection in PD.

Ultimately, this study demonstrates the ability of weak mitochondrial toxins to lower the mitophagy induction threshold by sensitizing the cell to mitochondrial stress. These data indicate the need for caution in screening for mitophagy activators in the presence of mitochondrial stressors because seemingly silent mitochondrial toxins may appear as promising pharmacological mitophagy potentiators, even if the initial compound hits were obtained using in vitro–purified enzyme targets in the absence of cells.

## METHODS

### Reagents

The following chemicals were used in this study: antimycin A (1397-94-0, USB Corporation), oligomycin A (HY-100558, MedChemExpress), Q-VD-OPh (HY-12305, MedChemExpress), FB231 (this paper), MTK458 (HY-152943, MedChemExpress), bafilomycin A (HY-100558, MedChemExpress), FuGENE 6 transfection reagent (E2691, Promega), Rapalog A/C heterodimerizer (635056, Takara Bio), and DFP (379409, Sigma-Aldrich). FB231 was synthesized by Wuxi App Therapeutics; detailed synthesis schema can be found in the Supplementary Materials.

### Cell lines

All cell lines were cultured at 37°C and 5% CO_2_. HeLa (RRID:CVCL_0058), SH-SY5Y (RRID:CVCL_0019), and human embryonic kidney (HEK) 293T (RRID:CVCL_0063) cells were obtained from the American Type Culture Collection (ATCC). YFP-Parkin/mt-Keima, YFP-Parkin/mt-Keima/PINK1KO, and FRB-Fis1/mt-Keima HeLa cells were supplied by the Youle laboratory. FKBP-GFP-Parkin/FRB-Fis1/mt-Keima HeLa and Mt-Keima SH-SY5Y cells were generated from this study. All cells were cultured in Dulbecco’s modified Eagle’s medium (DMEM, D6429, Sigma-Aldrich) supplemented with 10% (v/v) fetal bovine serum (FBS, F0926, Sigma-Aldrich) and 1% (v/v) penicillin-streptomycin (15140-122, Thermo Fisher Scientific). All cell lines were routinely tested for mycoplasma contamination.

### Transient transfection of plasmids

Transient transfection was achieved by using polyethylenimine Max 40 K (PEI, KyforaBio). A 3:1 ratio of PEI to DNA (w/w) was used to transfect cells with the plasmid of interest. The transfection media were then replaced with fresh media 16 hours posttransfection, at which point downstream experiments are performed. The following plasmid vectors were used for transfections in this study: pLVX-puro-DELE1-HA.

### Generation of stable cell lines

Stable cell lines were generated using lentiviral expression systems. For lentiviral transductions, HEK293T cells (RRID:CVCL_0063) were transfected with HGPM2, REV-1b, Tat-1b, VSV-G, and pHAGE constructs containing our gene of interest using FuGENE 6 transfection reagent (E2691, Promega). For retroviral transfection, HEK293T cells were transfected with pUMVC and VSV-G. The day after transfection, the culture medium was replaced with fresh media. Viruses were harvested 48 hour posttransfection and filtered through 0.45-μm syringe filters (WHA9914-2504, Cytiva) to avoid contamination of cultures with HEK293T cells. The cells were seeded into 12-well plates at 50,000 cells per well and infected via centrifugation by the virus-containing media supplemented with polybrene (8 mg/ml; Sigma-Aldrich). Cells having undergone successful infection were selected for by puromycin or neomycin, when the selection marker is present on the vector. The cells were then expanded and sorted by fluorescence-activated cell sorting (FACS) to additionally select for infected cells and normalize protein expressions across individual cells.

The following vectors were used in this study: pHAGE-FKBP-GFP-Parkin and pHAGE-mtKeima. For RNA interference, retroviral transduction was used to express short hairpin RNAs from the H1 promoter. The target sequences were HRI, 5′ GCATGAACCAAAC CCACTTCG 3′.

### Quantitative PCR

Total RNA was extracted from 800,000 cells using Direct-zol RNA MicroPrep kit (R2062, Zymo Research). One microgram of total RNA was used for cDNA synthesis via High-Capacity cDNA Reverse Transcription Kit (4368814, Thermo Fisher Scientific) for reverse transcription polymerase chain reaction (PCR) as per the manufacturer’s protocol. Quantitative PCR was done with primers for HRI (Forward: 5′-ACACCAACACATACGTCCAG-3′, Reverse: 5′-GCTCCATTTCTGTTCCAAACG-3′), and β-actin (Forward: 5′-TCATCACCATTGGCAATGAG-3′, Reverse: 5′-ACTTCATGATGGAGTTGAAG-3′), using Power SYBR Green PCR Master Mix (4368577, Thermo Fisher Scientific). Fold changes in RNA expression were calculated using the ΔΔCt method.

### Mitophagy experiments

Cells were treated with 10 nM each oligomycin A (HY-100558, MedChemExpress) and antimycin A (1397-94-0, USB Corporation) to induce mitochondrial damage. To induce mitophagy, varying doses of PINK1 activator MTK458 (HY-152943, MedChemExpress) and/or Parkin activator FB231 was added. In some experiments, as noted, we additionally added 20 μM Q-VD-OPh (HY-12305, MedChemExpress) to inhibit apoptosis. Samples were then analyzed by SDS–polyacrylamide gel electrophoresis and Western blot or mt-Keima assay.

### Imaging-based mt-Keima assay

HeLa cells stably expressing YFP-Parkin and mt-Keima (YPMK) were previously generated and used to measure induction of mitophagy. A HeLa cell line expressing mt-Keima and YFP-Parkin with PINK1 KO was used as a negative control. Both cell lines were plated at 15,000 cells per well in 96-well plates (655086, Greiner) and incubated overnight. The following day, compound dilutions were performed in a separate 96-well plate. To investigate the mitophagy induction threshold, compounds were serially diluted twofold to generate a six-dose titration, with final concentrations ranging from 0 to 20 μM for FB231 and MTK458, and 0 to 500 μM for DFP. A serial twofold dilution of an equimolar combination of oligomycin A and antimycin A was performed to generation a 10-dose titration, with final concentrations ranging from 0 to 500 nM. In each well, 190 μl of serum-free DMEM (Sigma-Aldrich) containing 50 μM Q-VD-OPh was added to 10 μl of compound dilutions in DMSO. Then, 12 μl of solutions from both the O/A and activator plate were added to the plated cells. Cells were incubated at 37°C/5% CO_2_ and then imaged at 6 hours via an ImageXpress Micro Confocal Plate Reader (Molecular Devices). The two channels used for imaging were excitation at 470 or 555 nm, with emission detected by a 610/20 filter. The average fluorescence intensity for each channel was calculated in the MetaXpress software of version 6.7.1.157 (Molecular Devices). Five images were taken and averaged for each well, and three separate plates, each representing independent experiments, were imaged to ensure reproducibility of data.

To experimentally obtain the EC_50_ of the mitophagy activators at 10 nM O/A, compounds were serially diluted twofold to generate a 10-dose titration (final concentration, 0 to 20 μM). In each well, 190 μl of serum-free DMEM (Sigma-Aldrich) containing 50 nM oligomycin A, 50 nM antimycin A, and 50 μM Q-VD-OPh was added to 10 μl of compound dilutions in DMSO. Then, 25 μl of these solutions was added to the plated cells, resulting in a final concentration of 10 nM oligomycin A, 10 nM antimycin A, and 10 μM Q-VD-OPh. The cells were imaged as described above at 6, 12, and 24 hours. Each dose was performed with six technical replicates. For the quantification of mt-Keima emission signals, the MetaXpress software (RRID:SCR_016654) was used. These values were plotted and subjected to statistical testing via GraphPad Prism.

### mt-Keima assay cells via flow cytometry

Stable cell lines expressing mt-Keima were seeded in 12-well plates at 200,000 cells per well and treated the following day with DMSO, 10 nM O/A, 1.25 μM FB231 alone or with 10 nM O/A, 2.5 μM MTK458 alone or with 10 nM O/A, and 100 nM O/A for 24 hours. The cells were then trypsinized and resuspended in sorting buffer [145 mM NaCl, 5 mM KCL, 1.8 mM CaCl_2_, 10 mM Hepes, 10 mM glucose, and 0.1% bovine serum albumin (BSA)]. The cells were then filtered through 35-μm strainer caps into 5-ml polystyrene FACS tubes (FSC-9005, Stellar Scientific) to eliminate large clumps of cells. Analysis was performed using CytExpert software version 2.6 on a Beckman Coulter CytoFLEX flow cytometer. Measurements of lysosomal mt-Keima were taken by calculating the ratio of mt-Keima emission at 610 nm after excitation by a 555-nm (pH 4) laser over mt-Keima emission at 610 nm after excitation by a 470-nm (pH 7) laser. For each sample, at least 30,000 events were collected and subsequently gated for live, single cells expressing mt-Keima. Flow cytometry data were analyzed using FlowJo version 10.10.0 (BD Biosciences)

### Animal experiments

Experiments with Sprague Dawley rats were done at BioDuro-Sundia (Shanghai, China). All animal experiments were reviewed and approved by IACUC (BD-201609126 Rats were obtained from Zhejiang Vital River Laboratory Animal Technology Co. Ltd., caged in static caging system fashion (three mice per cage) and fed experimental rat maintenance feed (Shanghai Protein Bio-Technology Limited, Shanghai, China) diet. During the study, the care and use of animals was conducted in accordance with the regulations of the Association for Assessment and Accreditation of Laboratory Animal Care (accreditation number is 001516).

### Western blotting

Cells were plated in six-well plates at a density of 400,000 cells per well and incubated overnight. Cells were harvested by trypsinization (12604-013, Thermo Fisher Scientific) and centrifugation. Cell pellets were washed with phosphate-buffered saline (PBS) and lysed in radioimmunoprecipitation assay (RIPA) buffer (50 mM tris, 150 mM NaCl, 0.1% SDS, 0.5% sodium deoxycholate, and 1% Triton X-100) supplemented by EDTA-free broad-spectrum protease inhibitors (Thermo Fisher Scientific). After incubating for 15 min on ice, the samples were spun down by centrifugation and soluble fractions were collected. Protein concentrations were normalized for equal loading. Five to ten micrograms of protein for each sample was resolved on 4 to 20% Mini-PROTEAN TGX gels (Bio-Rad) and then transferred to nitrocellulose membranes using the Trans-Blot Turbo Transfer System (Bio-Rad). Membranes were stained with Ponceau S for 5 min at room temperature (rt), washed with DI water, and then imaged using a ChemiDoc MP Imaging System (Bio-Rad). Membranes were washed in TBS-T (tris-buffered saline + 0.1% Tween 20) and then blocked at rt for 1 hour in TBS-T with 5% nonfat milk powder. Membranes were incubated overnight at 4°C with primary antibodies diluted in TBS-T with 5% milk. Membranes were washed three times with TBS-T and then incubated for 1 hour at rt with goat anti-mouse or goat anti-rabbit horseradish peroxidase (HRP)–conjugated antibodies (1706515 and 1706516, Bio-Rad) diluted in TBS-T with 5% nonfat milk powder. Membranes were washed three times with TBS-T and then imaged using Immobilon ECL Ultra Western HRP Substrate (WBULS0500, MilliporeSigma) and a ChemiDoc MP Imaging System (Bio-Rad). For the quantification of immunoblots, we performed densitometry analysis using ImageLab version 6.1 (RRID:SCR_014210).

To probe for endogenous PINK1, we followed a procedure from Thayer *et al.* (*70*). The cells were directly lysed in 1X sample buffer containing beta-mercaptoethanol (BME) and protease/phosphotase inhibitors. The samples were run on a 7.5% gel, transferred using the Bio-Rad system, and blocked in Licor PBS blocking buffer (LI-COR, 927-70001). Membranes were incubated in PINK1 primary antibody for 2 days at 4°C before secondary was added and membrane was imaged.

### Label-free proteomics

HeLa YFP-Parkin/mt-Keima cells were plated onto six-well plates at 750,000 cells per well and incubated overnight. Cells were then treated with 100 nM O/A, 5 μM MTK458, and 10 μM FB231 for 12 hours. The cells were harvested following treatment and then prepared using the EasyPep Mini MS Sample Prep Kit (A4006, Thermo Fisher Scientific) according to the manufacturer’s instructions. Peptide concentration was measured via the Pierce Quantitative Fluorometric Peptide Assay (catalog no. 23290, Thermo Fisher Scientific).

The liquid chromatography tandem mass spectrometry experiments were performed using an EASY-nLC 1000 (Thermo Fisher Scientific, San Jose, CA) connected to a QExactive HF mass spectrometer. The sample (1 μg) in 0.1% FA solution was loaded onto an Aurora UHPLC Column (25 cm by75 mm, 1.6 mm C18, AUR225075C18A, Ion Opticks) and separated over 136 min at a flow rate of 0.35 ml/min with the following gradient: 2 to 6% solvent B (7.5 min), 6 to 25% B (82.5 min), 25 to 40% B (30 min), 40e98% B (1 min), and 98% B (15 min). Solvent A consisted of 97.9% H_2_O, 2% ACN, and 0.1% formic acid, and solvent B consisted of 19.9% H_2_O, 80% ACN, and 0.1% formic acid. An MS1 scan was acquired in the Orbitrap at 120,000 resolution with a scan range of 350 to 1500 mass/charge ratio (*m*/*z*). The AGC target was 4 × 10^5^, and the maximum injection time was 50 min. Dynamic exclusion was set to exclude features after 1 time for 60 s with a 10-ppm mass tolerance. Higher-energy collisional dissociation fragmentation was performed with 35% collision energy after quadruple isolation of features using a 1.6-*m*/*z* isolation window, 5 × 10^4^ AGC target, and 35-ms maximum injection time. MS2 scans were then also acquired by the Orbitrap with 50,000 resolution. Ion source settings were as follows: ion source type, NSI; spray voltage, 2400 V; ion transfer tube temperature, 275°C. System control and data collection were performed by Xcalibur software. The mass spectrometry proteomics data have been deposited to the ProteomeXchange Consortium via the PRIDE partner repository with the dataset identifier PXD058150 (*71*).

### Thermal proteome profiling

TPP was performed following methods from the literature (*72*–*74*). Detailed methods and analysis of the TPP experiments can be found in the Supplementary Materials.

### Rapalog-induced chemical dimerization experiments

Chemically induced dimerization experiments were conducted using the Fis1-FRB and Parkin-FKBP system. Lentiviral transduction of FKBP-GFP-Parkin was performed on Fis1-FRB/mt-Keima HeLa cells procured from the Youle laboratory. The resulting stable cell line was then treated with A/C heterodimerizer (635056, Takara) for 24 hours. The cells were then analyzed by mt-Keima as described above.

### Cell viability assay

HeLa cells or SH-SY5Y were seeded at 400 cells per well in each well of a white 384-well plate (Greiner 781080). Thirty microliters of DMEM supplemented with glucose (4.5 mg/ml), 10% FBS, and 1% penicillin-streptomycin was used as the culture medium. In the galactose condition, 10 mM galactose replaced glucose in the medium. Following a 24-hour incubation at 37°C and 5% CO_2_, the cells were treated with the designated compound cocktail with DMSO concentration no more than 0.5% and four biological replicates per condition. The cells were incubated with compounds for 24 hours, and then cell viability was assessed either by the addition of 30 μl of CellTiter Glo reagent (G7572, Promega) or propidium iodide (1 μM). The cells were incubated for 10 min at rt, and luminescence/fluorescence was measured using a BioTek Synergy Neo Microplate Reader or high-throughput confocal. Cell viability for each well was calculated by comparing the percentage of viable treated cells to the DMSO control (i.e., 0 μM compound). IC_50_ values were then calculated from eight replicates collected in at least two separate experiments via nonlinear regression in GraphPad Prism 10.

### MMP measurements

WT HeLa cells were trypsinized and resuspended in complete media (DMEM supplemented with 10% FBS and 1% penicillin-streptomycin) containing 20 nM TMRE (#T669, Thermo Fisher Scientific). The cells were then filtered through 35-μm strainer caps into 96-well plates containing drug treatments dissolved in DMSO. The cells were kept shaking on an orbital shaker for 5 min before being allowed to incubate for 1 hour in the dark at rt. Analysis was performed using CytExpert software version 2.6 on a Beckman Coulter CytoFLEX flow cytometer. Measurements of membrane potential were taken using FL3. Three biological replicates were measured for each condition and for each experiment. For each sample, at least 12,000 events were collected and subsequently gated for live, single cells. Flow cytometry data were analyzed using Floread.io online analysis tool (https://floreada.io).

### In vitro iron chelation assay

Ferrous iron chelating assays using ferrous iron chelating assay kit (#AOX-15, AMSbio) according to manufacturer’s protocol. EDTA (from the kit), DFP (379409, Sigma-Aldrich), and FB231 stocks were diluted in assay buffer to make a nine-point threefold dilution dose series, with final volume of 100 μl. Controls with assay solution (no drug) and distilled water were used to determine maximum signal and background signal. One hundred microliters of working ferrozine solution was added and mixed on an orbital shaker for 10 min. Absorbances were read at 562 nm using a BioTek Synergy Neo2 plate reader (Agilent). Ferrous ion chelating was calculated as a % of the max signal observed in EDTA 200 μM condition: (%) = 100 × (*Abs*_max_ – *Abs*_test_)/*Ab*s_max_. Three replicate wells were measured for each condition.

### Seahorse extracellular flux measurements

A total of 20,000 WT HeLa cells were plated onto a 96-well plate 24 hours before the Seahorse experiment in complete media (DMEM supplemented with 10% FBS and 1% penicillin-streptomycin). Six hours before the experiment, the cells were washed into Seahorse media (Sigma-Aldrich; #D5030 supplemented with glutamine, sodium pyruvate, penicillin-streptomycin, and 25 mM glucose). For pretreatment experiments, the cells were treated with compound doses diluted in Seahorse media. One hour before analysis, the cells were transferred to a 37°C incubator with ambient CO_2_. The mitochondrial stress test was performed using a Seahorse Biosciences Extracellular Flux Analyzer (model XF96). Oligomycin (5 μM) was added to inhibit complex V, 1 μM FCCP was added to uncouple the proton gradient, and 5 μM rotenone/antimycin A was added to inhibit complex I/III. For drug injection experiments, either DMSO or the experimental drug was injected before the mitochondrial stress test. For extended observation of drug treatments, either DMSO or the experimental drug was injected and measurements were recorded every 7 min for 2 hours.

### Synergy score

Synergy scores were calculated using the SynergyFinder web server (*75*). Mitophagy scores such as the DMSO-treated condition was set to 0 and the max mitoKeima 555/488-nm value was set to 1 in each experiment. Viability data were normalized to the untreated condition in each experiment. Curves were fitted with the LL4 method, and outliers were not excluded. The ZIP model of synergy was used.

### Autoubiquitination

Parkin autoubiquitination was assayed using a preincubation of 196 nM FL-Parkin, 196 nM pUb, 196 nM Ub, and compound or DMSO control (to a maximum of 1% DMSO) for 15 min in reaction buffer [50 mM Hepes, 50 mM NaCl, 800 mM potassium fluoride, 0.005% Tween20, and 0.1% PF-127, (pH 8.5)], followed by the addition of a master mix of 5 nM E1 (Boston Biochem E-305), 50 nM UbcH7 E2 (Boston Biochem E2-640), and 8.8 nM ubiquitin-Europium (CisBio 61UBIKLA) in reaction buffer and incubation for an additional 120 min at 23°C, in a total reaction volume of 10 μl. Anti–6His-d2 (CisBio 61HISDLA) was added in detection buffer (reaction buffer plus 5 mM EDTA) for 120 min at rt, followed by reading plates on PerkinElmer Envision with top mirror: LANCE/DELFIA Duel/Bias, emission filter APC665 EM, and 2nd emission filter Europium 615 EM, reading 655 (channel 1) and 615 nm (channel 2) wavelengths on Envision, with Homogeneous Time-Resolved Fluorescence (HTRF) ratio = (channel 1/channel 2) * 10,000; % activation = (HTRF – BKGD/Max) * 100. Percent activation of compound titration was used to find activation EC_50_, where fit = (A + {(B − A)/1 + [(C/x)^D^]}) and A = bottom; B = top; C = relative EC_50_; D = Hill slope where bottom = 0; and top = 100 of control compound CMPD001. One hundred % activation signal = pUb activated Parkin plus 40 μM control activator CMPD001; 0% activation signal = pUb activated Parkin + DMSO, Parkin activator compounds can be identified by an increase in activation signal from the 0% activation signal TR-FRET.

### Probe assay

Probe binding to Parkin protein was determined by incubation of 40 nM heat-activated Parkin, 70 nM Ha-Ub-VS probe (Boston Biochem U-212), and 2x activator/2% DMSO (Sigma-Aldrich, D4540-100ML) for 60 min at 22°C with reaction buffer [50 mM Hepes (pH 8.5), 150 mM NaCl, 0.01% Tween 20, and 0.1% BSA] to total 10-μl reaction, followed by the addition of 2.6 nM anti–6His-Eu cryptate (CisBio 61HISKLA) and 40 nM anti–HA-XL665 (CisBio 610HAXLA) in detection buffer [50 mM HEPES (pH 8.5), 150 mM NaCl, 0.01% Tween 20, 0.1% BSA, 800 mM KF]. Plates were incubated for 60 min at rt, and read on a PerkinElmer Envision in white 384-well plate (Corning 3572) with top mirror: LANCE/DELFIA Duel/Bias, emission filter APC665 EM, and 2nd emission filter Europium 615 EM, reading 655 (channel 1) and 615 nm (channel 2) wavelengths on Envision, with HTRF ratio = (channel 1/channel 2) * 10,000; % activation = (HTRF – BKGD/Max) * 100. Percent activation of compound titration was used to find activation EC_50_, where fit = (A + {(B-A)/1 + [(C/x)^D]}) and A = bottom; B = top, C = relative EC_50_, D = Hill slope.

### Statistical analysis

All statistical data were calculated and graphed using GraphPad Prism version 10.2.3 (RRID:SCR_002798). To show statistical significance, we used either a one-way or two-way analysis of variance (ANOVA) test with appropriate multiple comparison tests. Statistical significance is denoted as *P < 0.05; **P < 0.01; ***P < 0.001; ****P < 0.0001; ns, not significant. Error bars are reported as means ± SD. To ensure reproducibility of experiments, we showed one representative replicate here of at least two replicates, as indicated in figure legends.

### Antibodies

The primary antibodies used in this study for Western blotting are as follows: anti-ATP5A (1:1000, Abcam, catalog no. ab176569, RRID:AB_2801536), anti-ATF3 (1:1000, Cell Signaling Technology, catalog no. 33593, RRID:AB_2799039), anti-Parkin (1:1000, Cell Signaling Technology, catalog no. 4211, RRID:AB_2159920), anti–phospho-ubiquitin S65 (1:1000, Cell Signaling Technology, catalog no. 62802, RRID:AB_2799632), anti-IFRD1 (1:1000, Novus, catalog no. NBP1-87327, RRID:AB_11052376), anti-COX4l2 (1:1000, Proteintech, catalog no. 11463-1-AP, RRID:AB_2085287), anti-FECH (1:1000, Proteintech, catalog no. 14466-1-AP, RRID:AB_2231579), anti-OPA1 (1:1000, Proteintech, catalog no.27733-1-AP, RRID:AB_ 2810292), anti-ATF4 (1:500, Santa Cruz Biotechnology, catalog no. sc-390063, RRID:AB_2058752), anti-HRI (1:1000, Proteintech, catalog no. 20499-1-AP; RRID:AB_10697665), anti-IREB2 (1:1000, Cell Signaling Technology, catalog no.37135; RRID:AB_2799110), and anti-PINK1 (1:2000, Cell Signaling Technology, catalog no.6946; RRID: AB_11179069).

The secondary antibodies used in this study for Western blotting are as follows: goat anti-rabbit (H + L)–HRP conjugate (Bio-Rad Laboratories, catalog no. 1706515, RRID:AB_11125142) and goat anti-mouse (H + L)–HRP conjugate (Bio-Rad Laboratories, catalog no. 1706516, RRID:AB_2921252).

### Cell lines

All parental cell lines, including HeLa (RRID:CVCL_0058), HEK293T (RRID:CVCL_0063), and SH-SY5Y (RRID:CVCL_0019) were acquired from the ATCC. YFP-Parkin/mt-Keima, YFP-Parkin/PINK1KO/mt-Keima, and FRB-Fis1/mt-Keima HeLa cells were acquired from the Youle laboratory. KI DELE1-HA 293 T line was obtained from the L. Jae laboratory. FRB-Fis1/FKBP-GFP-Parkin/mt-Keima HeLa and mt-Keima SH-SY5Y cells were generated from this study by stable lentiviral transduction.

### Use of generative AI and AI-assisted technologies

During the preparation of this work, the author(s) used GPT4-4o and GPT-4.5, Open AI to edit writing, code, and translate code/math into interpretable methods. After using this tool, the authors reviewed and edited the content as needed and takes full responsibility for the content of the published article. Examples of iterative prompt conversations used in this study are included below as permanent links:

Example 1, GPT-4o: “Creating an interactive HTML from PINK1 threshold simulation” https://chatgpt.com/share/682e1e70-f3b4-8013-9243-d10cc6a2f151;

Example 2, GPT-4o: “A pipeline to organize 384-well replicate data for viability measurements” https://chatgpt.com/share/68389f72-76d0-8013-9307-e294104f631d.

### Software and code

Software and code used in this study are listed below:

1) Prism software (version 10.2.3; GraphPad Software, Boston, MA, USA) for statistical analysis and graph generation (RRID:SCR_002798);

2) Image Lab software (version 6.1; Bio-Rad Laboratories, Hercules, CA, USA) for densitometry analysis of Western blots (RRID:SCR_014210);

3) ImageJ software (Schindelin et al. 2015) (version 1.54j) for confocal microscopy image processing (RRID:SCR_003070);

4) MetaXpress software (version 6.7.1.157; Molecular Devices, San Jose, CA, USA) for analysis of mt-Keima imaging (RRID:SCR_016654);

5) CytExpert software (version 2.6; Beckman Coulter, Brea, CA, USA) for live analysis of flow cytometry data (RRID:SCR_017217);

6) FlowJo software (version 10.10.0; BD Biosciences, Ashland, OR, USA) for analysis of flow cytometry data (RRID:SCR_008520); and

7) Custom Python scripts.

## Supplementary Materials

### The PDF file includes:

Supplementary Methods Tables S1 to S4 Figs. S1 to S10 Uncropped blots and full blots for each antibody Legends for tables S5 and S6 Legend for code S1

### Other Supplementary Material for this manuscript includes the following:

Tables S5 and S6

Code S1
