# Supplementary material for: Putative PINK1/Parkin activators lower the threshold for mitophagy by sensitizing cells to mitochondrial stress
Source: Sci Adv. 2025 Aug 27;11(35):eady0240. doi: 10.1126/sciadv.ady0240 (PMC12383277; doi:10.1126/sciadv.ady0240)
Supplement: Supplementary file 1 — Supplementary Methods Tables S1 to S4 Figs. S1 to S10 Uncropped blots and full blots for each antibody Legends for tables S5 and S6 Legend for code S1 [file sciadv.ady0240_sm.pdf]

Science Advances
AAAS

Supplementary Materials for

Putative PINK1/Parkin activators lower the threshold for mitophagy by sensitizing cells to mitochondrial stress

William M. Rosencrans *et al.*

Corresponding author: William M. Rosencrans, wrosencr@caltech.edu; David C. Chan, dchan@caltech.edu; Tsui-Fen Chou, tfchou@caltech.edu

Sci. Adv. **11**, eady0240 (2025)
DOI: 10.1126/sciadv.ady0240

The PDF file includes:

Supplementary Methods
Tables S1 to S4
Figs. S1 to S10
Uncropped blots and full blots for each antibody
Legends for tables S5 and S6
Legend for code S1

Other Supplementary Material for this manuscript includes the following:

Tables S5 and S6
Code S1

Supplementary Text
Chemistry.

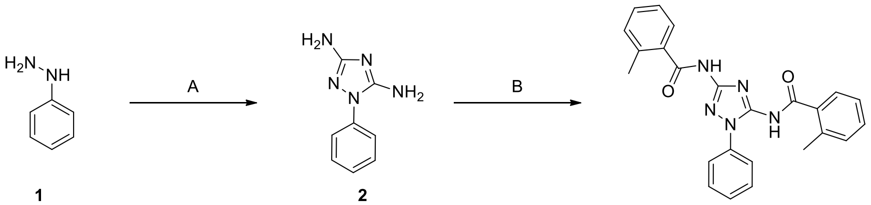

1 2 FB231

Reagents and conditions: (A) dicyandiamide, 2M *aq.* HCl, reflux, 16 h; B) 2-methylbenzoyl chloride, pyridine, MeCN, 80 °C, 20 min.

Chemistry. Mass spectra were acquired on LC-MS systems using electrospray, chemical and electron impact ionization methods from a range of instruments of the following configurations: SHIMADZU LCMS-2020, Agilent 1200 LC/G1956A MSD and Agilent 1200\G6110A, Agilent 1200 LC & Agilent 6110 MSD Mass Spectrometer $[M+H]^+$ refers to protonated molecular ion of the chemical species. NMR spectra were run on Bruker 400MHz spectrometers using ICON-NMR, under TopSpin program control. Spectra were measured at 298K, unless indicated otherwise, and were referenced relative to the solvent resonance.

Prepared HPLC condition:

column: Phenomenex luna C18 250 × 50 mm × 10 µm; mobile phase: [water (0.05%HCl)-ACN].

Instrumentation:

LC-MS Methods: Using SHIMADZU LCMS-2020, Agilent 1200 LC/G1956A MSD and Agilent 1200\G6110A, Agilent 1200 LC & Agilent 6110 MSD

Method 1: 0-60CD_2MIN_220&254

Column	XBridge C18 2.1×50mm, 5μm
Column Temperature	40 °C
Eluents	A: 0.025% $NH_3 \cdot H_2O$ in water（v/v）, B: Acetonitrile (v/v)
Flow Rate	1 ml/min
Gradient	1.2 min, 0% to 60% B; 0.4 min, 60% B, 60% to 0% B in 0.01 min, 0.39 min 0% B
Ionization source	ESI

Drying Gas	N_2
Drying Gas Flow	10(L/min)
Nebulizer Pressure	35 (psig)
Drying Gas Temp	350(°C)
Capillary Voltage	2500(V)
MS Polarity	Positive
MS Mode	Scan
Mass range	100-1000

1-phenyl-1*H*-1,2,4-triazole-3,5-diamine (2)

The mixture of phenylhydrazine (128.6 g, 1.19 mol, 1 eq) and 1-cyanoguanidine (100 g, 1.19 mol, 1 eq) in hydrochloric acid (500 mL) (2 M) at reflux for 16 h. After being cooled to room temperature, the mixture was basified with aq. NaOH (40 wt%) to pH=8. The solvent was concentrated under reduced pressure. The residue was triturated with hexane (800 mL) for1 h. The crude product was collected by filtration which was further triturated with DCM (1 L) for 16 h. The crude product was collected by filtration which was further triturated with DCM/MeOH (1 L/50 mL) for 16 h. The solid was collected by filtration and dried *in vacuo* to afford 1-phenyl-1*H*-1,2,4-triazole-3,5-diamine (140 g, crude) as a brown solid.

LC-MS (ESI): m/z (M+H) 176.0

***N,N'*-(1-phenyl-1*H*-1,2,4-triazole-3,5-diyl)bis(2-methylbenzamide) (FB231)**

To a solution of 1-phenyl-1*H*-1,2,4-triazole-3,5-diamine (60 g crude, 342 mmol, 1 eq) and pyridine (235 g, 2.97 mol, 240 mL, 8.68 eq) in ACN (400 mL) was added 2-methylbenzoyl chloride (225 g crude, 1.46 mol, 189 mL, 4.25 eq) at 80 °C. The mixture was stirred at 80 °C for 20 min. After being cooled to 15 °C, LiOH (62.8 g, 2.62 mol, 7.66 eq) in 100 mL H_2O was added to and the mixture was stirred at 15 °C for 16 h. The mixture was concentrated under reduced pressure. The mixture was extracted with CH_2Cl_2 (500 mL) and washed with H_2O (300 mL). The organic layer was separated and concentrated under reduced pressure. The residue was purified by reverse phase column (ACN/0.1% FA as additive) to give 24 g of crude product, which was purified by silica gel chromatography (petroleum ether / ethyl acetate =5/1 to 1/1) to afford *N,N'*-(1-phenyl-1*H*-1,2,4-triazole-3,5-diyl)bis(2-methylbenzamide) (20 g TFA salt, 93%purity) as an off-white solid. This TFA salt was dissolved in EtOAc (200 mL) and the solution was washed with aqueous $NaHCO_3$ (20%, 200 mL). The organic layer was separated and concentrated under reduced pressure to give 18 g freebase as a white solid. The residue (that was combined with 27 g of freebase, EW4062-260-P1) was dissolved in MeOH (150 mL) and hydrochloric acid (2 M, 150 mL). The solution was concentrated under under vacuum to give 46.8 g of *N,N'*-(1-phenyl-1*H*-1,2,4-triazole-3,5-diyl)bis(2-methylbenzamide) HCl salt (99% purity) as a yellow solid. This batch of HCl salt was

combined with 10.2 g of EW4062-262-P1 with 98% purity to give a single batch, which was registered as AH00231.01.06.
LC-MS (ESI): m/z (M+H) 412.2; ^{1}H NMR (400MHz, DMSO-*d6*) δ= 11.25- 11.02 (m, 1H), 10.92 (s, 1H), 7.63-7.61 (m, 2H), 7.58-7.55 (m, 2H), 7.52-7.45 (m, 3H), 7.43-7.39 (m, 2H), 7.33-7.27 (m, 4H), 2.43 (s, 3H), 2.22 (s, 3H); ^{13}C NMR (75 MHz, DMSO-*d6*) δ= 168.5, 167.6, 154.5, 145.3, 136.8, 136.2, 135.6, 134.1, 130.9, 130.6, 129.3, 128.5, 127.4, 125.7, 125.6, 123.5, 19.4, 19.2.

One-pot TPP experiment
The one-pot TPP protocol was adopted from literature with minor adjustment.(69,70) The cells are lysed by freeze-thaw in phosphate-buffered saline (PBS) using liquid nitrogen for 5 cycles. Cell debris was removed by centrifugation at 20000g for 15min. Protein concentration was determined using Bradford assay, and was adjusted to ~5 mg/ml. The lysate was subsequentially aliquoted into 12 aliquots, and each aliquot was incubated with MTK or FB231 under different concentration for 1 hr under room temperature. The 12 samples were grouped into 3 sample groups, with 3 aliquots treated with each of the following drug concentration: 100 uM MTK, 40 uM FB231. The rest 6 samples were treated with only DMSO, and the DMSO concentration in each sample was adjusted to ~0.5% (v/v). Each of the 12 aliquots was further aliquoted into 12 aliquots, and each of the small aliquots was heated under each of the following temperatures (43.0C, 43.5C, 44.7C, 46.6C, 49.2C, 51.8C, 54.2C, 56.8C, 59.4C, 61.3C, 62.5C, and 63.0C). The aliquots were heated under corresponding temperature for 3 min, followed by 3 min equilibration under room temperature, before sitting back on ice. 12 aliquots for the same sample were pooled together, and the precipitation is removed by ultracentrifugation under 100000g for 20 min. The supernatant is collected and subjected to an isobaric-labeled filter-aided sample preparation (iFASP) protocol with minor changes.(McDowell et al., 2013) Briefly, supernatant from each sample was loaded onto a 10kDa Amicon filter (Pierce), and washed with 8M Urea in 100mM HEPES (Urea buffer) for 3 times. Without further notice, each washing step includes adding 200 ul of the corresponding solution followed by 14000 g centrifugation for 15 min. After 3 washes with Urea buffer, 200 ul of Urea buffer containing 5 mM tris(2-carboxyethyl)phosphine (TCEP) was added into each filter to break disulfide bonds. The reaction was allowed for 1hr under room temperature, and 200 ul of Urea buffer containing 20 mM of chloroacetamide (CAA) was added into each filter to alkylate free thiols. The alkylation reaction was allowed for 15 min under room temperature, and the filters were centrifuged for 14000 g for 15 min. The filters were further washed by 150 ul of 100 mM of triethylamine bicarbonate (TEAB) in water for 3 times. After TEAB washes, 120 ul of 100 mM TEAB containing 1 ug of Trypsin (Pierce) was added into each filter. Enzyme to substrate ratio should be around 1:50 to 1:100. The trypsinization step was allowing under 37C for 16 hr. After trypsinization, 41 ul of acetonitrile containing 0.5 mg of TMTpro reagent (Thermo) was added into each filter, and the labeling was allowed for 1 hr. 4 ul of 5%

hydroxylamine was added into each filter to quench the TMT labeling, and samples were eluted from the filters by 14000g centrifugation for 15 min. The filters were further washed by 50 ul of 0.5 M NaCl in water for 3 times, and all elutes were pooled together. The pooled sample was dried using a CentriVap concentrator (LabConco), and was desalted with a monospin C18 column (GL Science) according to manufacturer's instruction. The desalted sample was dried again using a CentriVap concentrator, and was further fractionated into 8 fractions using the Pierce High pH Reversed-Phase Peptide Fractionation Kit (Pierce) according to manufacturer's protocol. The fractionated samples were dried again using CentriVap concentrator (LabConco) and stored under -80C until LC-MS/MS analysis.

LC-MS/MS analysis for TMT labeled samples

The samples were reconstituted in 20 ul of 2% acetonitrile and 0.2% formic acid in water. Peptide concentration was determined using the Pierce Colorimetric Quantitative Peptide Assay, and an aliquot of 500 ug of the peptide was used for LC-MS/MS analysis. The sample is separated on an Aurora UHPLC Column (25 cm × 75 µm, 1.6 µm C18, AUR2-25075C18A, Ion Opticks) using an Thermo Vanquish Neo liquid chromatography system. The gradient settings follows **Table S1**.

Time	Duration	Flow (nl/min)	%B
0:00	0:00	300	2
7:30	7:30	300	6
90:00	72:00	300	25
120:00	30:00	300	40
121:00	1:00	300	98
130:00	9:00	300	98

Table S1. LC gradient for TMT samples

Mobile Phase A contains 0.2% formic acid, 2% acetonitrile, and 97.8% water, and mobile Phase B contains 0.2% formic acid, 80% acetonitrile, and 19.8% water.

The samples were analyzed on a Thermo Eclipse Tribrid mass spectrometer using a data-dependent acquisition method. Detailed parameters of the scans are listed in **Table S2**.

**Global settings**	
Ion source type	NSI
Spray voltage	1500 V
Ion transfer tube temperature	275 C
Polarity	Positive
**MS1 scan settings**	
Resolution	120000

Normalized AGC target	250
Maximum IT	50 ms
Scan range	375-1600 m/z
**MS2 scan settings**	
Resolution	50000
Normalized AGC target	Standard
Maximum IT	Dynamic
Loop time	3 sec
Isolation window	0.7 m/z
NCE	35
Spectrum data type	Centroid
Fixed first mass	110 z

Table S2. MS settings for TMT samples

Data analysis for TMT-labeled proteomic samples

The raw data generated by mass spectrometer is analyzed using Proteome Discoverer 2.5. The data is searched using the mouse proteome achieved from UniprotKB on 10/26/2022. The parameters for PD search are listed below in **Table S3**. All the parameters that are not mentioned are left defaulted.

**SequestHT settings**	
Enzyme name	Trypsin (Full)
Max. missed cleavage	2
Min. peptide length	6
Max. peptide length	144
Precursor mass tolerance	10 ppm
Fragment mass tolerance	0.02 Da
Max. equal modification	3
Dynamic modification	Oxidation/ +15.995 Da (M)
Dynamic modification (protein terminus)	Acetyl/ + 42.011 Da (N-Terminal)
Dynamic modification (protein terminus)	Met-loss/ - 131.040 Da (M)
Dynamic modification (protein terminus)	Met-loss+Acetyl/ - 89.030 Da (M)
Static modification (peptide terminus)	TMTpro/ + 304.027 Da (N-Terminal)
Static modification	TMTpro/ + 304.027 Da (N-Terminal)
Static modification	Carbamidomethyl/ + 57.021 Da (C)
**Percolator**	

Target/Decoy selection	Concatenated
Validation based on	q-Value
Target FDR (Strict)	0.01
Target FDR (Relaxed)	0.05

Table S3. Parameters for PD searching

The resulting data was exported as excel file and processed by in-house python scripts. Briefly speaking, the protein abundances were median normalized across samples, and Welch's t-test was used to test if the drug treated samples were significantly different from DMSO treated samples.

Interactive Simulation Method

Overview

This study simulates the effects of two inhibitors on mitochondrial function and subsequent mitophagy activation. The simulation aims to explore how varying concentrations of a weak inhibitor and a strong inhibitor influence mitochondrial function and mitophagy levels, represented by mathematical models implemented in Python. The simulations are visualized through dose-response curves for mitochondrial function and mitophagy across different experimental conditions.

Mathematical Modeling

Logistic Dose-Response Function

The logistic dose-response function used to model mitochondrial function is given by :

(1) $$f(dose) = bottom + \frac{1 - bottom}{1 + \left(\frac{dose}{IC50}\right)^{hill}}$$

where:

- dose is the concentration of the inhibitor.
- IC50 is the half-maximal inhibitory concentration.

- hill is the Hill coefficient that determines the steepness of the curve.
- bottom is the baseline residual response.

The parameters used for the simulations were set as follows: for the weak inhibitor, IC50 was 1.0, the Hill coefficient was 1.0, and bottom values ranged between 0.3 and 0.99, while for the strong inhibitor, IC50 was 0.25, and the Hill coefficient was 1.0 with a bottom value of 0.0.

Mitophagy Response Function

The mitophagy response to changes in mitochondrial function was modeled using a power law equation. The response was calculated as:

(2) $$R(m) = f(x) = \begin{cases} 0, \; if \; < 0 \\ \left[\frac{threshold - m}{threshold}\right]^n, \; \text{otherwise} \end{cases}$$

where:

- *m* represents mitochondrial function.
- *threshold* is the activation threshold.
- *n* is a power coefficient to determine the steepness of the response. For all simulations, the activation threshold was set to 0.3, and n was set to 1

Calculation of EC50 Values

EC50 values for mitophagy response were determined using interpolation to achieve a higher level of accuracy. The *EC50* value is defined as the dose at which the response reaches 50% of its maximum. For each dose-response curve, the dose corresponding to the response closest to 0.5 was identified, and if it was not at the boundary, linear interpolation was applied between the closest points to estimate the *EC50* value.

Simulation Parameters

The following parameters were used in the simulation:

Weak Inhibitor:

- *IC50* = 1.0
- *Hill* coefficient = 1.0
- *Baselin*e = 0.4

Strong Inhibitor:

- *IC50* = 0.25
- *Hill* coefficient = 1.0
- *Baseline* = 0.0

Mitophagy Activation:

- Threshold for activation = 0.3
- Power coefficient *n* = 1

Simulations and Visualization

Doses for the inhibitors were simulated using logarithmically spaced values ranging from 10^{-3} to 10^{3} for the strong inhibitor and 10^{-3} to 10^{2} for the weak inhibitor. The mitochondrial function responses for the weak and strong inhibitors were calculated individually as well as in combination. The combined mitochondrial function was obtained by multiplying the individual effects of the weak and strong inhibitors.

The normalized *EC50* values for the strong inhibitor in the presence of varying doses of the weak inhibitor were also determined. The normalized *EC50* was calculated by dividing the *EC50* in the combination setting by the *EC50* of the strong inhibitor alone.

Interactive Simulation Requirements

To run the interactive dose-response simulation, the following programs and libraries are

required:

- Python 3.x
- Panel library
- Plotly library
- NumPy library
- A modern web browser (e.g., Google Chrome, Firefox, Safari) to view the interactive HTML file

To generate the interactive simulation, run the provided Python script. The output will be saved as an HTML file that can be opened in a browser to explore the effects of different parameters interactively.

Plotting and Visualization Settings

All simulations and visualizations were implemented in Python using the NumPy and Matplotlib libraries. The plots were generated to show both mitochondrial function and mitophagy response under varying conditions. The dose of the strong inhibitor at which the mitophagy response reaches 50%. The search range for *d_strong* was set between 0.001 and 1000. If no root could be found within this range, the *EC50* was reported as NaN.

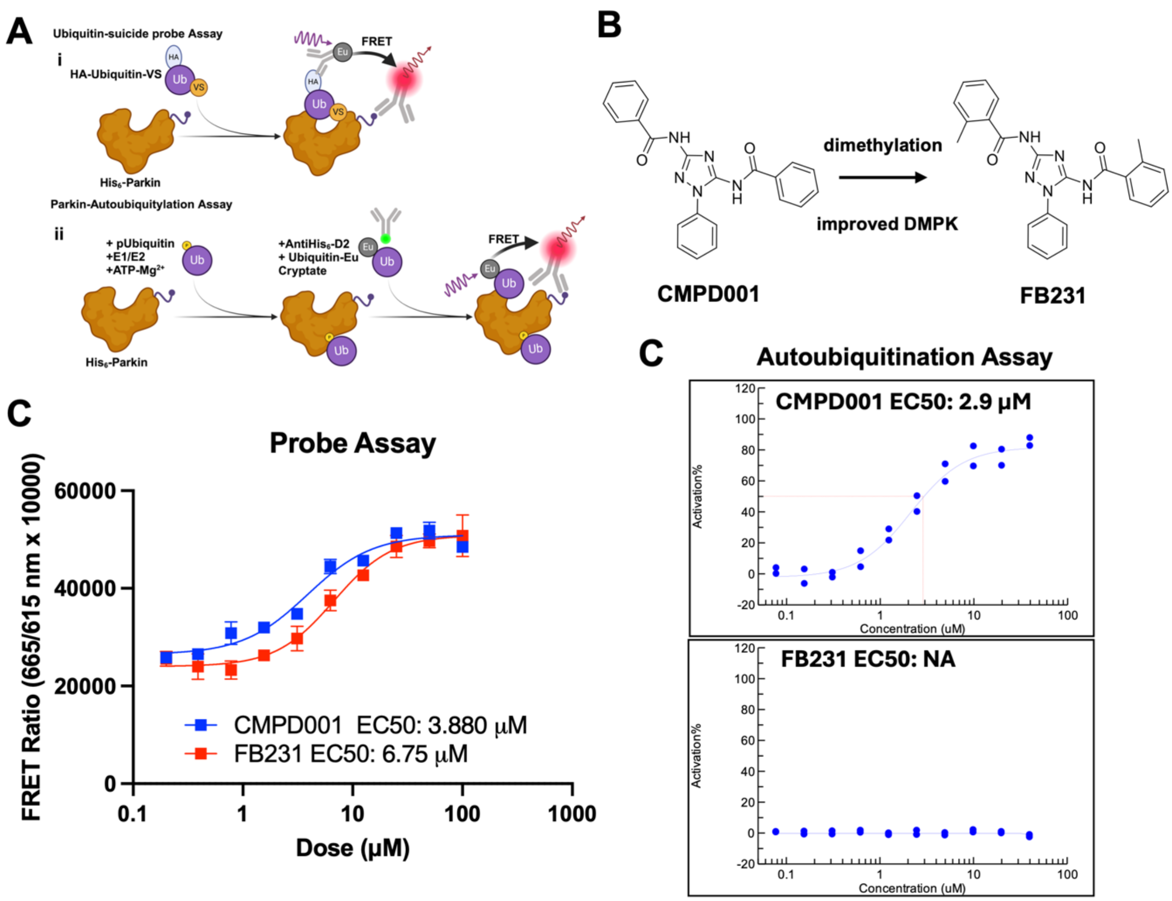

Figure S1: Identification of Parkin activating compounds through an in vitro ubiquitination assay. (A) Cartoons representing the scheme for the Ubiquitin-suicide probe assay. **(B)** Chemical structures of CMPD001 and FB231. **(C)** A representative assay readout of the Ubiquitin Probe assay for CMPD001 (Red) and FB231 (Blue), (N=2). Lines represent fits Hill Equation fits to the data to calculate the EC_{50}.

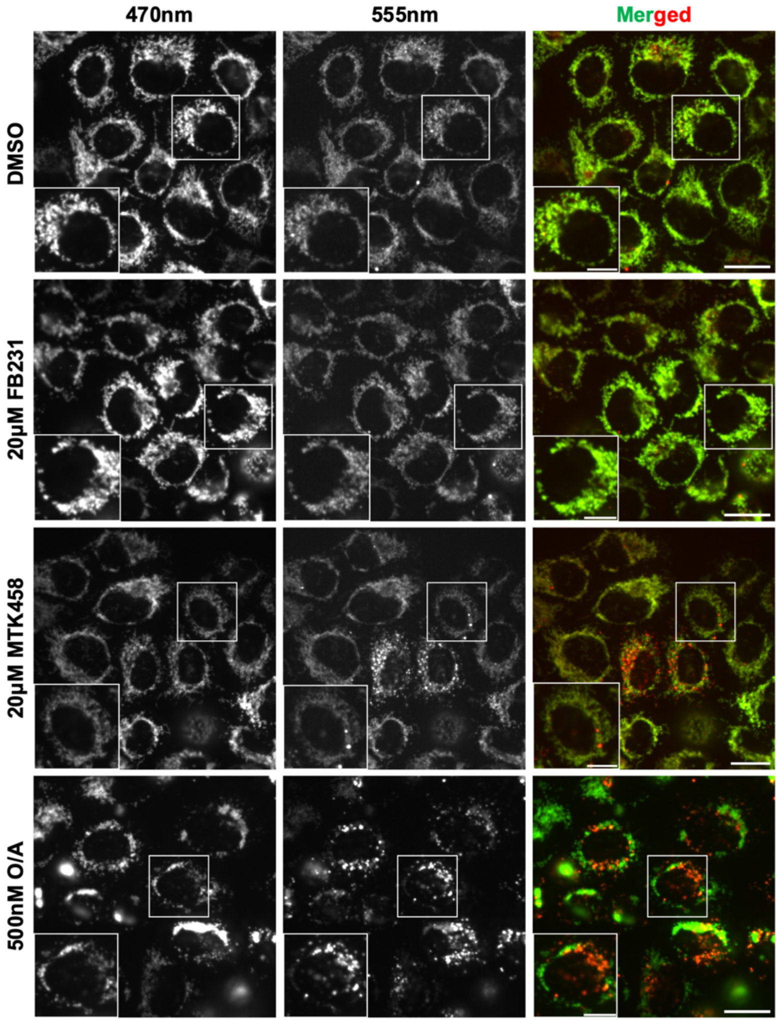

Figure S2. Mitophagy activators do not induce mitophagy without prior mitochondrial damage. Representative confocal images of YFP-Parkin/mt-Keima-expressing cells treated with FB231 or MTK458 alone. No 555 nm puncta induction are observed in the activators alone, whereas 500 nM O/A induces clear mitolysosomal puncta. Scale bars: overviews, 20 µm; insets, 10 µm.

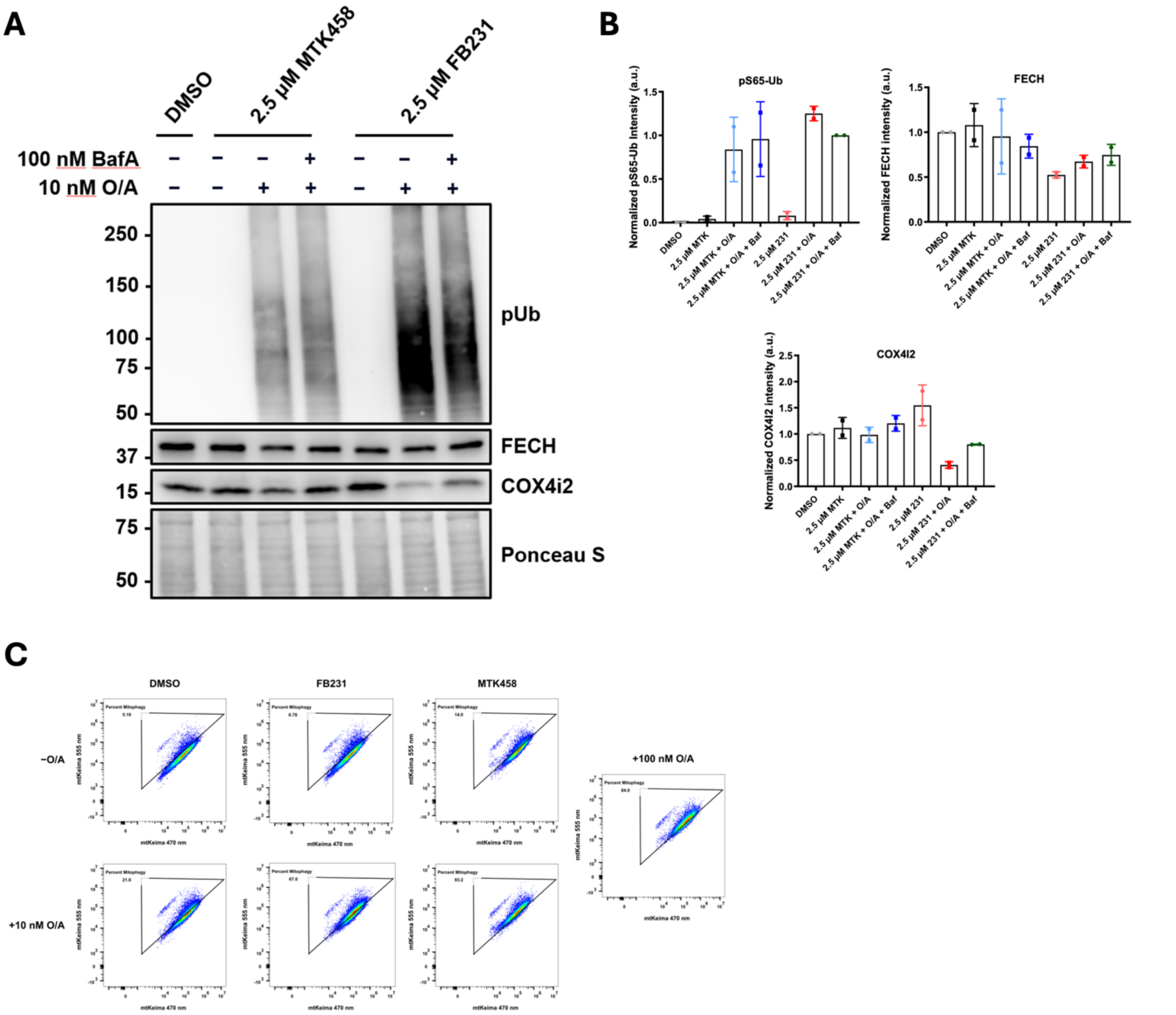

Figure S3. Additional data related to Figure 2, Parkin/PINK1 activators enable potent activation of the PINK1/Parkin pathway. (A) Immunoblots of YFP-Parkin-expressing HeLa cells treated with or without 10 nM O/A and 2.5 μM MTK458/FB231 and 100 nM BafA for 6 h. **(B)** Normalized densitometry measurements for (A), (N=2). **(C)** Representative flow cytometry plots of mt-Keima-expressing SH-SY5Y cells treated with DMSO, 10 nM O/A, 1.25 μM FB231, 2.5 μM MTK458, a combination of 10 nM O/A and FB231/MTK458 at the same doses, or 100 nM O/A for 24 h. For each sample, at least 30,000 events were collected and subsequently gated for live, single cells expressing mt-Keima. Data represents the percentage of cells undergoing mitophagy as indicated by the ratio of mt-Keima 555/470 nM emission.

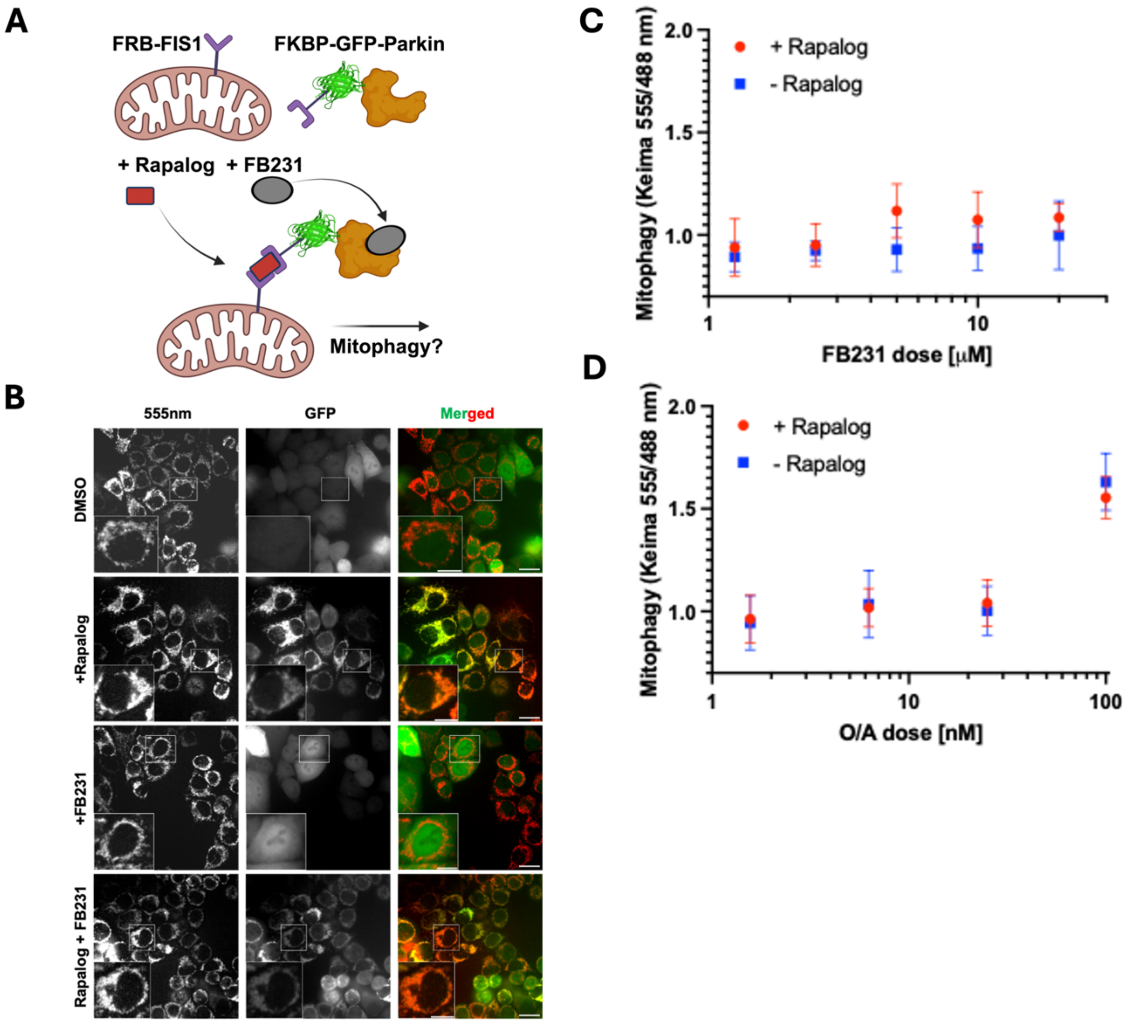

Figure S4. FB231 cannot be utilized in a Parkin-AutoTAC strategy. (A) Cartoon scheme of the CID Parkin recruitment assay to test the potential of FB231 in a Parkin-PROTAC. Parkin is tagged with FKBP-GFP while mt-Keima-expressing mitochondria are tagged with FRB-FIS1. Upon addition of a rapalog, Parkin is forced to the surface of the mitochondria. Varying doses of FB231 are added. **(B)** Representative confocal images of cytosolic FKBP-GFP-Parkin (Green) being recruited to the mitochondria (mt-Keima 555 nm) upon rapalog addition (200 nM). Scale bars: overviews, 20 µm; insets, 10 µm. **(C)** mt-Keima measurement of the Parkin recruitment assay with (red) or without (blue) rapalog upon increasing doses of FB231 after 24 h. **(D)** As in (C) with O/A.

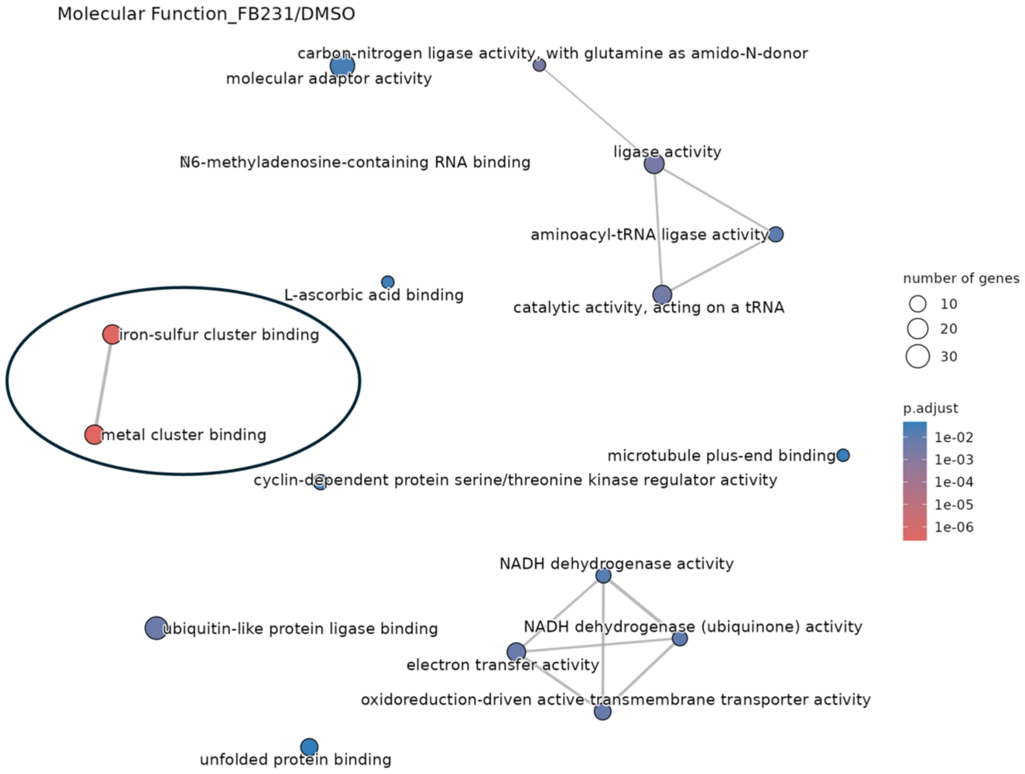

Figure S5: Proteomics identified pathways altered by FB231. GO enrichment analysis of molecular functions shows significant alterations of a cluster of proteins related to iron-sulfur/metal cluster binding in YFP-Parkin-expressing HeLa cells upon treatment with 10 μM FB231.

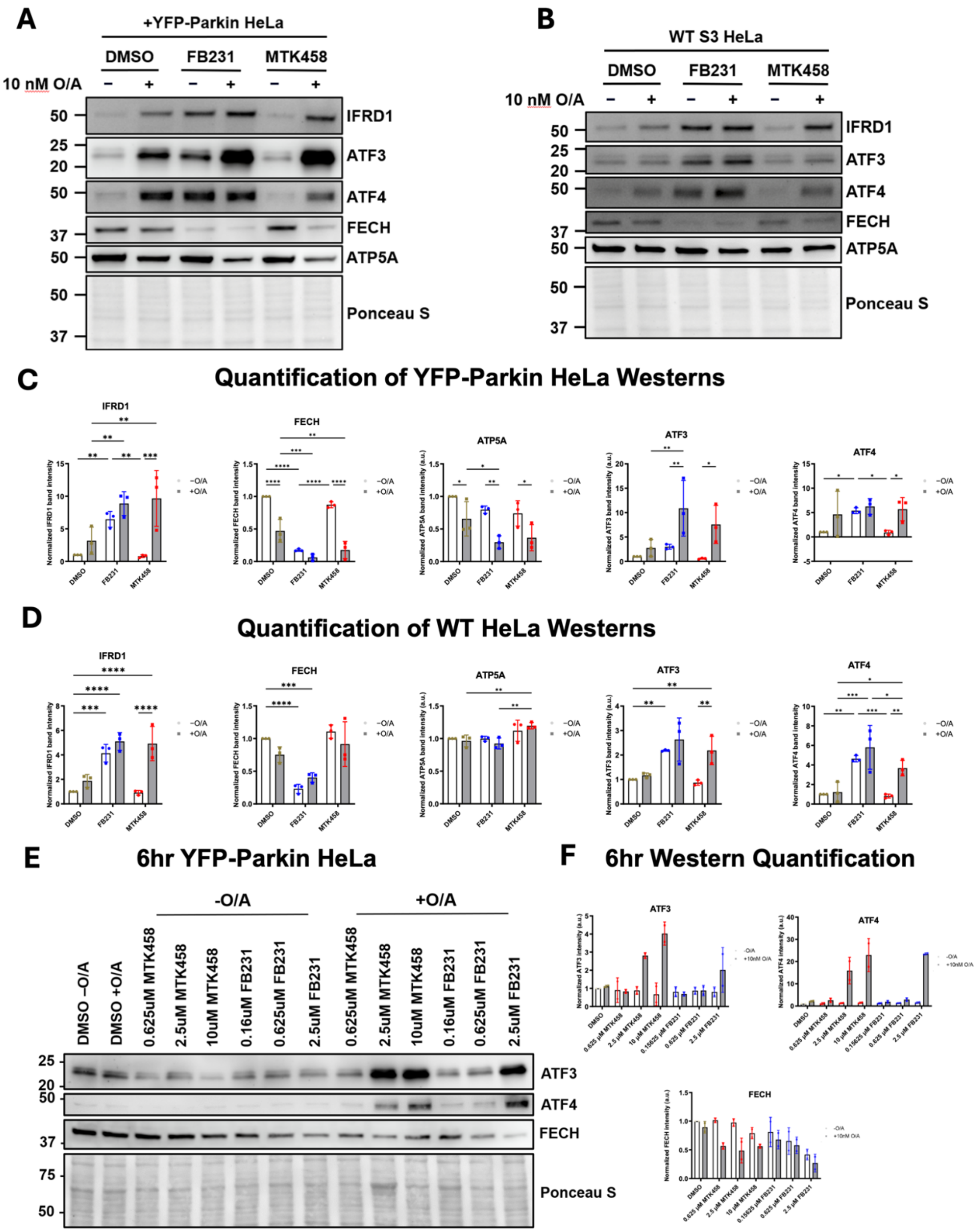

Figure S6: Additional blots related to Figure 4, Off-Targets of FB231 and MTK458. (A and **B)** Immunoblots of markers identified in the proteomic data sets. (A) YFP-Parkin-expressing and (B) WT HeLa cells were treated with DMSO, 10 nM O/A and/or 10 uM FB231 or 5 uM MTK458 for 16 h. **(C** and **D)** Normalized densitometry analysis for immunoblots in (A) and (B), respectively (N=3). **(E)** Immunoblots of ATF3, ATF4, and FECH in YFP-Parkin-expressing HeLa cells treated with various doses of MTK458/FB231 for 6 h with or without 10 nM O/A. **(F)** Normalized densitometry analysis for immunoblots in (E), (N=2). Data are presented as mean ± SD; $^{*}P \leq 0.05$, $^{**}P \leq 0.01$, $^{***}P \leq 0.001$, $^{****}P \leq 0.0001$ (Two-way ANOVA).

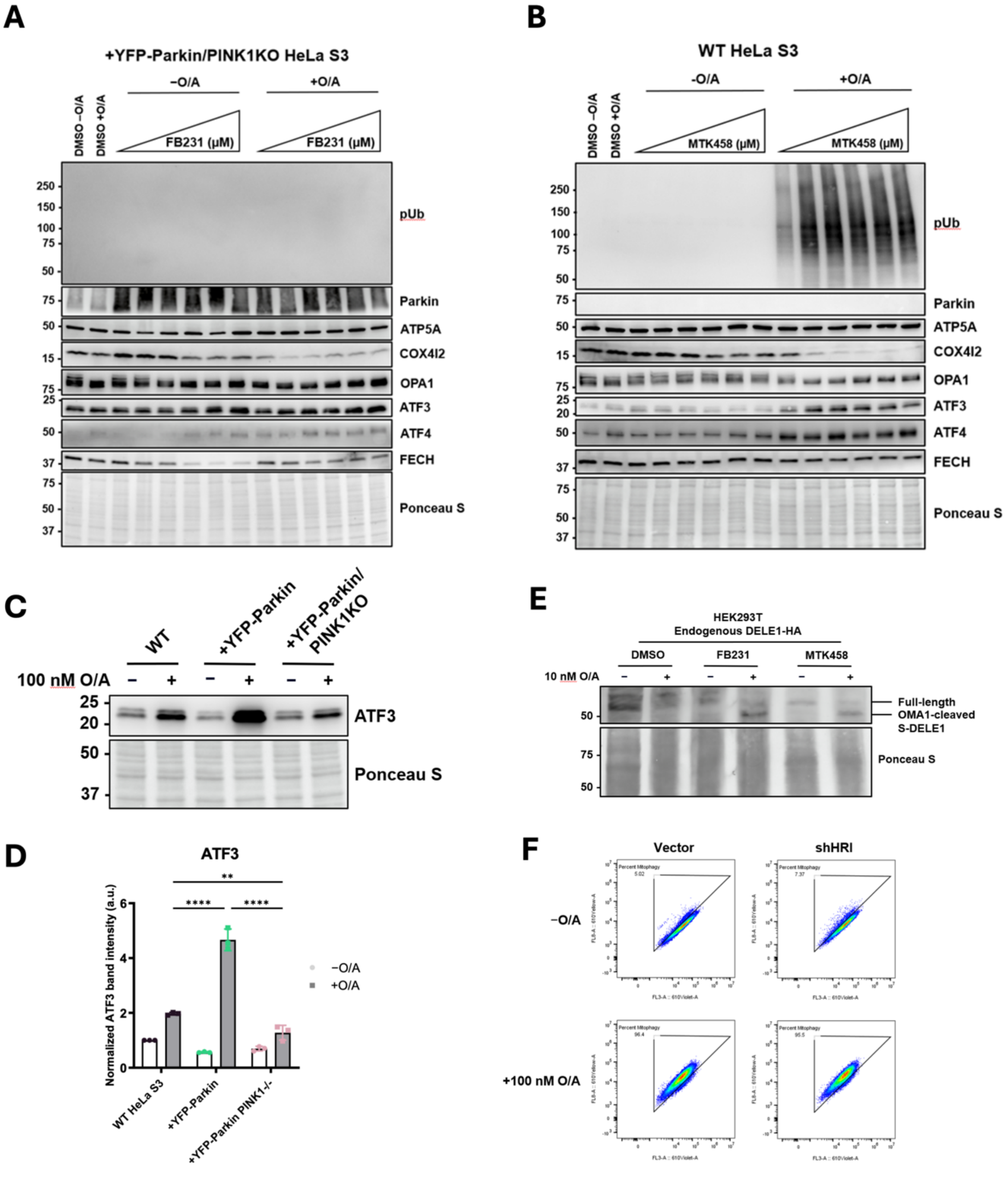

Figure S7: Additional blots related to Figure 4, FB231 and MTK458 induce Mitochondrial Stress upstream of PINK1/Parkin activation. (A) Immunoblots of mitophagy, mitochondrial stress, and integrated stress response biomarkers in YFP-Parkin/PINK1 KO HeLa cells treated with DMSO, 10 nM O/A, 0.3–10µM FB231, and the same doses of FB231 with 10 nM O/A for 16 h. **(B)** As in (A) with 0.6–20µM MTK458. **(C)** Immunoblot of ATF3, an integrated stress response biomarker, in WT, YFP-Parkin-expressing, and YFP-Parkin/PINK1KO HeLa cells treated with DMSO or 100 nM O/A. **(D)** Normalized densitometry analysis of (C), (N=3). **(E)** Immunoblots of DELE1-HA in endogenous DELE1-HA knock-in HEK293T cells treated with or without 10 nM O/A for 16 h alone or in combination with 10 µM FB231 or 10 µM MTK458. **(F)**

Representative flow cytometry plots of mt-Keima-expressing HeLa cells treated with DMSO or 100 nM O/A for 6 h. For each sample, at least 30,000 events were collected and subsequently gated for live, single cells expressing mt-Keima. Data represents the percentage of cells undergoing mitophagy as indicated by the ratio of mt-Keima 555/470 nM emission. All cells, except those in (C), were administered with 20 μM Q-VD-OPh to prevent cell death. Ponceau S stain was used as total protein loading control.

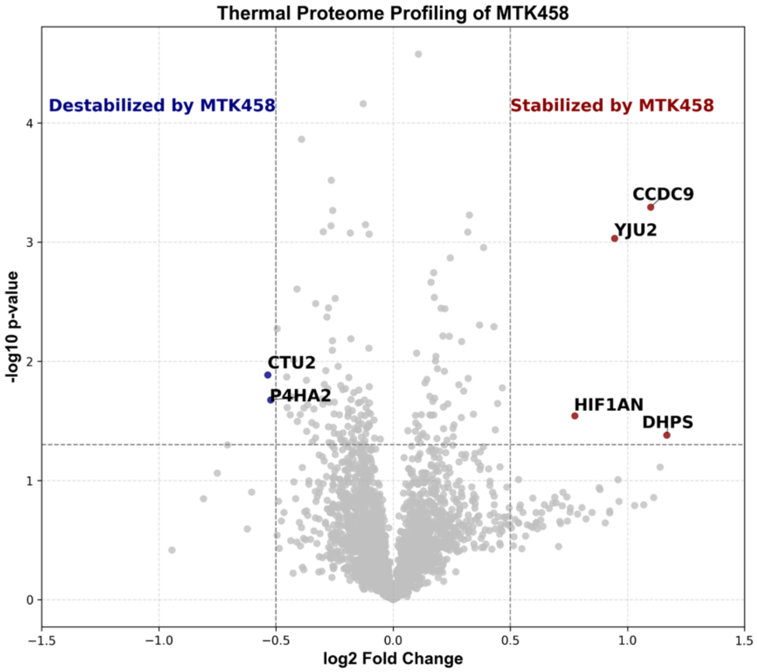

Figure S8: Thermal Proteome Profiling of MTK458.

Volcano plot of thermal proteome profiling of YFP-Parkin-expressing HeLa lysate treated with 50 μM MTK458 compared to equivalent DMSO-treated lysate. Significantly destabilized proteins compared to DMSO, with fold-change < -1 and P < 0.05 indicated in blue. Proteins significantly stabilized compared to DMSO, fold-change > 1 and P < 0.05 indicated in red. Gene names of significantly altered proteins are labelled.

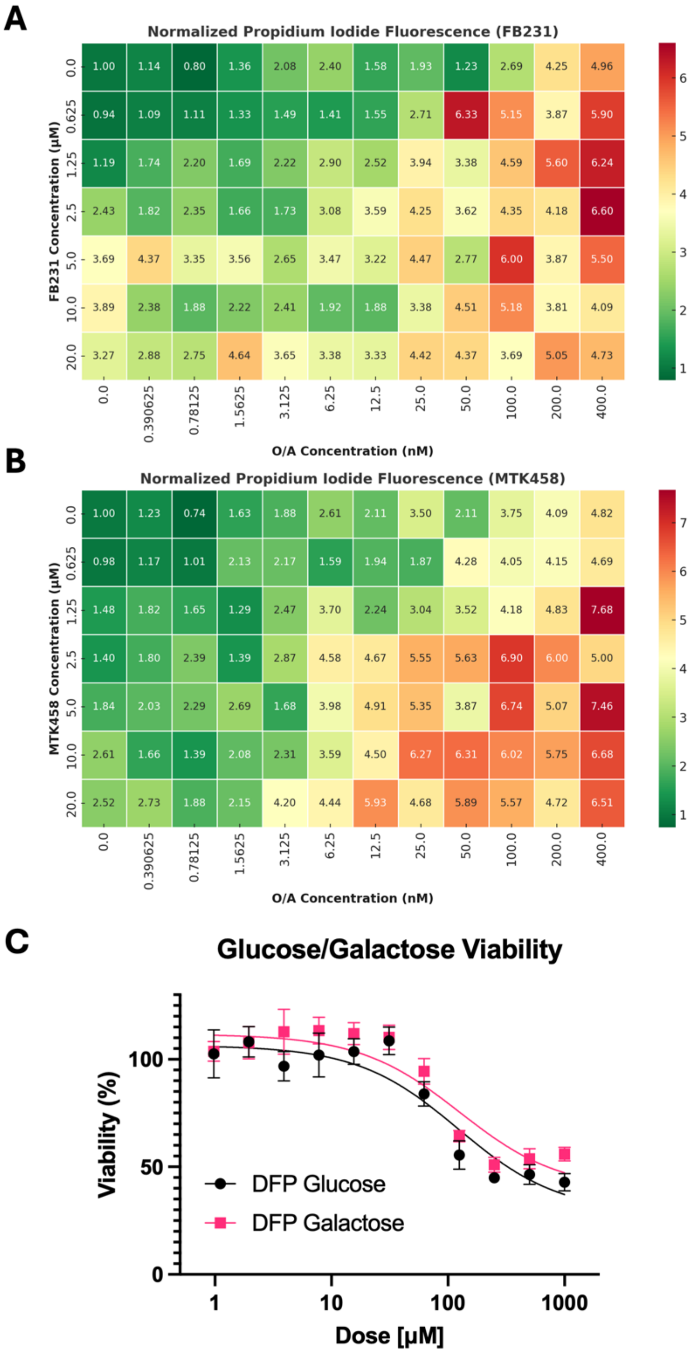

Figure S9: Additional data related to Figure 8, FB231 and MTK458 sensitize cells to mitochondrial stress

(A) Heatmap showing normalized propidium iodide (PI) fluorescence intensity, a marker of dead cells, for cells treated for 24 h with various dose combinations of FB231 and O/A. Each block represents the average intensity of N=4 wells. Fluorescence is normalized to the average of the DMSO-only condition. **(B)** as in (A) for various doses combinations of MTK458 and O/A. **(C)** Dose-viability plot of WT HeLa cells in glucose-containing media (black) or galactose-containing media (pink) treated with increasing doses of 24 h (N=8). Data are presented as means ± SD.

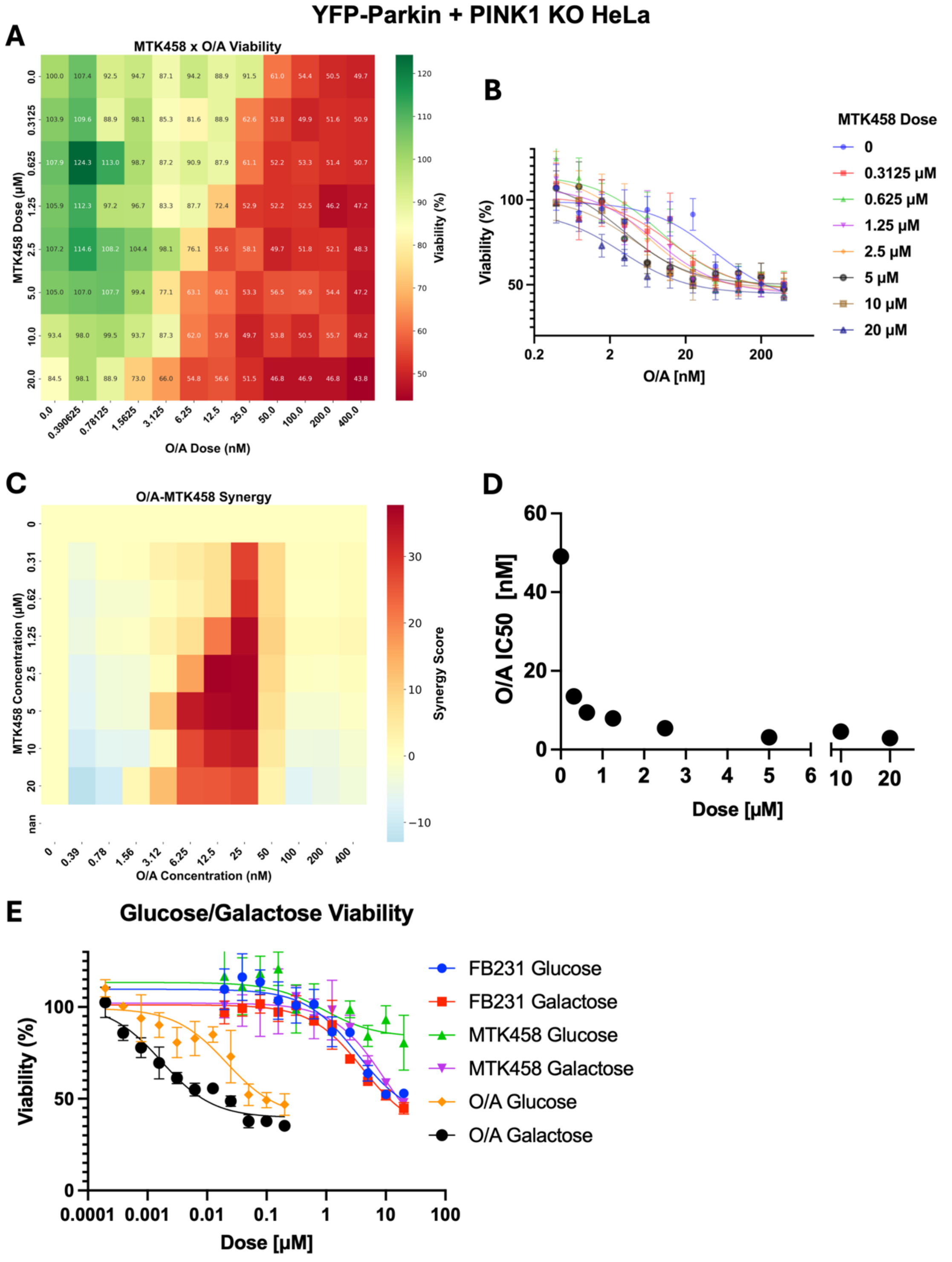

Figure S10: PINK1 is not required for MTK458-induced sensitization to mitochondrial stress.

(A) Heatmap showing cell viability as in YFP-Parkin/PINK1 KO HeLa cells treated with combination doses of MTK458 and O/A for 24 h. **(B)** YFP-Parkin/PINK1 KO HeLa cells viability

dose response curves for O/A at increasing doses of MTK458. Points represent the mean and standard deviation of 8 replicates. Solid lines are fits to a Hill equation to determine the IC_{50}. **(C)** Synergy score heat map of O/A and MTK458 combinations on cell viability. **(D)** Plot of calculated O/A viability IC_{50} at different doses of MTK458 in YFP-Parkin/PINK1 KO HeLa cells. **(E)** Dose-viability plot of YFP-Parkin/PINK1 KO HeLa cells in glucose media or galactose-containing media treated with increasing doses of O/A, FB231, or MTK458 for 24 h. Points and error bars represent the mean and standard deviation from 8 replicates. Solid lines represent fits the Hill equation.

Table S4. In vitro and in vivo comparison of CMPD001 properties and DMPK.

<table>
<tr><td rowspan="3"></td><td colspan="5">In Vitro DMPK</td></tr>
<tr><td></td><td></td><td></td><td></td><td></td></tr>
<tr><td>MDCK P_{app} (10^{-6} cm/s) A to B</td><td>Kin. Sol.
(µM)</td><td>HLM
t1/2 (min)</td><td>RLM
t1/2 (min)</td><td>cLogP</td></tr>
<tr><td>CMPD001</td><td>20</td><td>157</td><td>151</td><td>21</td><td>3.7</td></tr>
<tr><td>FB231</td><td>16</td><td>166</td><td>578</td><td>630</td><td>4.4</td></tr>
</table>

<table>
<tr><td rowspan="3"></td><td colspan="8">In Vivo DMPK - Rat</td></tr>
<tr><td colspan="2">IV (1 mg/kg)</td><td colspan="2">PO (5 mg/kg)</td><td colspan="4">IP 3 mg/kg)</td></tr>
<tr><td>Cl (mL/min/kg)</td><td>Vss (L/kg)</td><td>%F</td><td>AUC (0-t) (ng.hr/mL)</td><td>AUC (0-t) (ng.hr/mL)</td><td>Cmax (ng/mL)</td><td>t1/2 (hr)</td><td>%F</td></tr>
<tr><td>CMPD001</td><td>15.7</td><td>0.4</td><td>7%</td><td>0.4K</td><td></td><td></td><td></td><td></td></tr>
<tr><td>FB231</td><td>4</td><td>0.4</td><td>24%</td><td>5.2K</td><td>10K</td><td>1.5K</td><td>3.2h</td><td>75%</td></tr>
</table>

Figure 2E: Activator titration mitophagy markers +YFP-Parkin HeLa S3 – Repl. 1 (representative)

***Repl. = Replicate**

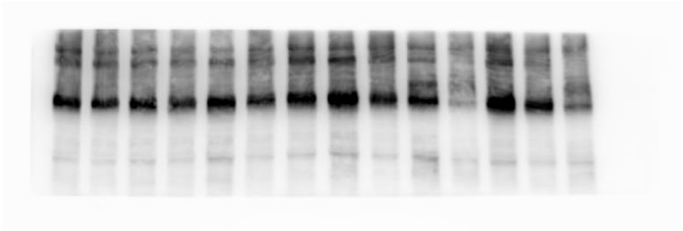

Parkin

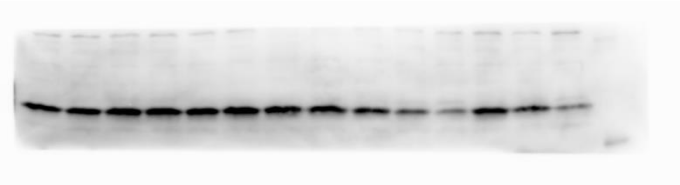

COX4l2

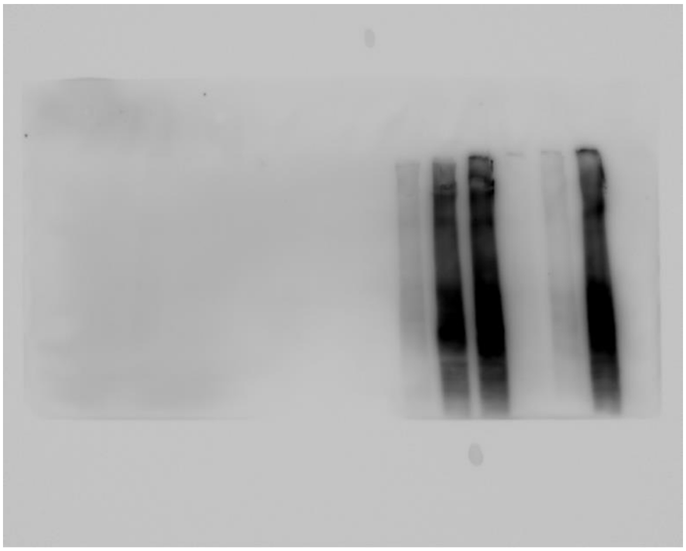

pUb

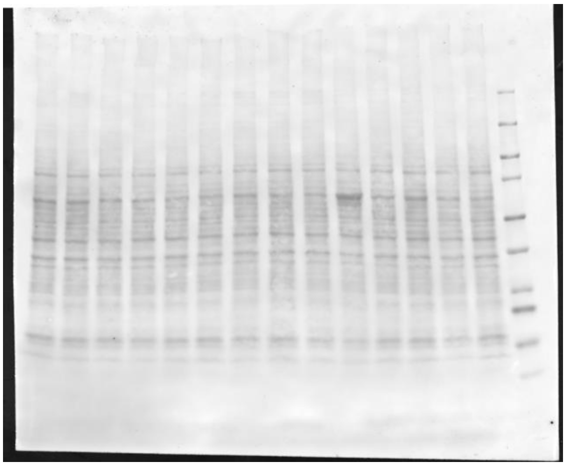

Ponceau S

Gel Loading Order (same for all blots):

1. DMSO
2. +10 nM O/A
3. 0.6 µM MTK458
4. 2.5 µM MTK458
5. 10 µM MTK458
6. 0.16 µM FB231
7. 0.6 µM FB231
8. 2.5 µM FB231
9. 0.6 µM MTK458 + 10 nM O/A
10. 2.5 µM MTK458 + 10 nM O/A
11. 10 µM MTK458 + 10 nM O/A
12. 0.16 µM FB231 + 10 nM O/A
13. 0.6 µM FB231 + 10 nM O/A
14. 2.5 µM FB231 + 10 nM O/A
15. Ladder

Figure 2E: Activator titration mitophagy markers +YFP-Parkin HeLa S3 – Repl. 2

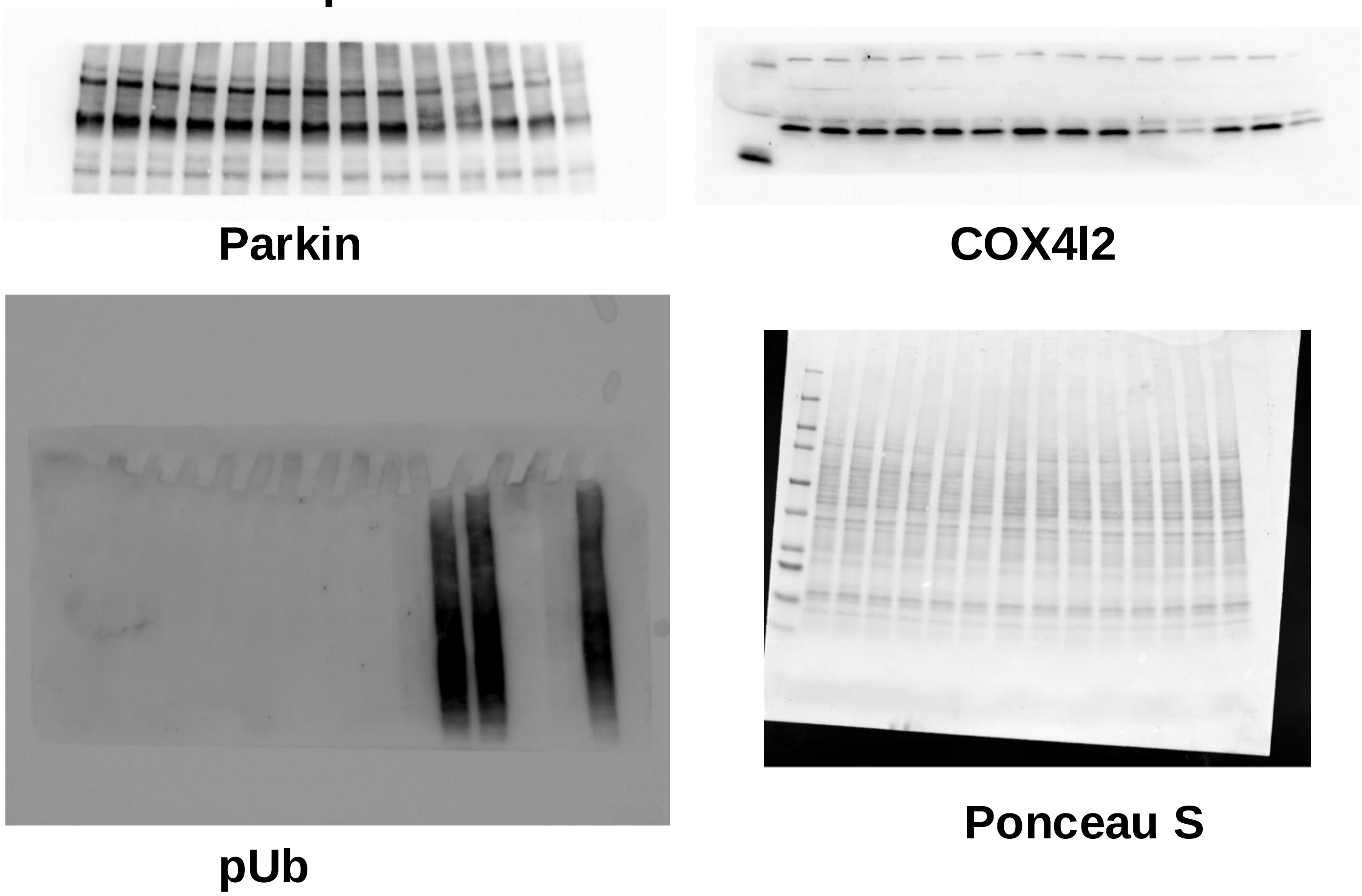

Gel Loading Order (same for all blots):

1. Ladder
2. DMSO
3. +10 nM O/A
4. 0.6 µM MTK458
5. 2.5 µM MTK458
6. 10 µM MTK458
7. 0.16 µM FB231
8. 0.6 µM FB231
9. 2.5 µM FB231
10. 0.6 µM MTK458 + 10 nM O/A
11. 2.5 µM MTK458 + 10 nM O/A
12. 10 µM MTK458 + 10 nM O/A
13. 0.16 µM FB231 + 10 nM O/A
14. 0.6 µM FB231 + 10 nM O/A
15. 2.5 µM FB231 + 10 nM O/A

Figure 3C: Activators synergy pUb +YFP-Parkin HeLa S3 – Repl. 1-3

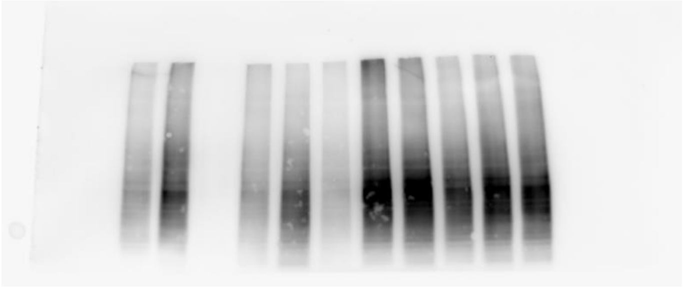

pUb 1

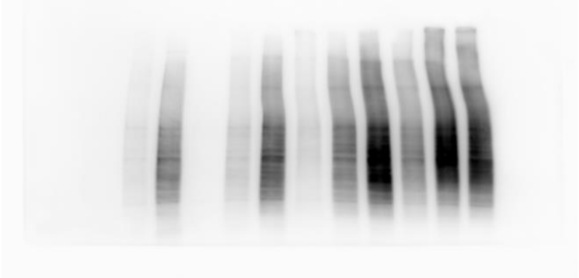

pUb 2 (representative)

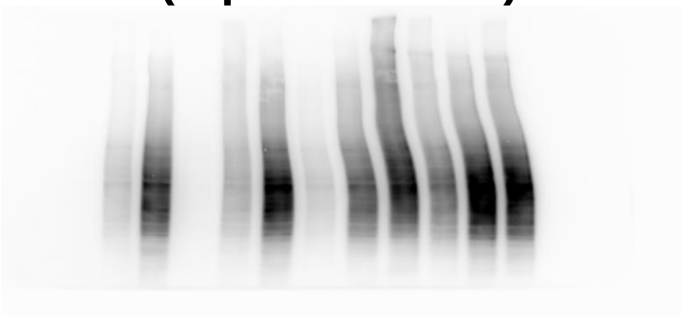

pUb 3

Gel Loading Order (same for all blots):

1. Ladder
2. DMSO
3. 1.25 μM MTK458
4. 2.5 μM MTK458
5. 0.3 μM FB231
6. 0.3 μM FB231 + 1.25 μM MTK458
7. 0.3 μM FB231 + 2.5 μM MTK458
8. 0.6 μM FB231
9. 0.6 μM FB231 + 1.25 μM MTK458
10. 0.6 μM FB231 + 2.5 μM MTK458
11. 1.25 μM FB231
12. 1.25 μM FB231 + 1.25 μM MTK458
13. 1.25 μM FB231 + 2.5 μM MTK458
14. Ladder 15. Ladder/Blank

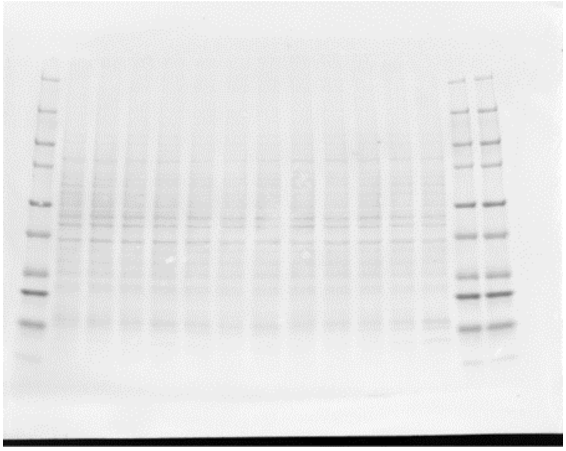

Ponceau S 1

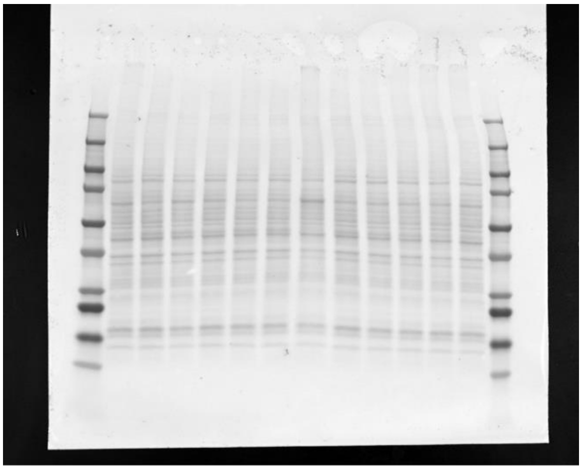

Ponceau S 2 (representative)

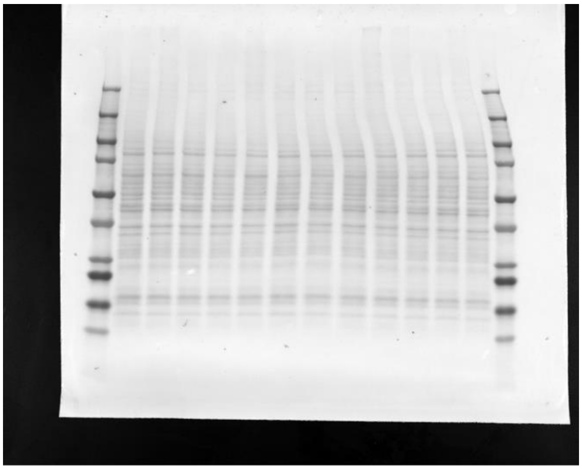

Ponceau S 3

Fig 4D: Compound dose escalation mitophagy and stress – FB231 Repl. 1 (representative) +YFP-Parkin HeLa S3

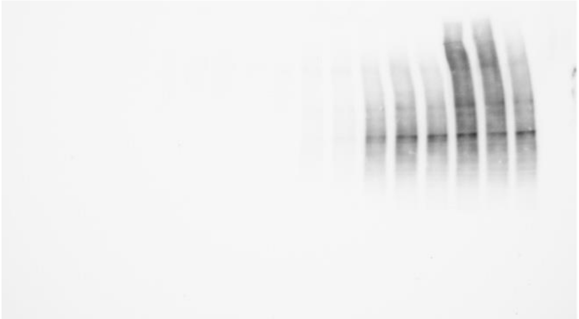

pUb

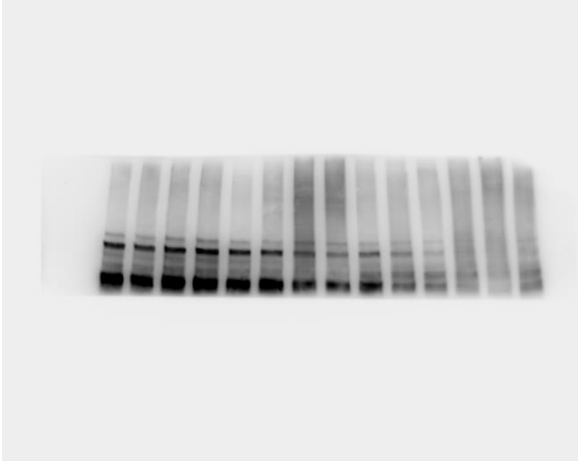

PARKIN

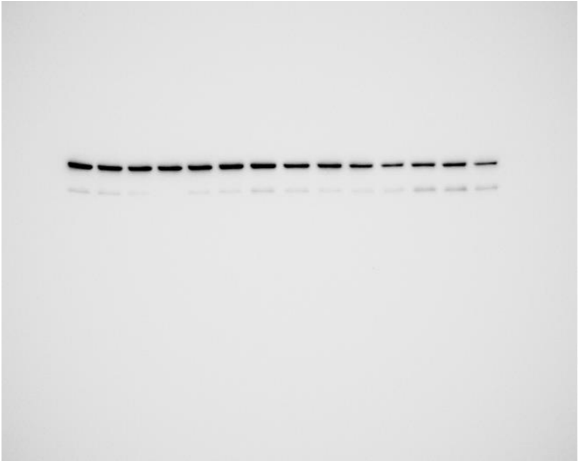

ATP5A

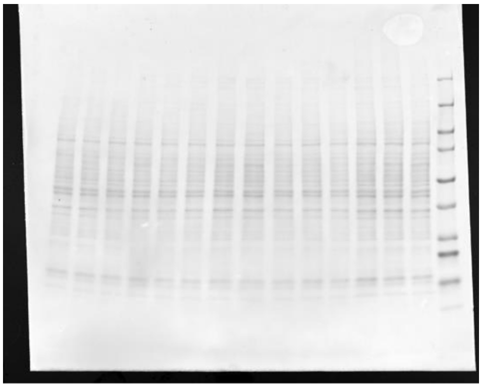

Ponceau S

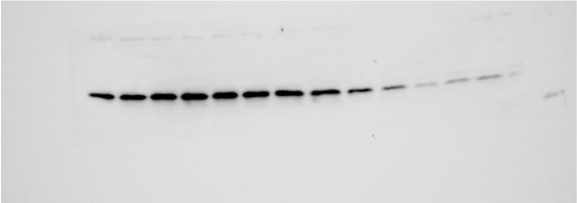

COX4I2

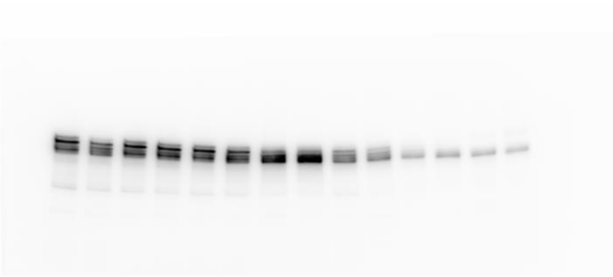

OPA1

Gel Loading Order (same for all blots):

1. DMSO
2. +10 nM O/A
3. 0.3 μM FB231
4. 0.6 μM FB231
5. 1.25 μM FB231
6. 2.5 μM FB231
7. 5 μM FB231
8. 10 μM FB231
9. 0.3 μM FB231 + 10 nM O/A
10. 0.6 μM FB231 + 10 nM O/A
11. 1.25 μM FB231 + 10 nM O/A
12. 2.5 μM FB231 + 10 nM O/A
13. 5 μM FB231 + 10 nM O/A
14. 10 μM FB231 + 10 nM O/A
15. Ladder

Figure 4D: Compound dose escalation (continued) – FB231 +YFP-Parkin HeLa S3 Repl. 1 (representative)

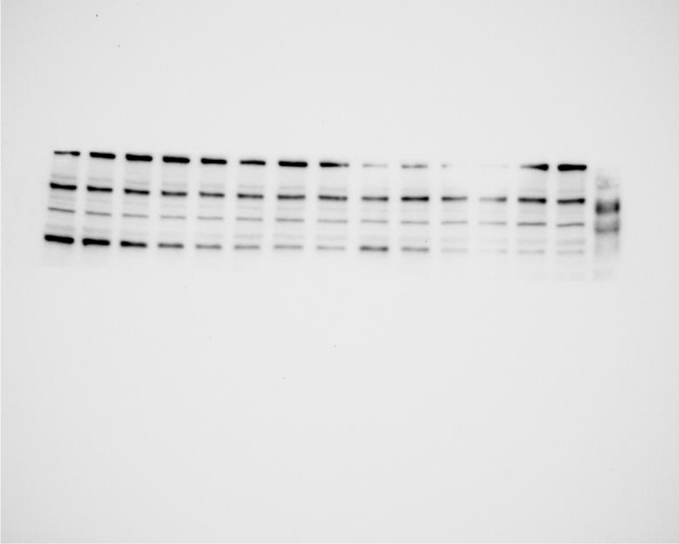

FECH

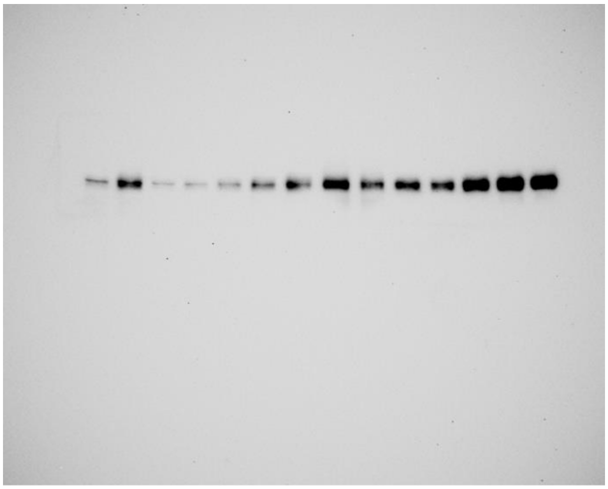

ATF4

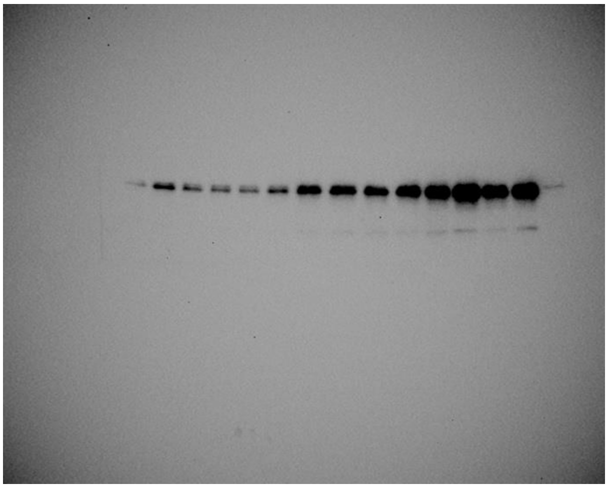

ATF3

Gel Loading Order (same for all blots):

1. Ladder
2. DMSO
3. +10 nM O/A
4. 0.3 µM FB231
5. 0.6 µM FB231
6. 1.25 µM FB231
7. 2.5 µM FB231
8. 5 µM FB231
9. 10 µM FB231
10. 0.3 µM FB231 + 10 nM O/A
11. 0.6 µM FB231 + 10 nM O/A
12. 1.25 µM FB231 + 10 nM O/A
13. 2.5 µM FB231 + 10 nM O/A
14. 5 µM FB231 + 10 nM O/A
15. 10 µM FB231 + 10 nM O/A

Fig 4D: Compound dose escalation mitophagy and stress – FB231 Repl. 2

+YFP-Parkin HeLa S3

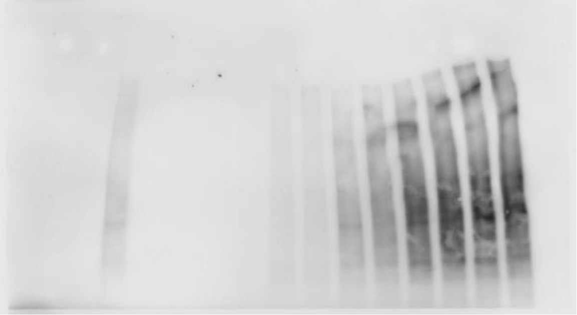

pUb

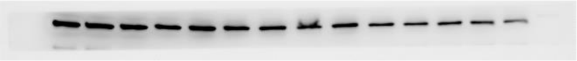

ATP5A

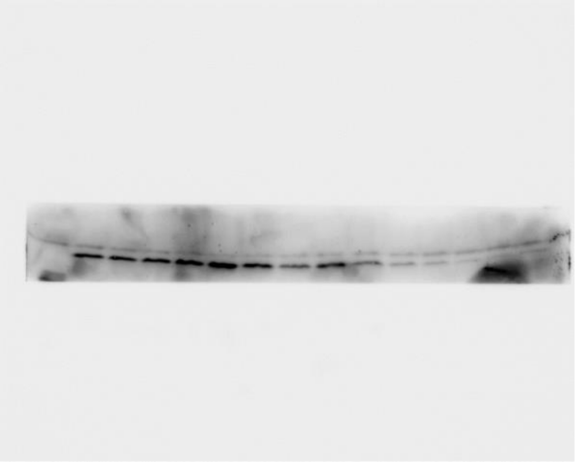

COX4I2

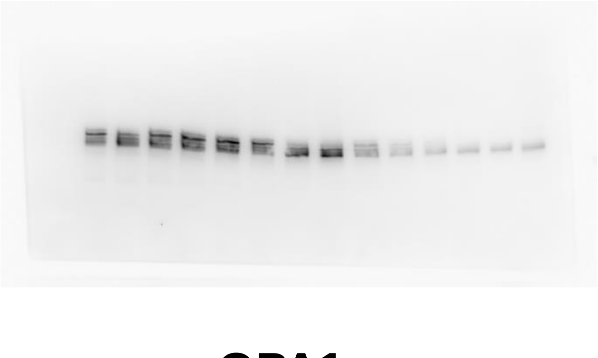

OPA1

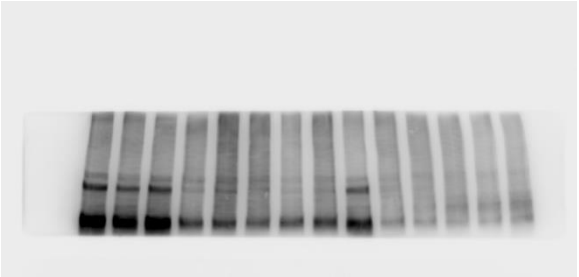

PARKIN

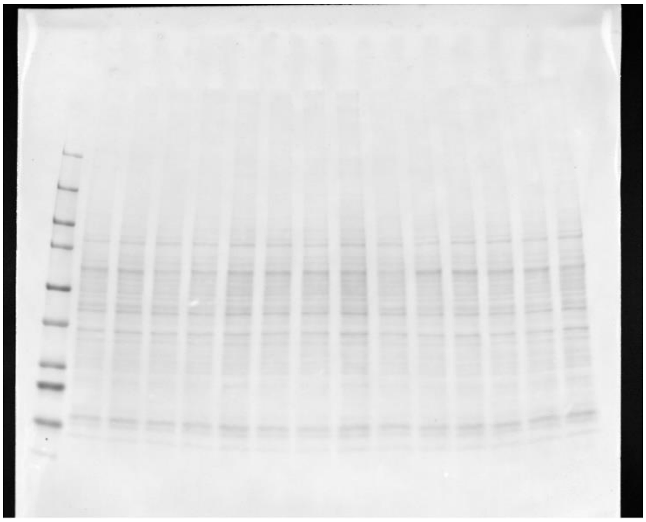

Ponceau S

Gel Loading Order (same for all blots):

1. Ladder
2. DMSO
3. +10 nM O/A
4. 0.3 μM FB231
5. 0.6 μM FB231
6. 1.25 μM FB231
7. 2.5 μM FB231
8. 5 μM FB231
9. 10 μM FB231
10. 0.3 μM FB231 + 10 nM O/A
11. 0.6 μM FB231 + 10 nM O/A
12. 1.25 μM FB231 + 10 nM O/A
13. 2.5 μM FB231 + 10 nM O/A
14. 5 μM FB231 + 10 nM O/A
15. 10 μM FB231 + 10 nM O/A

Figure 4D-E: Compound dose escalation (continued) – FB231

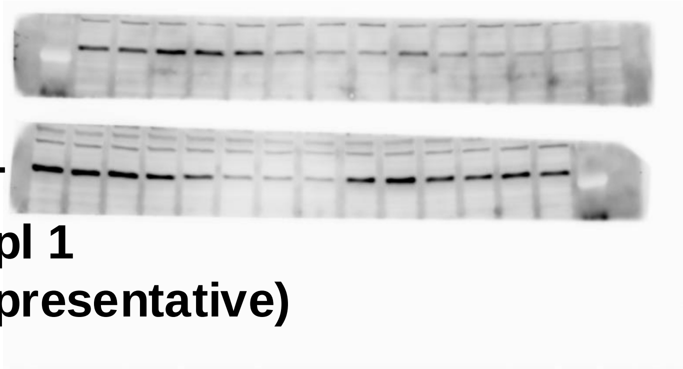

FECH

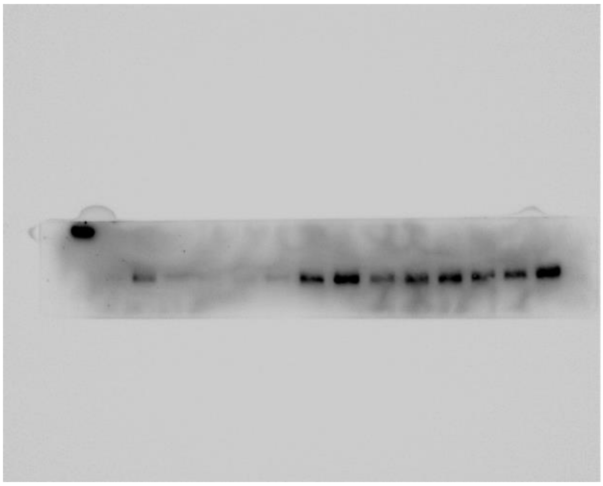

ATF4 (+YFP-Parkin)

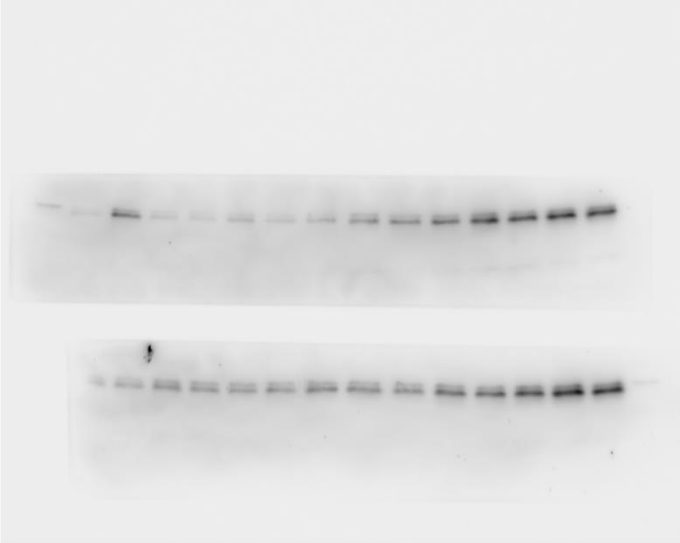

ATF3

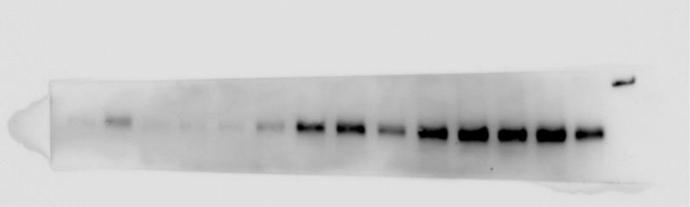

ATF4 (WT)

Gel Loading Order (same for all blots):

1. Ladder
2. DMSO
3. +10 nM O/A
4. 0.3 µM FB231
5. 0.6 µM FB231
6. 1.25 µM FB231
7. 2.5 µM FB231
8. 5 µM FB231
9. 10 µM FB231
10. 0.3 µM FB231 + 10 nM O/A
11. 0.6 µM FB231 + 10 nM O/A
12. 1.25 µM FB231 + 10 nM O/A
13. 2.5 µM FB231 + 10 nM O/A
14. 5 µM FB231 + 10 nM O/A
15. 10 µM FB231 + 10 nM O/A

Fig 4E: Compound dose escalation mitophagy and stress – FB231 Repl. 1 (representative)

WT HeLa S3

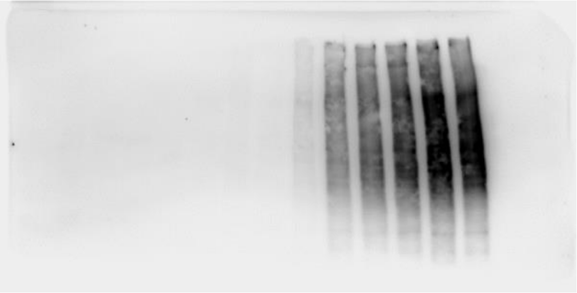

pUb

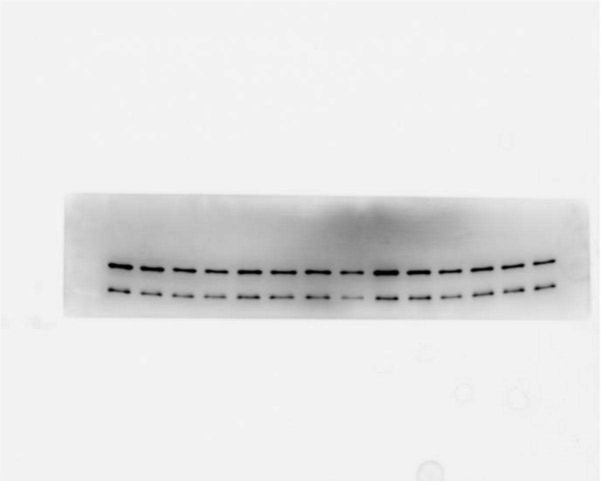

ATP5A

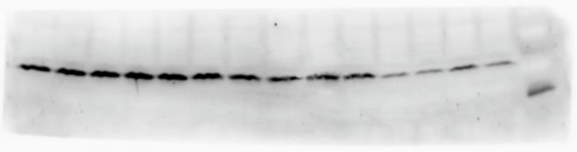

COX4I2

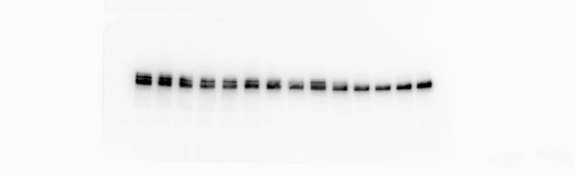

OPA1

PARKIN

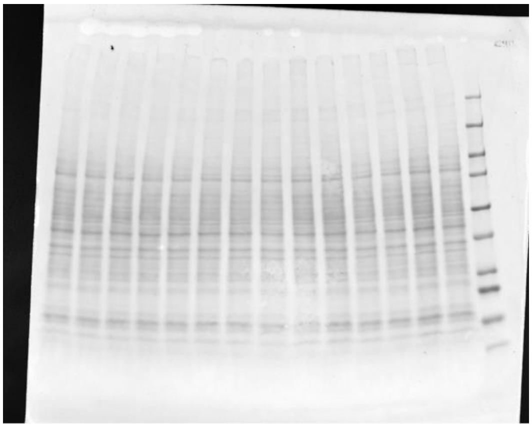

Ponceau S

Gel Loading Order (same for all blots):

1. DMSO
2. +10 nM O/A
3. 0.3 μM FB231
4. 0.6 μM FB231
5. 1.25 μM FB231
6. 2.5 μM FB231
7. 5 μM FB231
8. 10 μM FB231
9. 0.3 μM FB231 + 10 nM O/A
10. 0.6 μM FB231 + 10 nM O/A
11. 1.25 μM FB231 + 10 nM O/A
12. 2.5 μM FB231 + 10 nM O/A
13. 5 μM FB231 + 10 nM O/A
14. 10 μM FB231 + 10 nM O/A
15. Ladder

Fig 4E: Compound dose escalation mitophagy and stress – FB231 Repl. 2

WT

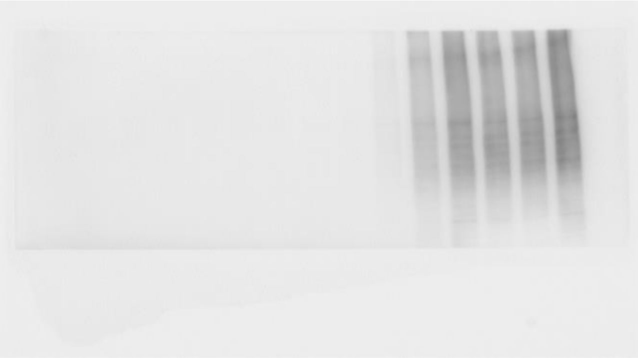

pUb

PARKIN

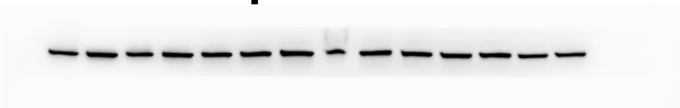

ATP5A

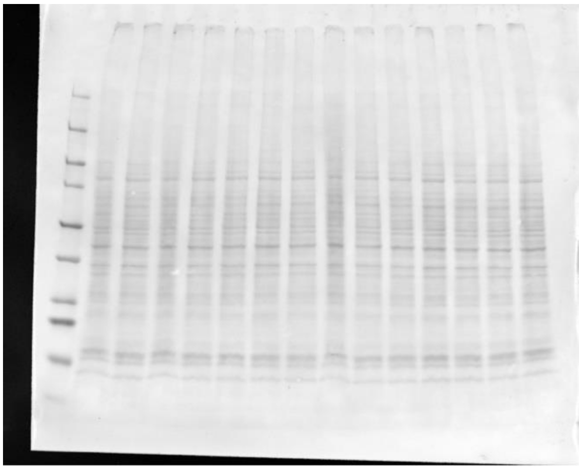

Ponceau S

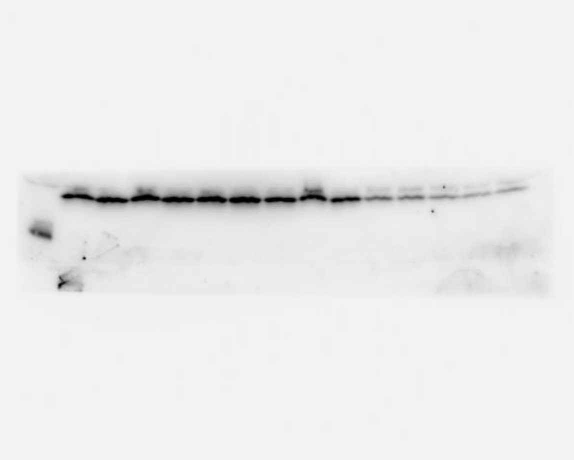

COX4I2

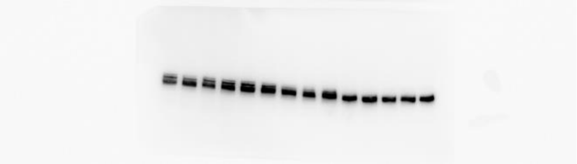

OPA1

Gel Loading Order (same for all blots):

1. Ladder
2. DMSO
3. +10 nM O/A
4. 0.3 µM FB231
5. 0.6 µM FB231
6. 1.25 µM FB231
7. 2.5 µM FB231
8. 5 µM FB231
9. 10 µM FB231
10. 0.3 µM FB231 + 10 nM O/A
11. 0.6 µM FB231 + 10 nM O/A
12. 1.25 µM FB231 + 10 nM O/A
13. 2.5 µM FB231 + 10 nM O/A
14. 5 µM FB231 + 10 nM O/A
15. 10 µM FB231 + 10 nM O/A

Figure 4E: Compound dose escalation (continued) – FB231 WT repl 2

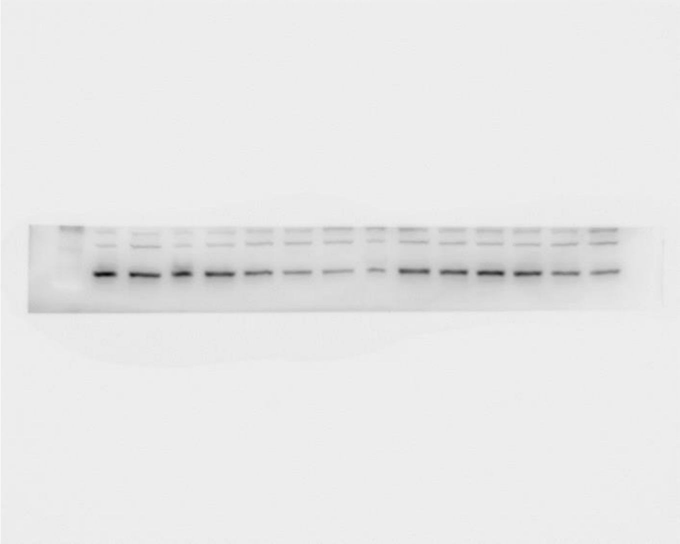

FECH

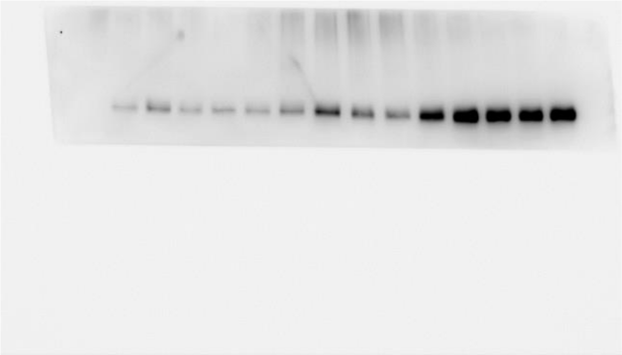

ATF4

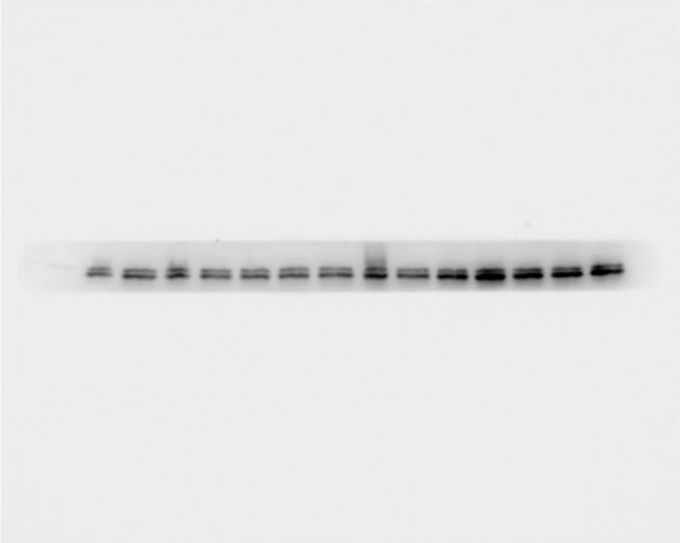

ATF3

Gel Loading Order (same for all blots):

1. Ladder
2. DMSO
3. +10 nM O/A
4. 0.3 μM FB231
5. 0.6 μM FB231
6. 1.25 μM FB231
7. 2.5 μM FB231
8. 5 μM FB231
9. 10 μM FB231
10. 0.3 μM FB231 + 10 nM O/A
11. 0.6 μM FB231 + 10 nM O/A
12. 1.25 μM FB231 + 10 nM O/A
13. 2.5 μM FB231 + 10 nM O/A
14. 5 μM FB231 + 10 nM O/A
15. 10 μM FB231 + 10 nM O/A

Fig 4D: Compound dose escalation mitophagy and stress – FB231 repl. 3
+YFP-Parkin HeLa S3

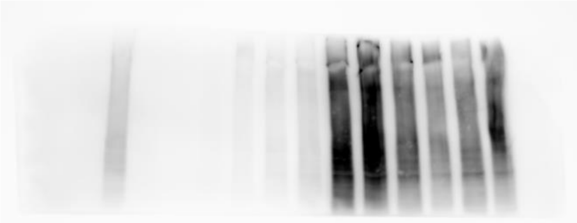

pUb

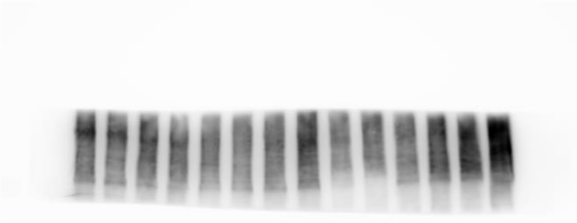

PARKIN

ATP5A

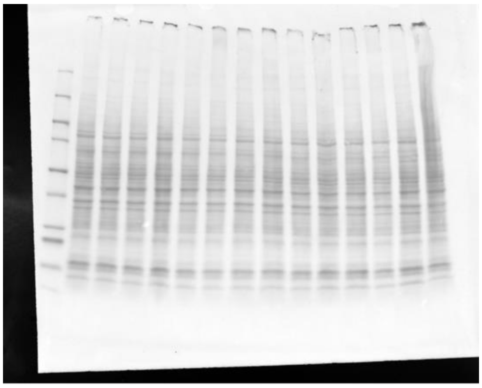

Ponceau S

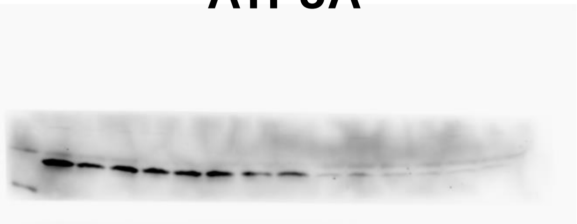

COX4I2

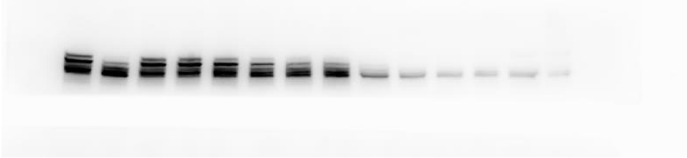

OPA1

Gel Loading Order (same for all blots):

1. Ladder
2. DMSO
3. +10 nM O/A
4. 0.3 μM FB231
5. 0.6 μM FB231
6. 1.25 μM FB231
7. 2.5 μM FB231
8. 5 μM FB231
9. 10 μM FB231
10. 0.3 μM FB231 + 10 nM O/A
11. 0.6 μM FB231 + 10 nM O/A
12. 1.25 μM FB231 + 10 nM O/A
13. 2.5 μM FB231 + 10 nM O/A
14. 5 μM FB231 + 10 nM O/A
15. 10 μM FB231 + 10 nM O/A

Figure 4D: Compound dose escalation (continued) – FB231 +YFP-Parkin HeLa S3 repl. 3

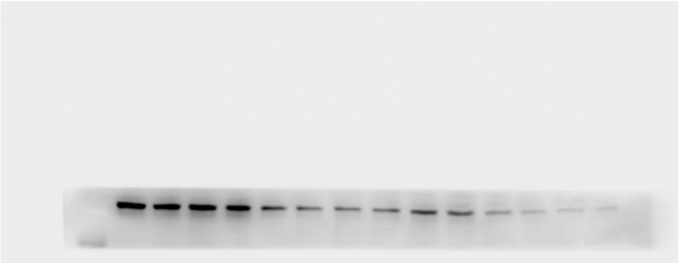

FECH

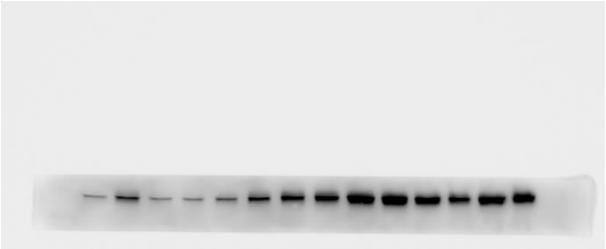

ATF4

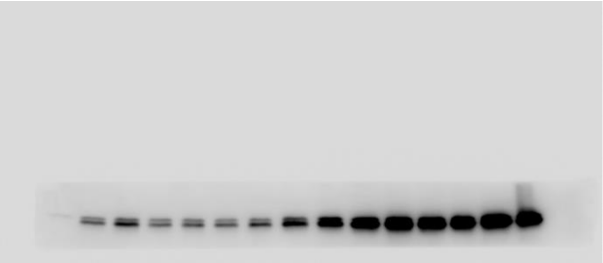

ATF3

Gel Loading Order (same for all blots):

1. Ladder
2. DMSO
3. +10 nM O/A
4. 0.3 μM FB231
5. 0.6 μM FB231
6. 1.25 μM FB231
7. 2.5 μM FB231
8. 5 μM FB231
9. 10 μM FB231
10. 0.3 μM FB231 + 10 nM O/A
11. 0.6 μM FB231 + 10 nM O/A
12. 1.25 μM FB231 + 10 nM O/A
13. 2.5 μM FB231 + 10 nM O/A
14. 5 μM FB231 + 10 nM O/A
15. 10 μM FB231 + 10 nM O/A

Fig 4E: Compound dose escalation mitophagy and stress – FB231 repl. 3
WT HeLa S3

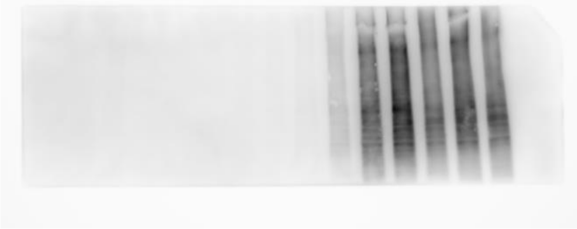

pUb

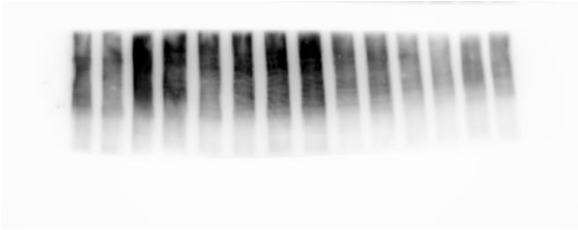

PARKIN

ATP5A

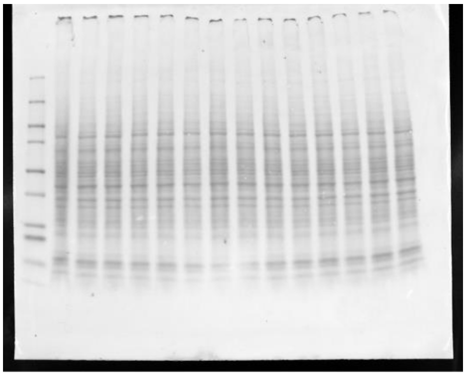

Ponceau S

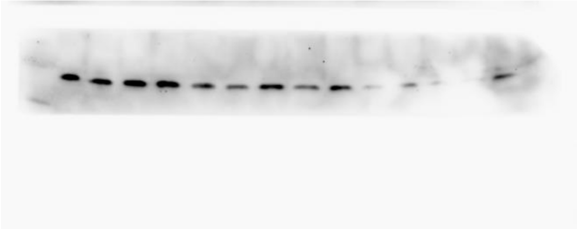

COX4I2

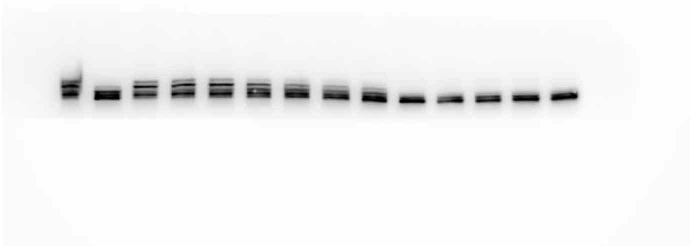

OPA1

Gel Loading Order (same for all blots):

1. Ladder
2. DMSO
3. +10 nM O/A
4. 0.3 μM FB231
5. 0.6 μM FB231
6. 1.25 μM FB231
7. 2.5 μM FB231
8. 5 μM FB231
9. 10 μM FB231
10. 0.3 μM FB231 + 10 nM O/A
11. 0.6 μM FB231 + 10 nM O/A
12. 1.25 μM FB231 + 10 nM O/A
13. 2.5 μM FB231 + 10 nM O/A
14. 5 μM FB231 + 10 nM O/A
15. 10 μM FB231 + 10 nM O/A

Figure 4E: Compound dose escalation (continued) – FB231 WT HeLa S3 repl. 3

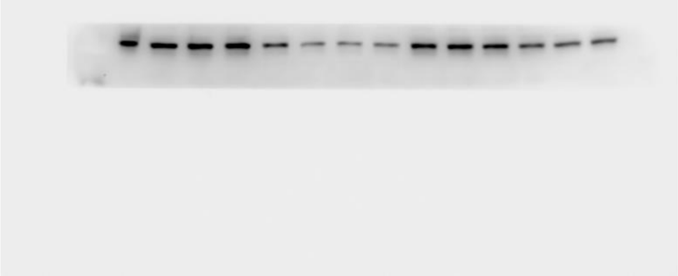

FECH

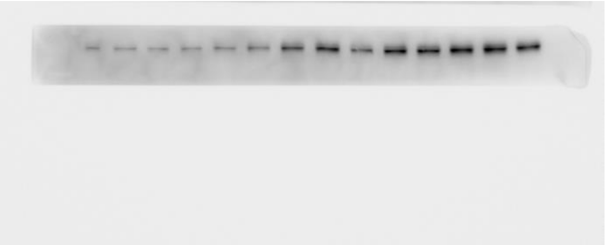

ATF4

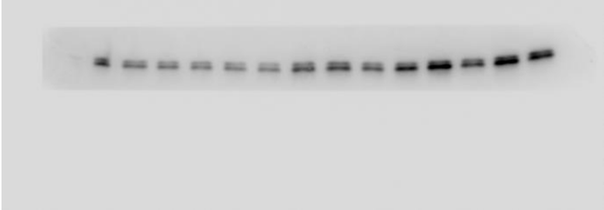

ATF3

Gel Loading Order (same for all blots):

1. Ladder
2. DMSO
3. +10 nM O/A
4. 0.3 μM FB231
5. 0.6 μM FB231
6. 1.25 μM FB231
7. 2.5 μM FB231
8. 5 μM FB231
9. 10 μM FB231
10. 0.3 μM FB231 + 10 nM O/A
11. 0.6 μM FB231 + 10 nM O/A
12. 1.25 μM FB231 + 10 nM O/A
13. 2.5 μM FB231 + 10 nM O/A
14. 5 μM FB231 + 10 nM O/A
15. 10 μM FB231 + 10 nM O/A

Fig 4G-H (YFP-Parkin-expressing cell lines), Fig S7B (WT): Compound dose escalation mitophagy and stress – MTK458 Repl. 1

Gel Loading Order (same for all blots):

1. Ladder
2. DMSO
3. +10 nM O/A
4. 0.6 µM MTK458
5. 1.25 µM MTK458
6. 2.5 µM MTK458
7. 5 µM MTK458
8. 10 µM MTK458
9. 20 µM MTK458
10. 0.6 µM MTK458 + 10 nM O/A
11. 1.25 µM MTK458 + 10 nM O/A
12. 2.5 µM MTK458 + 10 nM O/A
13. 5 µM MTK458 + 10 nM O/A
14. 10 µM MTK458 + 10 nM O/A
15. 20 µM MTK458 + 10 nM O/A

+YFP-Parkin

+YFP-Parkin /PINK1KO

WT

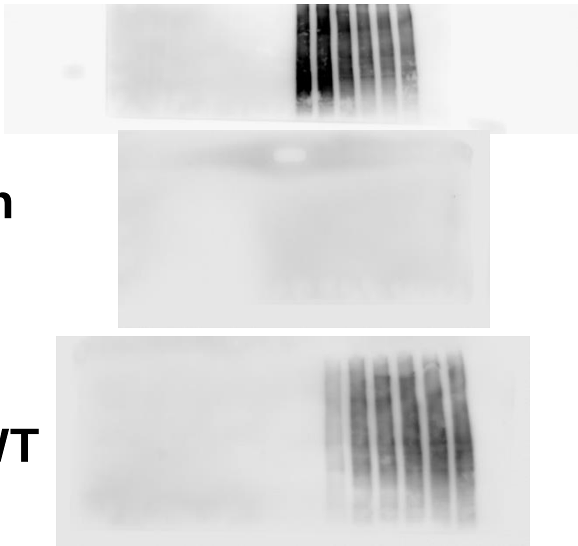

pUb

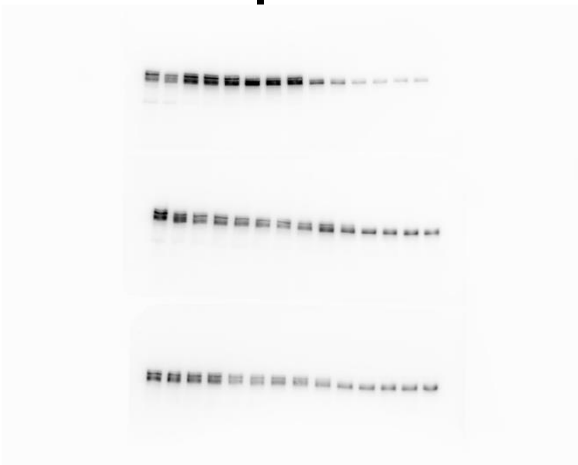

OPA1

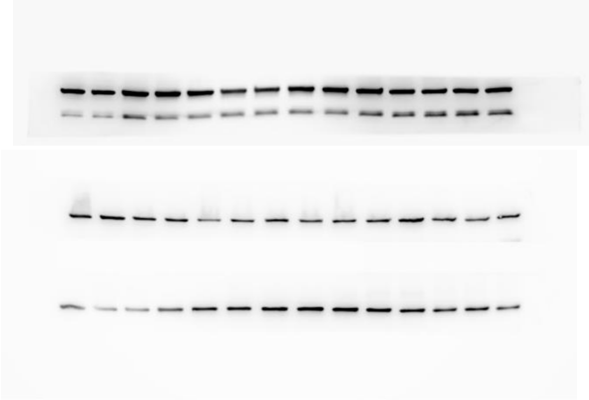

ATP5A

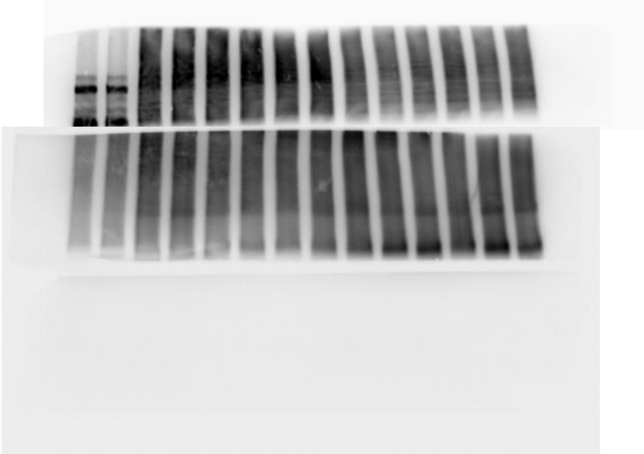

PARKIN

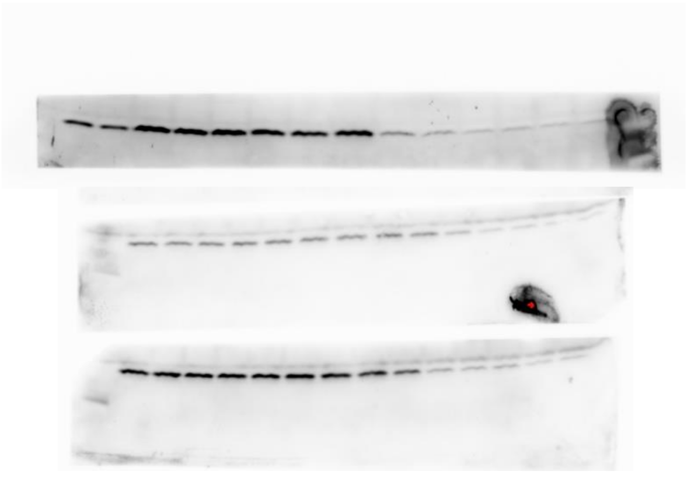

COX4I2

Fig 4G-H (YFP-Parkin-expressing cell lines), Fig S7B (WT): Compound dose escalation (continued) – MTK458 Repl. 1

+YFP-Parkin

+YFP-Parkin /PINK1KO

WT

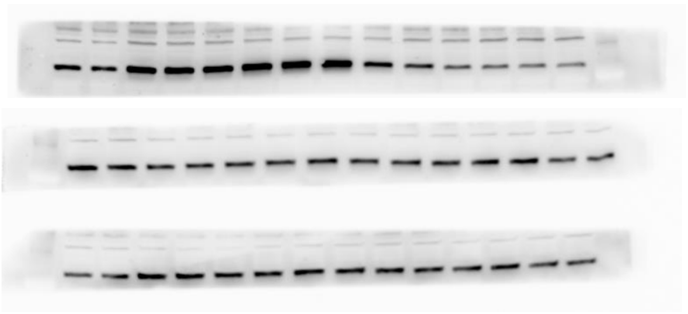

FECH

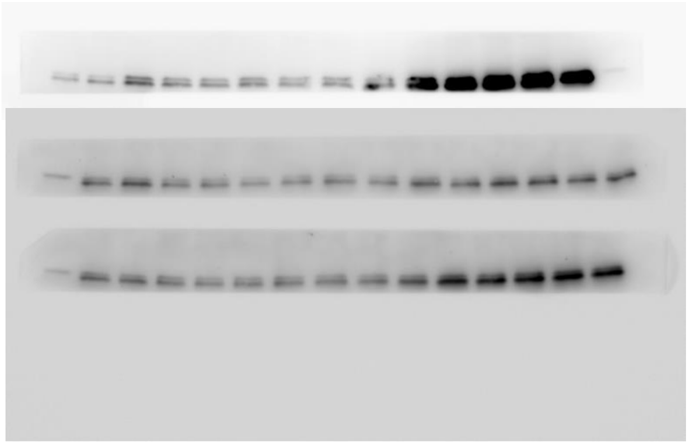

ATF3

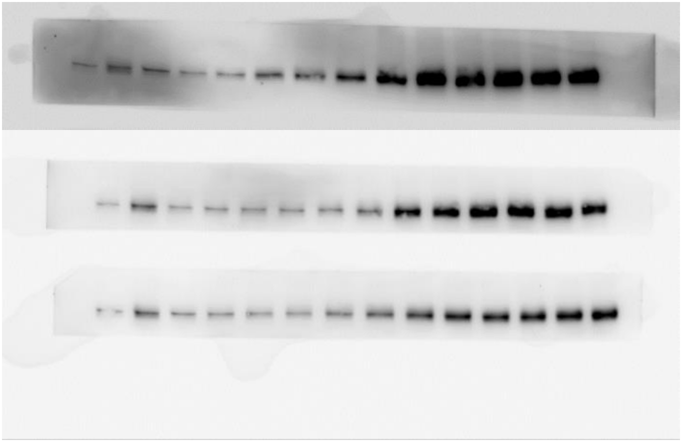

ATF4

Gel Loading Order (same for all blots):

1. DMSO
2. +10 nM O/A
3. 0.6 μM MTK458
4. 1.25 μM MTK458
5. 2.5 μM MTK458
6. 5 μM MTK458
7. 10 μM MTK458
8. 20 μM MTK458
9. 0.6 μM MTK458 + 10 nM O/A
10. 1.25 μM MTK458 + 10 nM O/A
11. 2.5 μM MTK458 + 10 nM O/A
12. 5 μM MTK458 + 10 nM O/A
13. 10 μM MTK458 + 10 nM O/A
14. 20 μM MTK458 + 10 nM O/A
15. Ladder

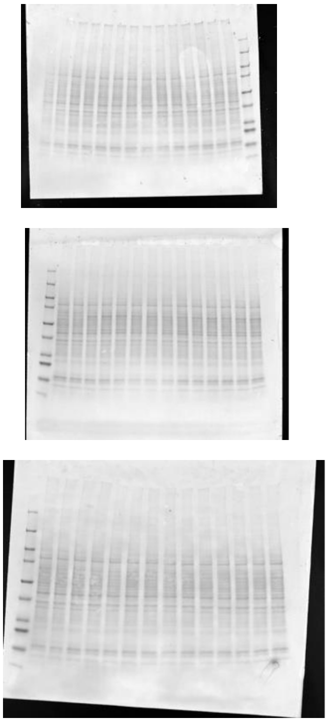

Ponceau S

Fig 4G-H (YFP-Parkin-expressing cell lines), Fig S7B (WT): Compound dose escalation mitophagy and stress – MTK458 Repl. 2

Gel Loading Order (same for all blots):

1. Ladder
2. DMSO
3. +10 nM O/A
4. 0.6 μM MTK458
5. 1.25 μM MTK458
6. 2.5 μM MTK458
7. 5 μM MTK458
8. 10 μM MTK458
9. 20 μM MTK458
10. 0.6 μM MTK458 + 10 nM O/A
11. 1.25 μM MTK458 + 10 nM O/A
12. 2.5 μM MTK458 + 10 nM O/A
13. 5 μM MTK458 + 10 nM O/A
14. 10 μM MTK458 + 10 nM O/A
15. 20 μM MTK458 + 10 nM O/A

+YFP-Parkin

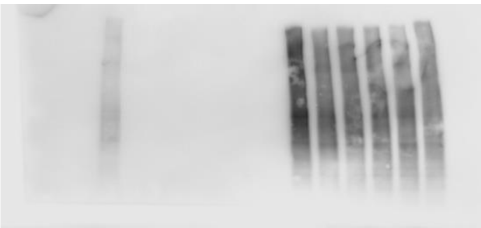

+YFP-Parkin /PINK1KO

WT

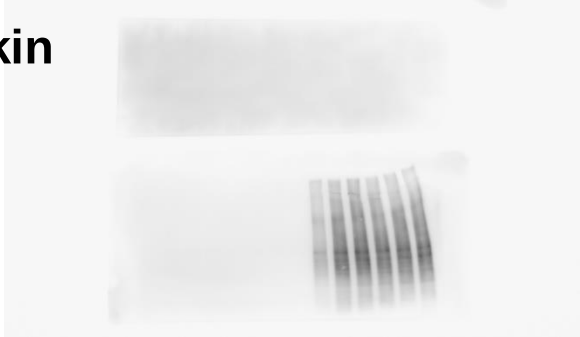

pUb

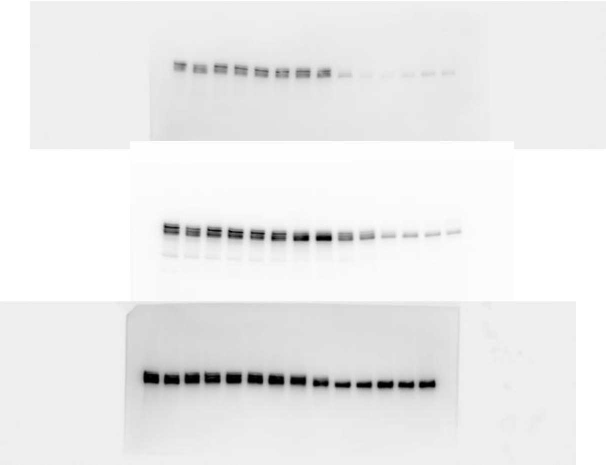

OPA1

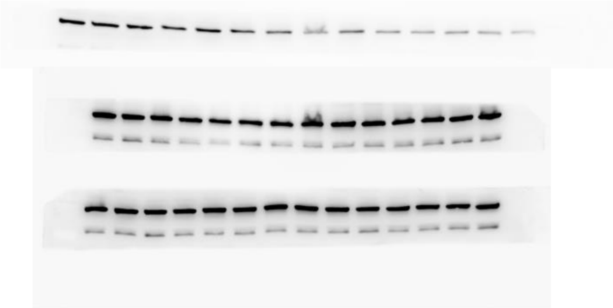

ATP5A

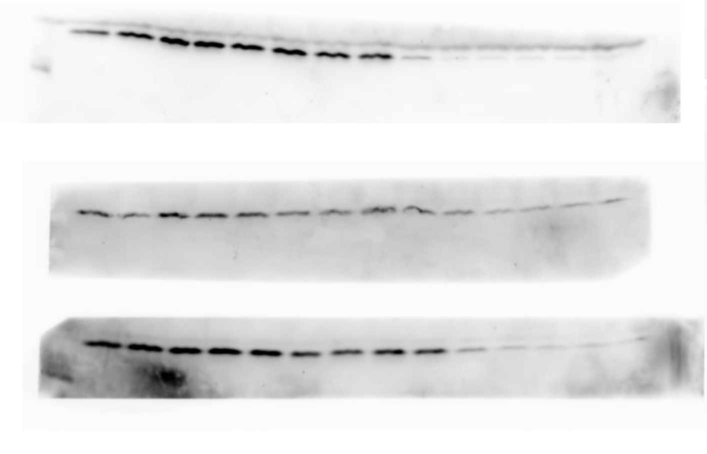

COX4I2

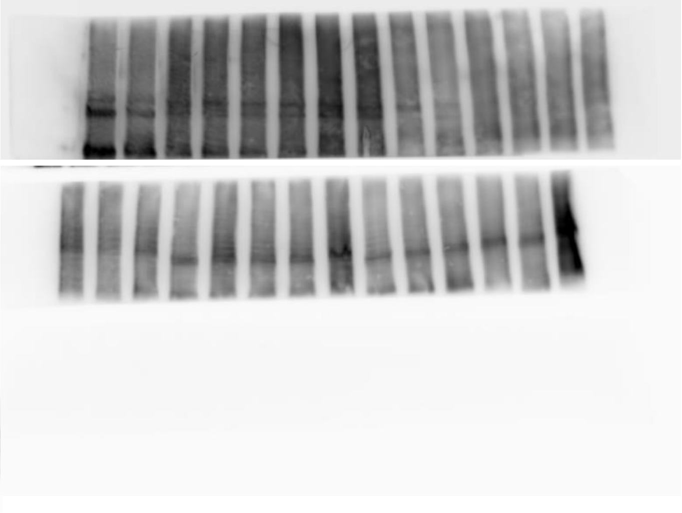

PARKIN

Fig 4G-H (YFP-Parkin-expressing cell lines), Fig S7B (WT): Compound dose escalation (continued) – MTK458 Repl. 2

+YFP-Parkin

+YFP-Parkin/PINK1KO

WT

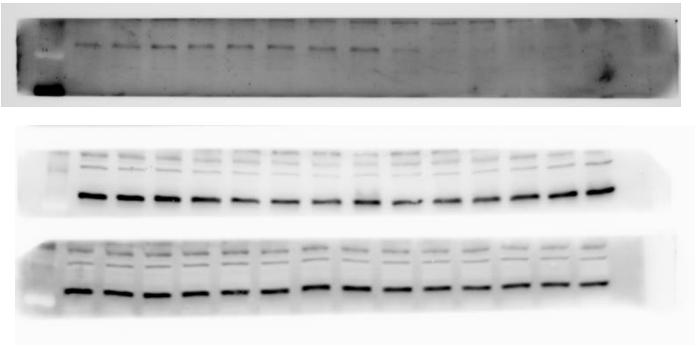

FECH

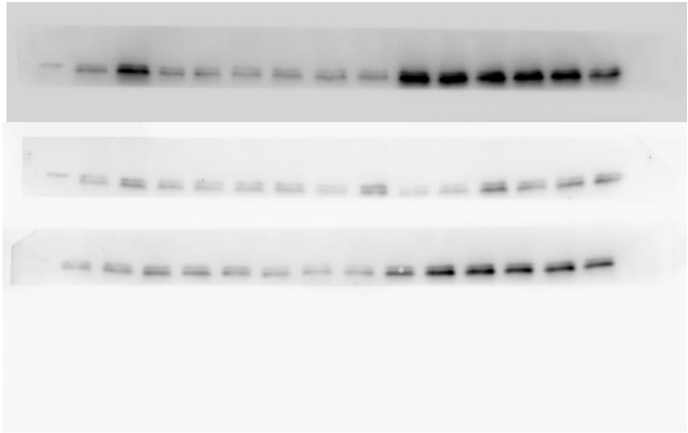

ATF3

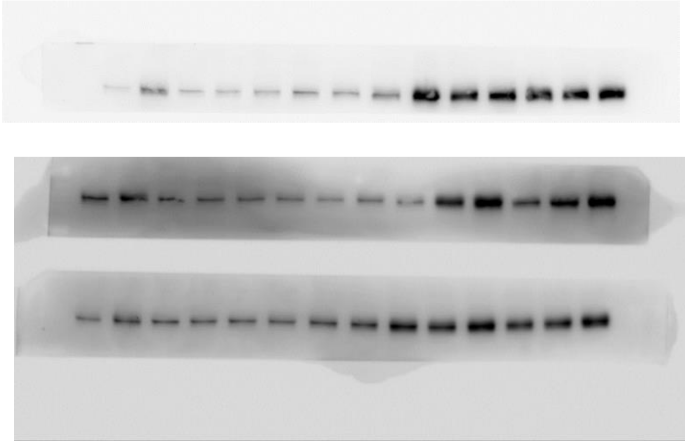

ATF4

Gel Loading Order (same for all blots):

1. Ladder
2. DMSO
3. +10 nM O/A
4. 0.6 µM MTK458
5. 1.25 µM MTK458
6. 2.5 µM MTK458
7. 5 µM MTK458
8. 10 µM MTK458
9. 20 µM MTK458
10. 0.6 µM MTK458 + 10 nM O/A
11. 1.25 µM MTK458 + 10 nM O/A
12. 2.5 µM MTK458 + 10 nM O/A
13. 5 µM MTK458 + 10 nM O/A
14. 10 µM MTK458 + 10 nM O/A
15. 20 µM MTK458 + 10 nM O/A

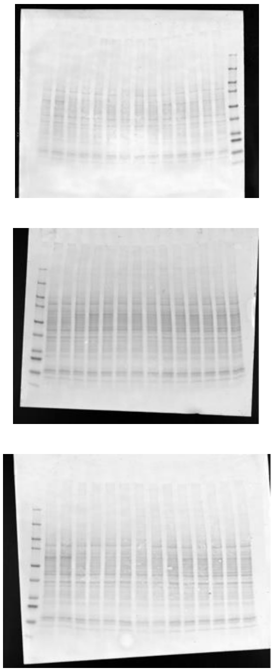

Ponceau S

Fig 4G-H: Compound dose escalation mitophagy and stress – MTK458 Repl. 3

Gel Loading Order (same for all blots):

1. Ladder
2. DMSO
3. +10 nM O/A
4. 0.6 µM MTK458
5. 1.25 µM MTK458
6. 2.5 µM MTK458
7. 5 µM MTK458
8. 10 µM MTK458
9. 20 µM MTK458
10. 0.6 µM MTK458 + 10 nM O/A
11. 1.25 µM MTK458 + 10 nM O/A
12. 2.5 µM MTK458 + 10 nM O/A
13. 5 µM MTK458 + 10 nM O/A
14. 10 µM MTK458 + 10 nM O/A
15. 20 µM MTK458 + 10 nM O/A

+YFP-Parkin

+YFP-Parkin /PINK1KO

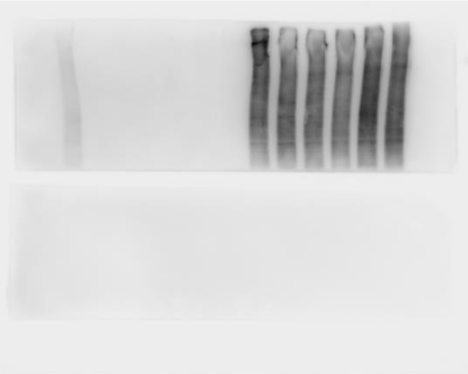

pUb

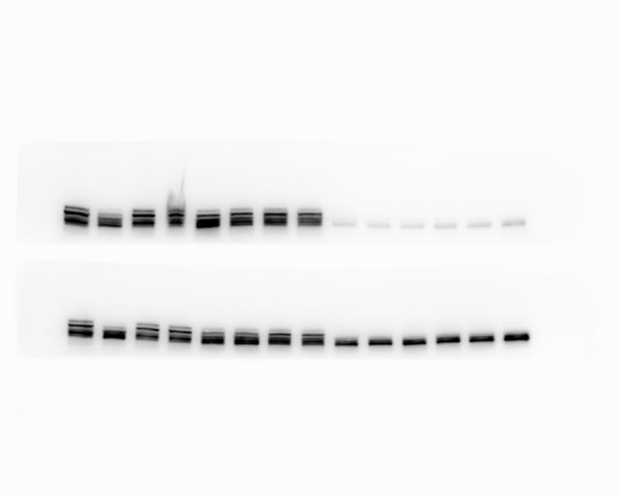

OPA1

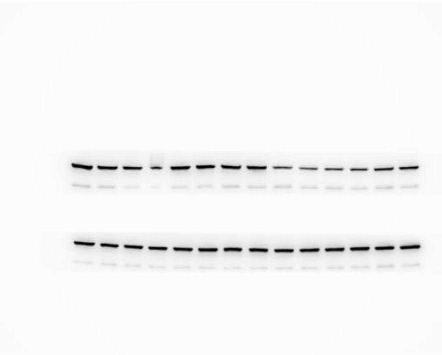

ATP5A

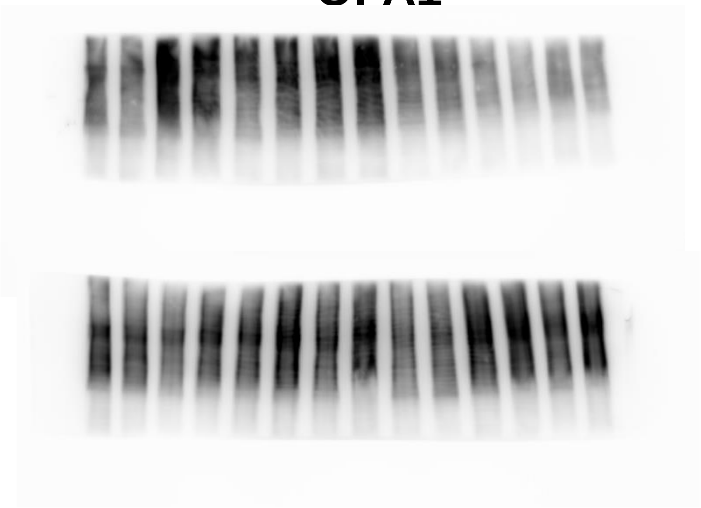

PARKIN

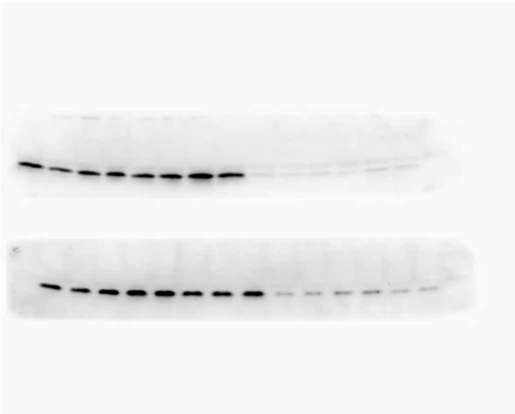

COX4I2

Fig 4G-H: Compound dose escalation (continued) – MTK458 Repl. 3

+YFP-Parkin

+YFP-Parkin /PINK1KO

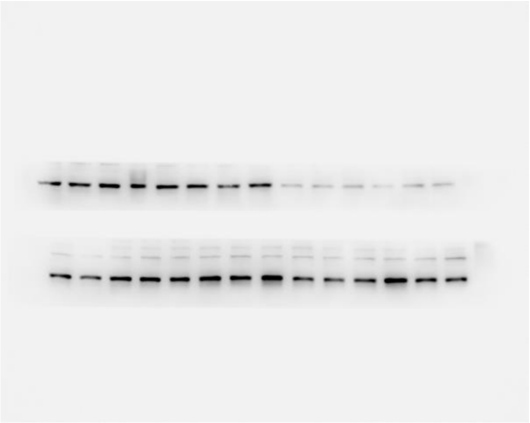

FECH

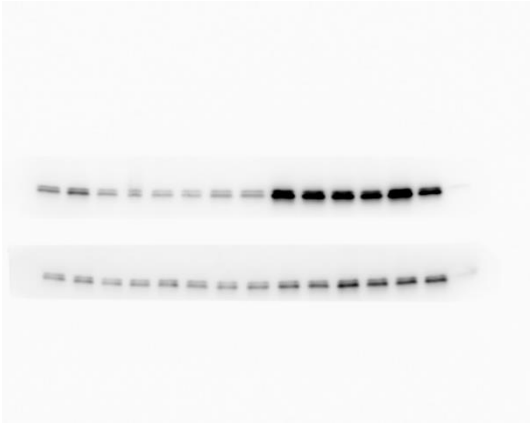

ATF3

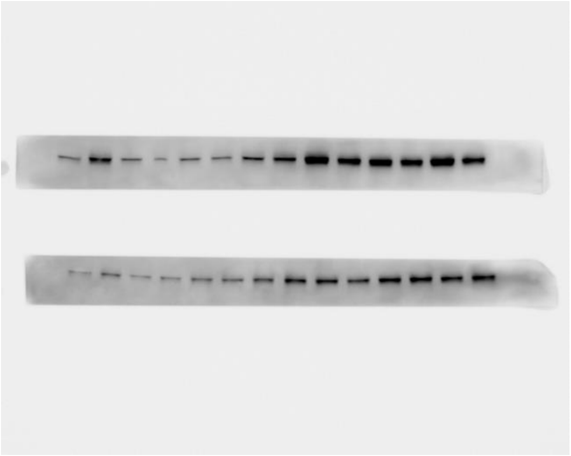

ATF4

Gel Loading Order (same for all blots):

1. DMSO
2. +10 nM O/A
3. 0.6 µM MTK458
4. 1.25 µM MTK458
5. 2.5 µM MTK458
6. 5 µM MTK458
7. 10 µM MTK458
8. 20 µM MTK458
9. 0.6 µM MTK458 + 10 nM O/A
10. 1.25 µM MTK458 + 10 nM O/A
11. 2.5 µM MTK458 + 10 nM O/A
12. 5 µM MTK458 + 10 nM O/A
13. 10 µM MTK458 + 10 nM O/A
14. 20 µM MTK458 + 10 nM O/A
15. Ladder

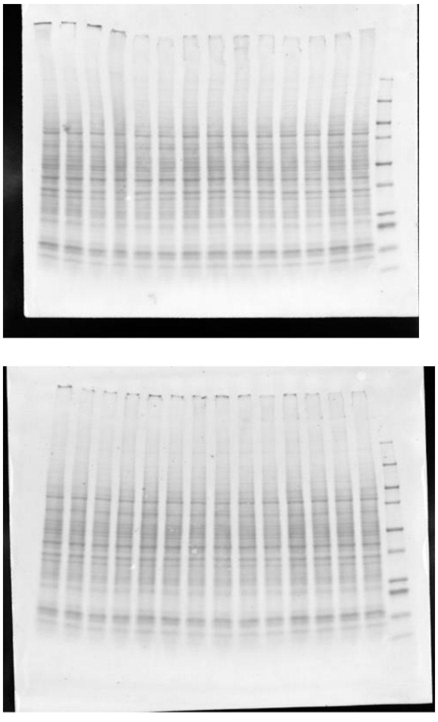

Ponceau S

Figure 4J: Mass Spec Compound off-targets – SH-SY5Y repl 1 & 3

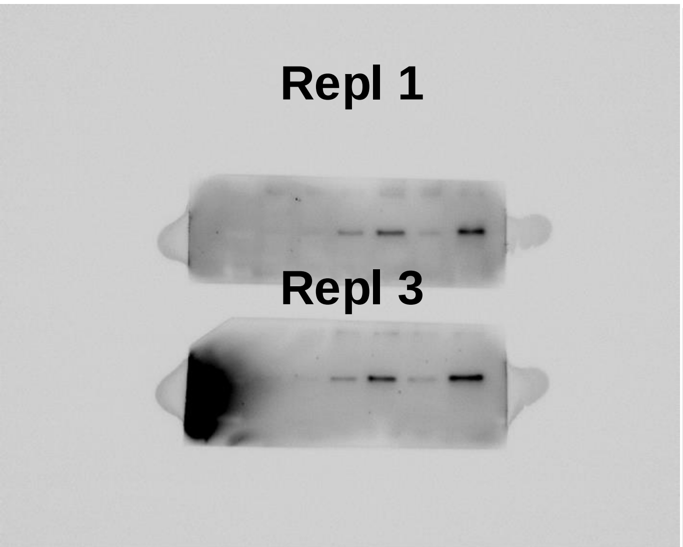

IFRD1

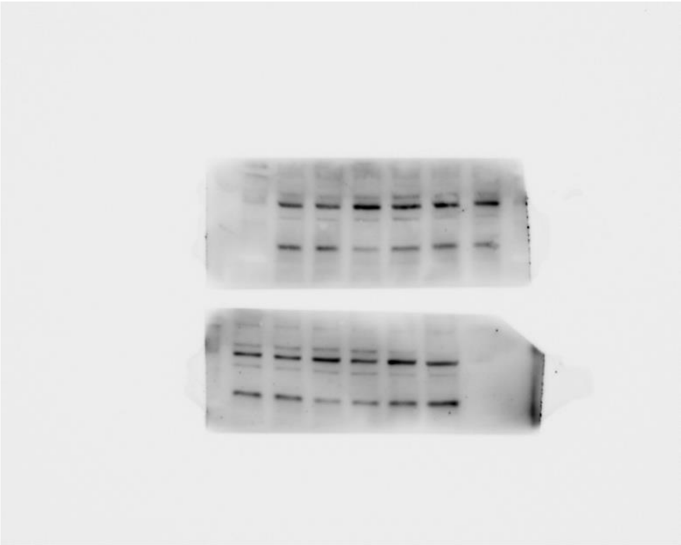

FECH

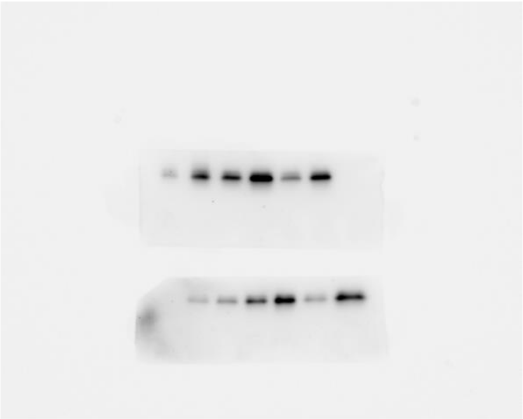

ATF3

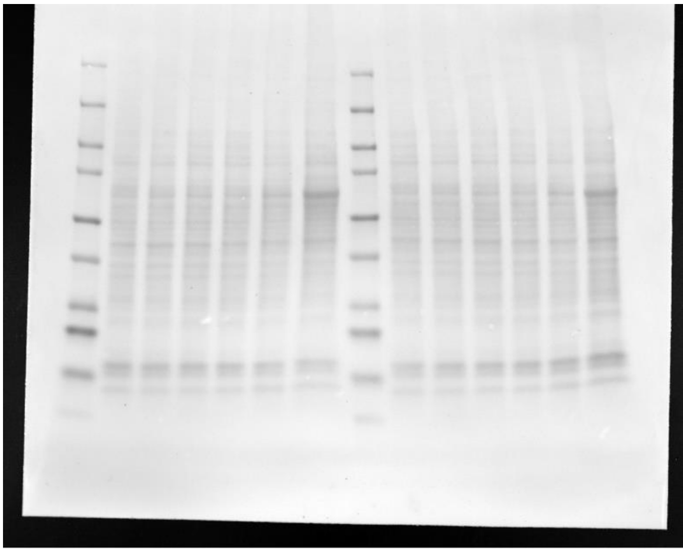

Ponceau S

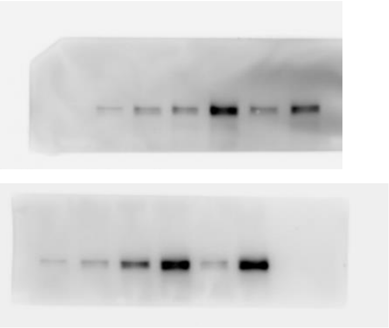

ATF4

Gel Loading Order (same for all blots):

1. Ladder
2. DMSO
3. +10 nM O/A
4. 10 μM FB231
5. 10 μM FB231 + 10 nM O/A
6. 5 μM MTK458
7. 5 μM MTK458 + 10 nM O/A

Figure 4J: Mass Spec Compound off-targets – SH-SY5Y repl 2 – (representative)

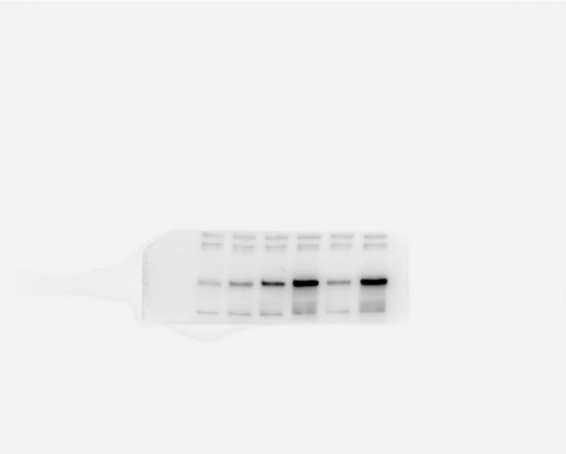

IFRD1

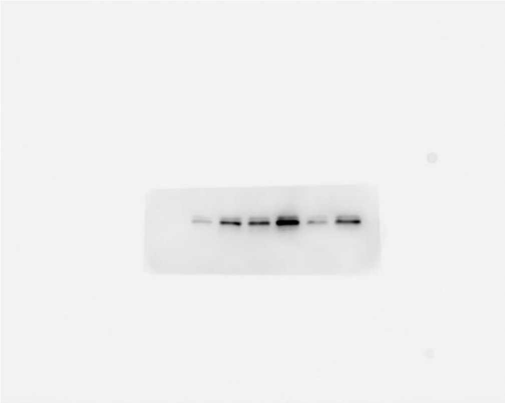

ATF3

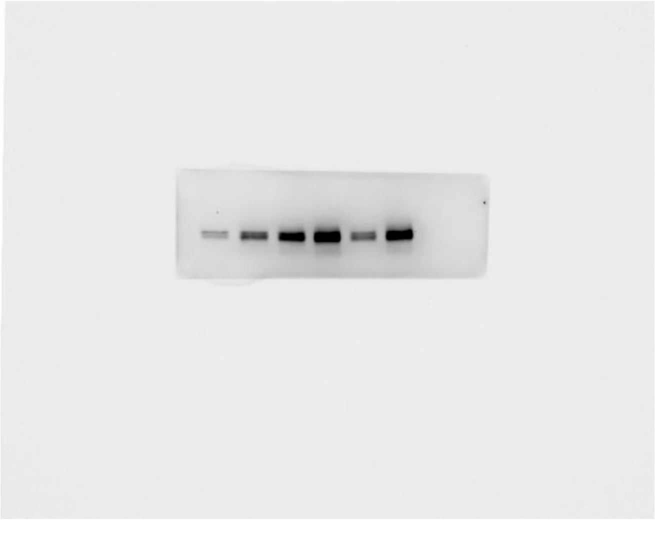

ATF4

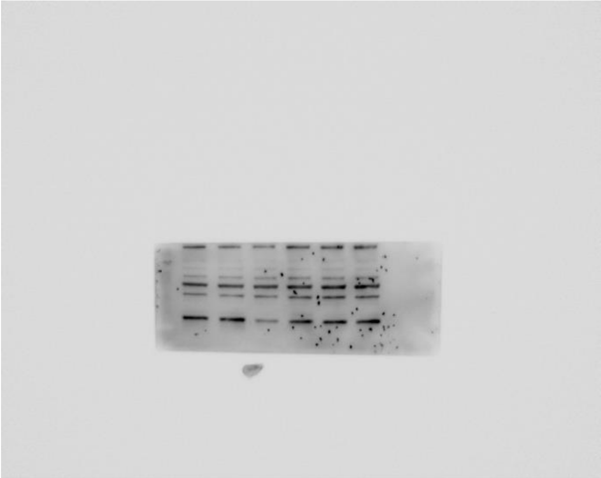

FECH

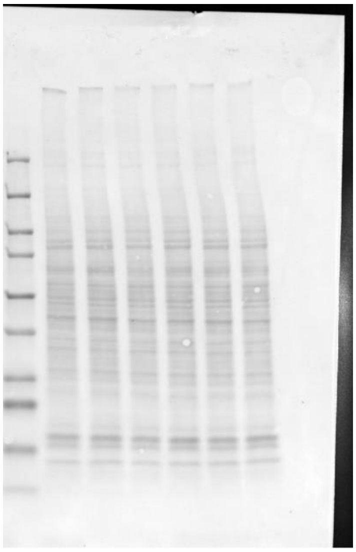

Ponceau S

Gel Loading Order (same for all blots):

1. Ladder
2. DMSO
3. +10 nM O/A
4. 10 μM FB231
5. 10 μM FB231 + 10 nM O/A
6. 5 μM MTK458
7. 5 μM MTK458 + 10 nM O/A

Figure 5B: Integrative stress response markers upon Mitophagy activator treatment in WT HeLa S3 – repl 1 (representative)

HA-DELE1

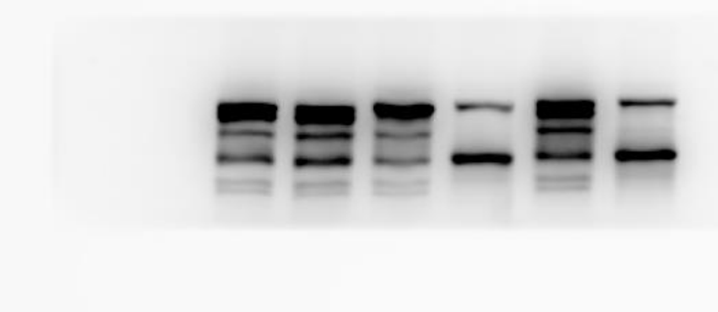

Gel Loading Order (same for all blots):

1. Ladder
2. DMSO
3. +10 nM O/A
4. 10 µM FB231
5. 10 µM FB231 + 10 nM O/A
6. 10 µM MTK458
7. 10 µM MTK458 + 10 nM O/A

ATF3

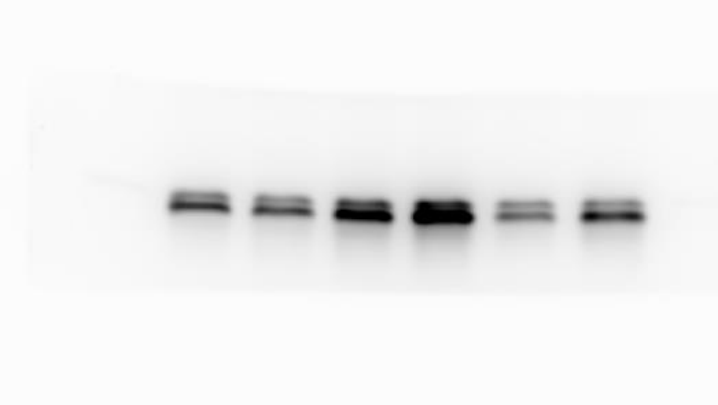

Ponceau S

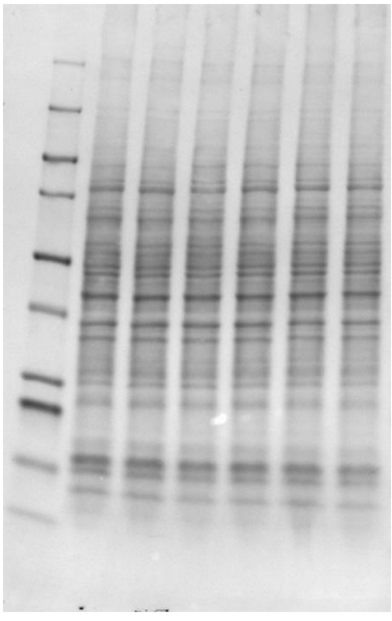

ATF4

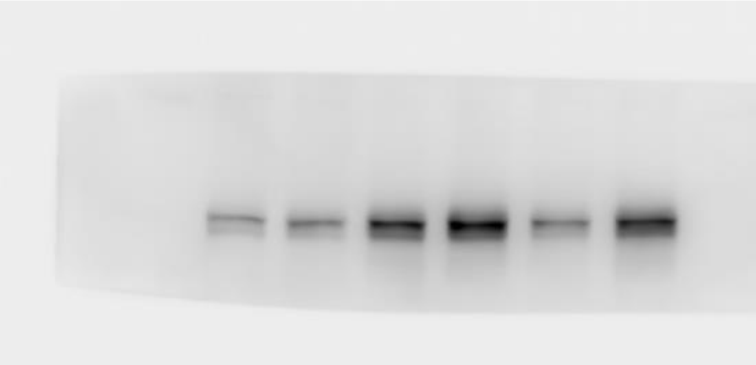

Figure 5B: Integrative stress response markers upon Mitophagy activator treatment in WT HeLa S3 – repl 2

HA-DELE1

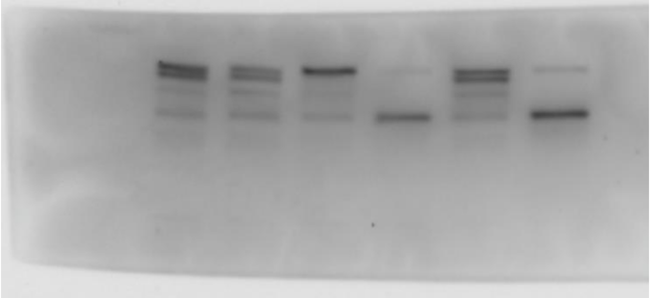

ATF3

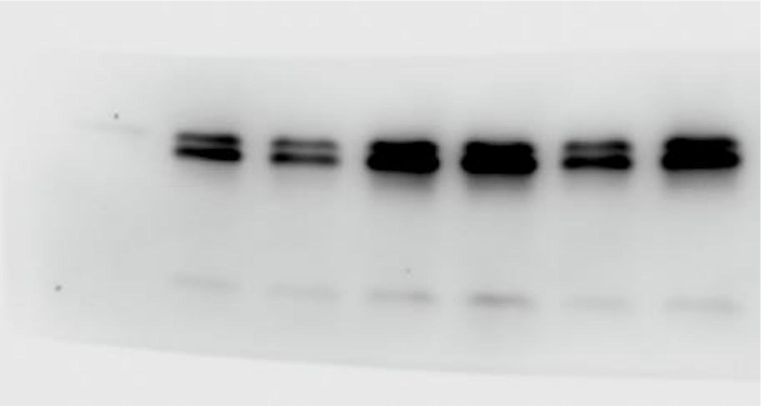

ATF4

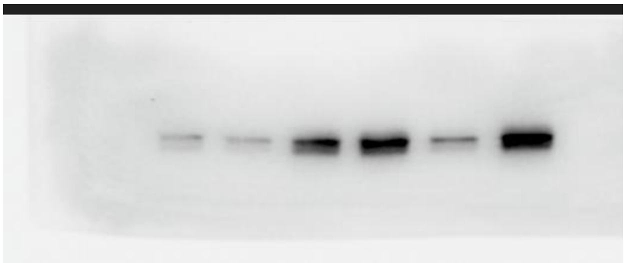

Gel Loading Order (same for all blots):

1. Ladder
2. DMSO
3. +10 nM O/A
4. 10 µM FB231
5. 10 µM FB231 + 10 nM O/A
6. 10 µM MTK458
7. 10 µM MTK458 + 10 nM O/A

Ponceau S

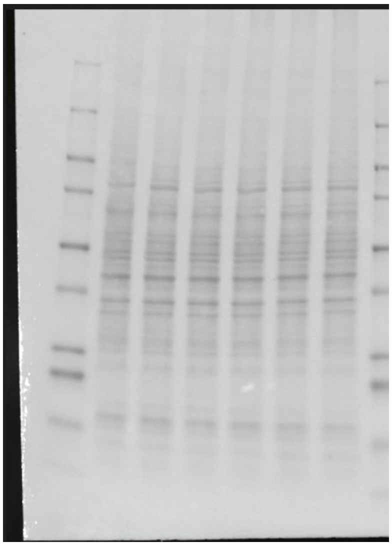

Figure 5B: Integrative stress response markers upon Mitophagy activator treatment in WT HeLa S3 – repl 3

HA-DELE1

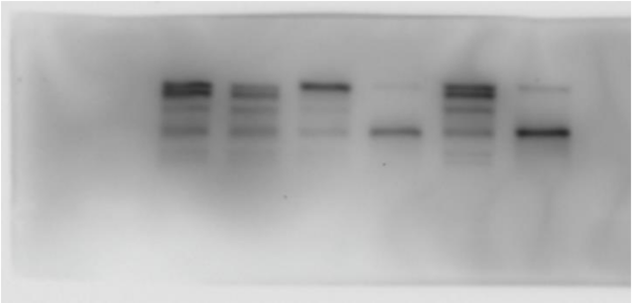

ATF3

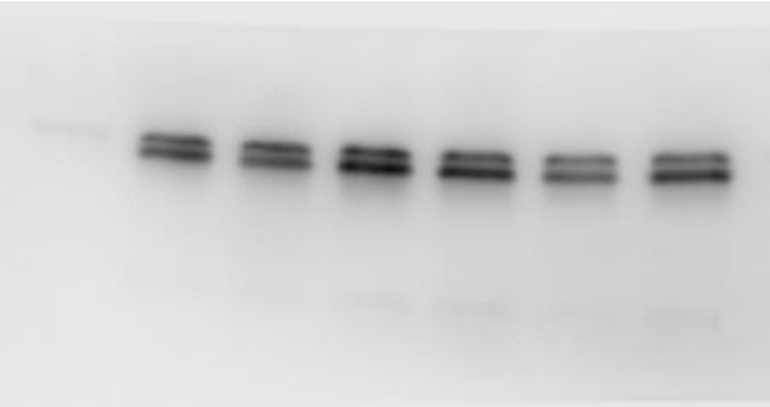

ATF4

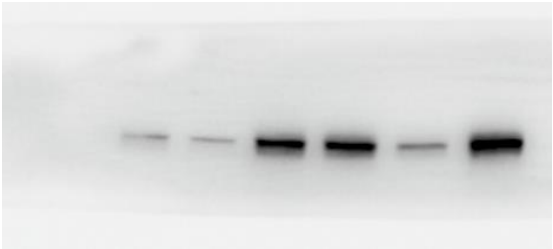

Gel Loading Order (same for all blots):

1. Ladder
2. DMSO
3. +10 nM O/A
4. 10 µM FB231
5. 10 µM FB231 + 10 nM O/A
6. 10 µM MTK458
7. 10 µM MTK458 + 10 nM O/A

Ponceau S

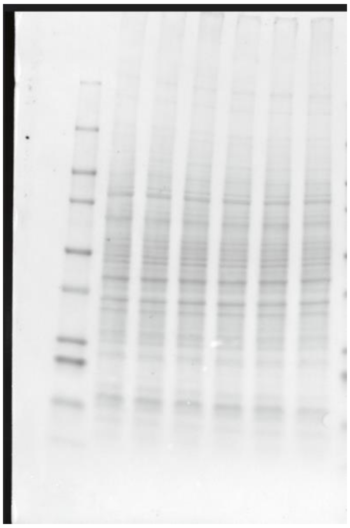

Figure 5C: Integrative stress response markers upon Mitophagy activator treatment in +YFP-Parkin/PINK1KO HeLa S3 – repl 1 (representative)

HA-DELE1

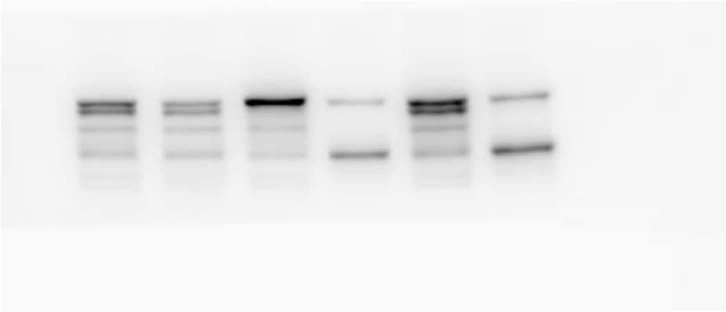

Gel Loading Order (same for all blots):

1. Ladder
2. DMSO
3. +10 nM O/A
4. 10 µM FB231
5. 10 µM FB231 + 10 nM O/A
6. 10 µM MTK458
7. 10 µM MTK458 + 10 nM O/A

ATF3

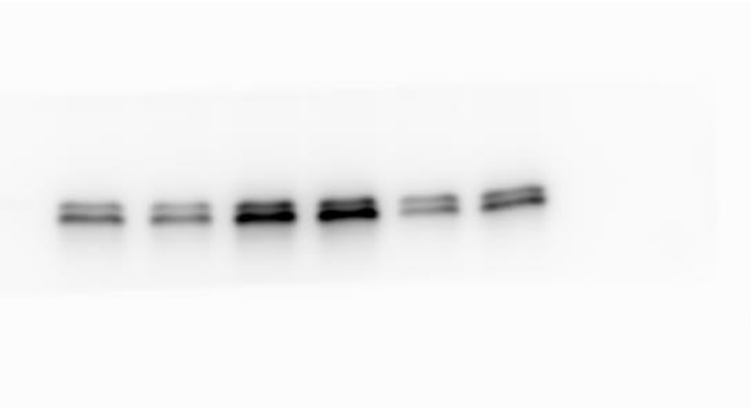

ATF4

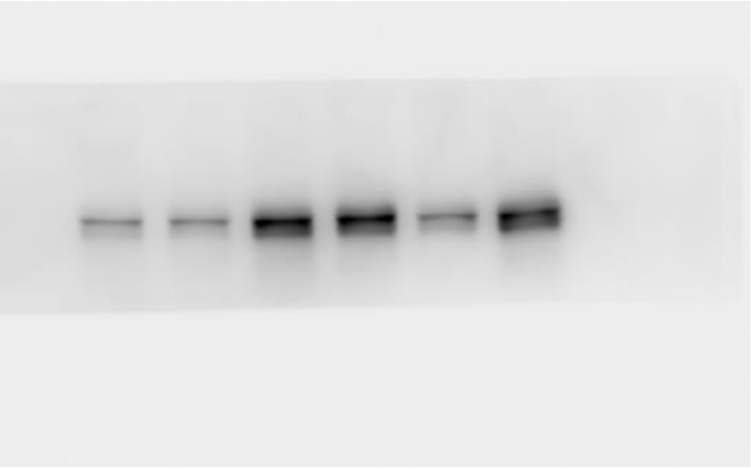

Ponceau S

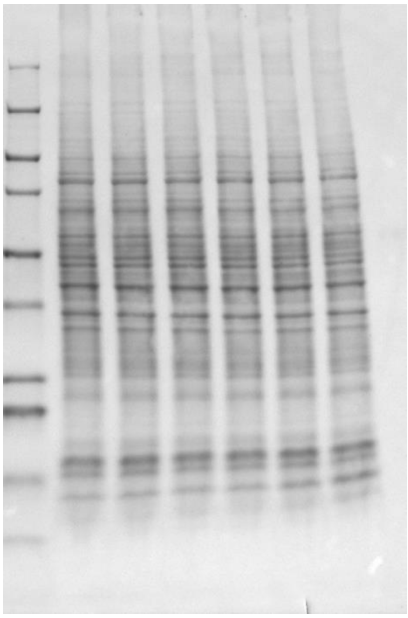

Figure 5C: Integrative stress response markers upon Mitophagy activator treatment in +YFP-Parkin/PINK1KO HeLa S3 – repl 2

HA-DELE1

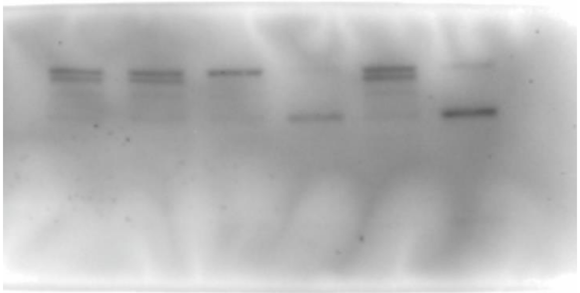

ATF3

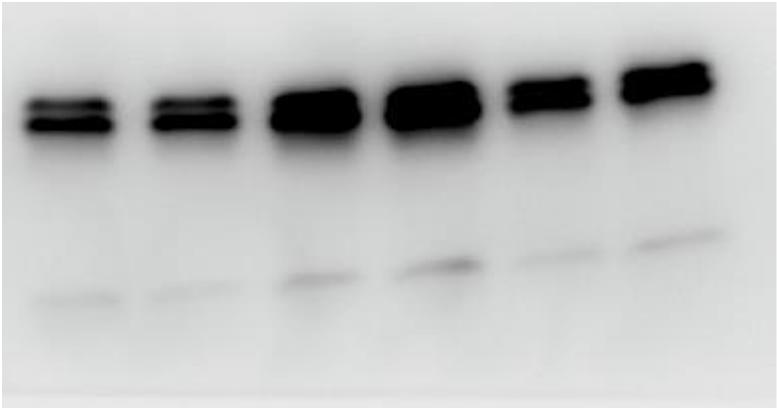

ATF4

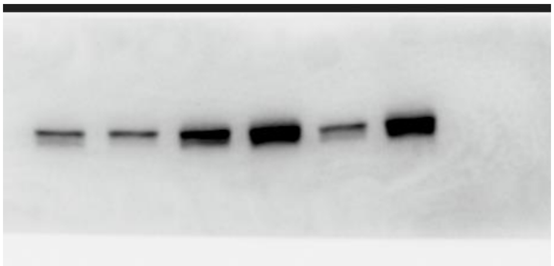

Gel Loading Order (same for all blots):

1. Ladder
2. DMSO
3. +10 nM O/A
4. 10 µM FB231
5. 10 µM FB231 + 10 nM O/A
6. 10 µM MTK458
7. 10 µM MTK458 + 10 nM O/A

Ponceau S

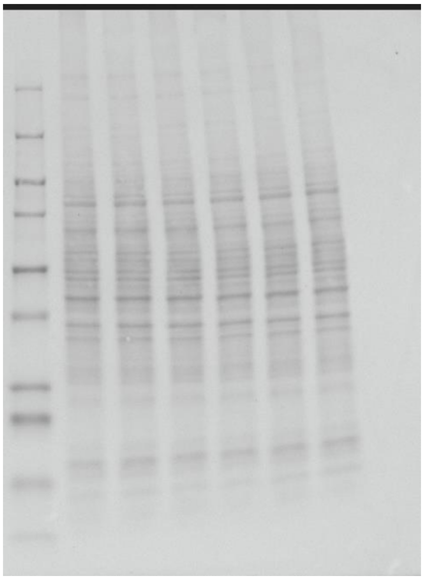

Figure 5C: Integrative stress response markers upon Mitophagy activator treatment in +YFP-Parkin/PINK1KO HeLa S3 – repl 3

HA-DELE1

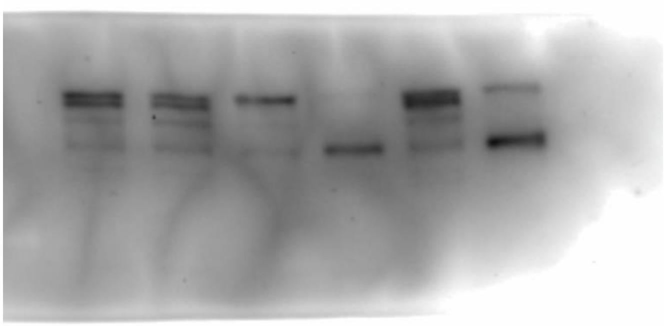

ATF3

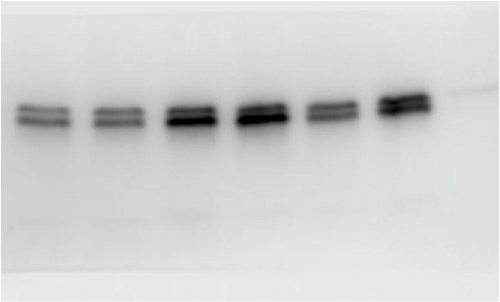

Gel Loading Order (same for all blots):

1. Ladder
2. DMSO
3. +10 nM O/A
4. 10 μM FB231
5. 10 μM FB231 + 10 nM O/A
6. 10 μM MTK458
7. 10 μM MTK458 + 10 nM O/A

ATF4

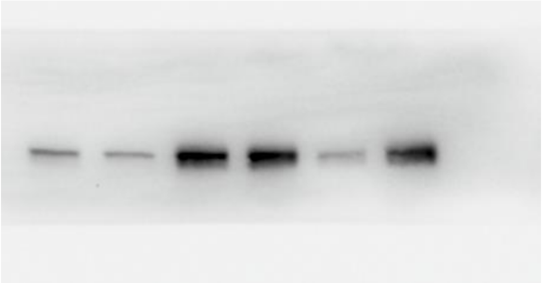

Ponceau S

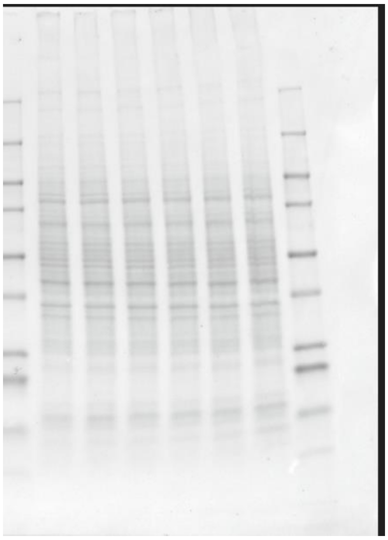

Fig 5G: ISR knockdowns confirmation repl. 1 (representative)

HRI

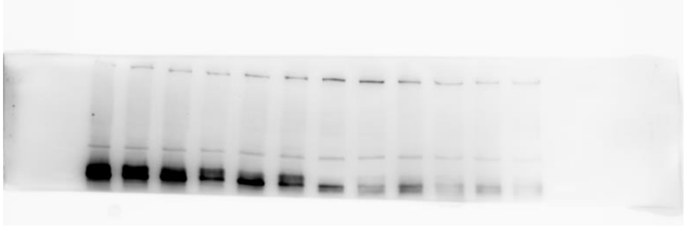

ATF3

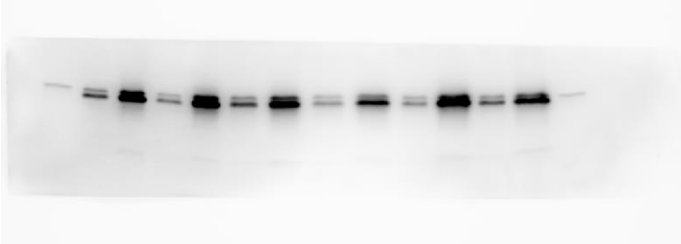

ATF4

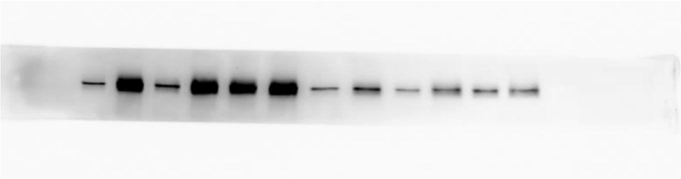

Ponceau S

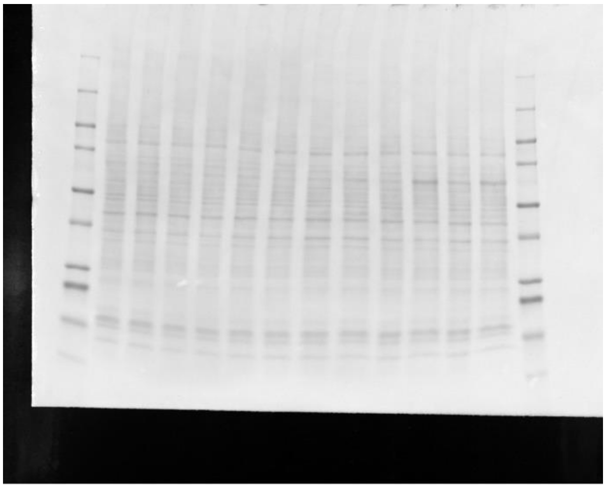

Gel Loading Order (same for all blots):

1. Ladder
2. Vector DMSO
3. Vector 10 nM O/A
4. Vector 5 µM MTK458
5. Vector 5 µM MTK458 + 10nM O/A
6. Vector 2.5 µM FB231
7. Vector 2.5 µM FB231 + 10nM O/A
8. shHRI DMSO
9. shHRI 10 nM O/A
10. shHRI 5 µM MTK458
11. shHRI 5 µM MTK458 + 10nM O/A
12. shHRI 2.5 µM FB231
13. shHRI 2.5 µM FB231 + 10nM O/A
14. Ladder

Fig 5G: ISR knockdowns confirmation (FB231)

Repl. 2

HRI

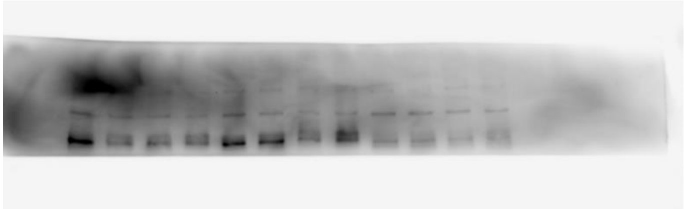

ATF3

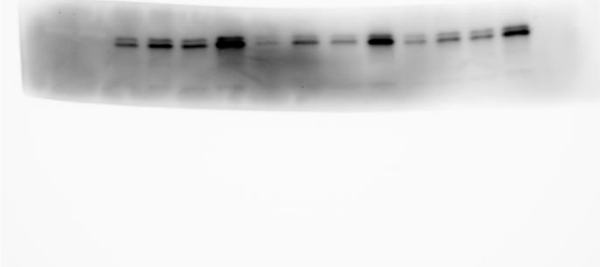

ATF4

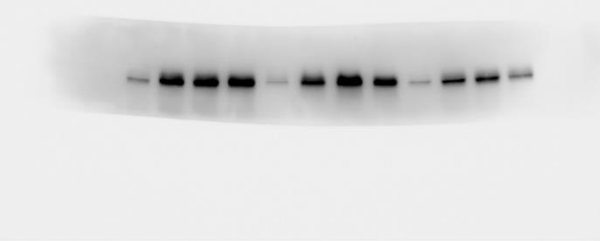

Ponceau S

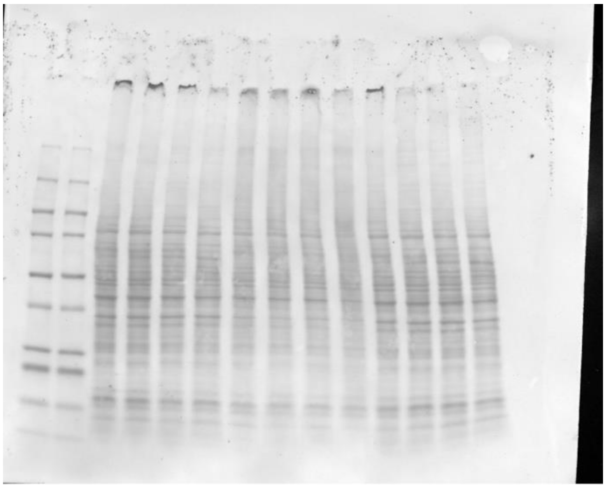

Gel Loading Order (same for all blots):

1. Ladder
2. Ladder
3. Vector DMSO
4. Vector 10 nM O/A
5. Vector 2.5 μM FB231
6. Vector 2.5 μM FB231 + 10nM O/A
7. shDELE1 DMSO
8. shDELE1 10 nM O/A
9. shDELE1 2.5 μM FB231
10. shDELE1 2.5 μM FB231 + 10nM O/A
11. shHRI DMSO
12. shHRI 10 nM O/A
13. shHRI 2.5 μM FB231
14. shHRI 2.5 μM FB231 + 10nM O/A

Fig 5G: ISR knockdowns confirmation (MTK458)

Repl. 2

HRI

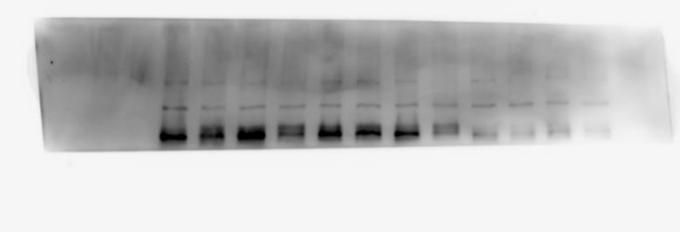

ATF3

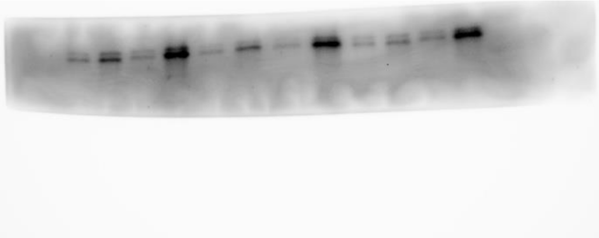

ATF4

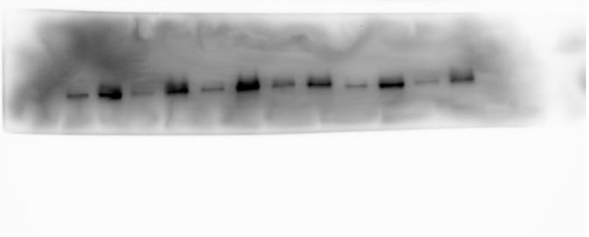

Ponceau S

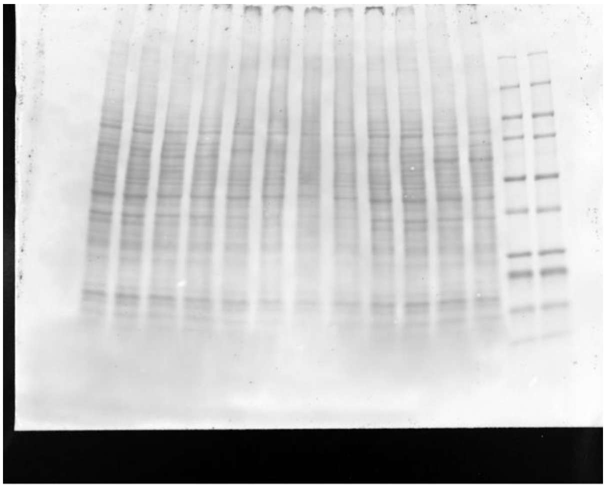

Gel Loading Order (same for all blots):

1. Vector DMSO
2. Vector 10 nM O/A
3. Vector 5 μM MTK458
4. Vector 5 μM MTK458 + 10nM O/A
5. shDELE1 DMSO
6. shDELE1 10 nM O/A
7. shDELE1 5 μM MTK458
8. shDELE1 5 μM MTK458 + 10nM O/A
9. shHRI DMSO
10. shHRI 10 nM O/A
11. shHRI 5 μM MTK458
12. shHRI 5 μM MTK458 + 10nM O/A
13. Ladder
14. Ladder

Figure 6B: Iron responsive element binding protein 2 Upon FB231 treatment in YFP-Parkin-expressing HeLa cells

Repl 1

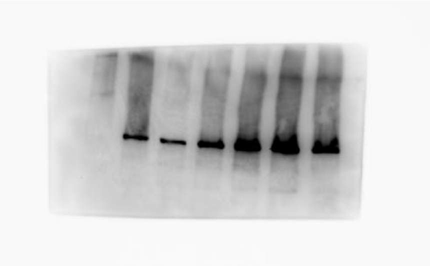

IREB2

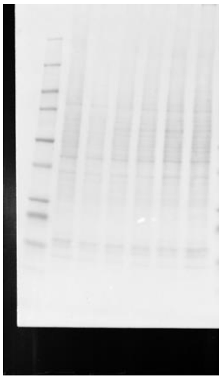

Ponceau S

Repl 2

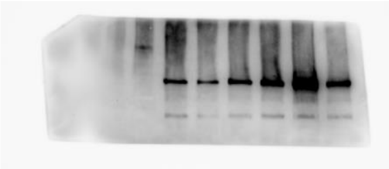

IREB2

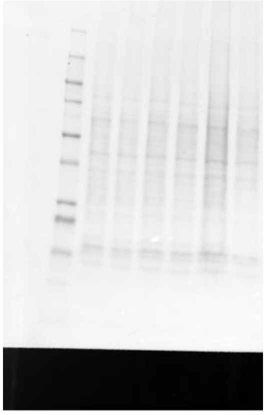

Ponceau S

Repl 3

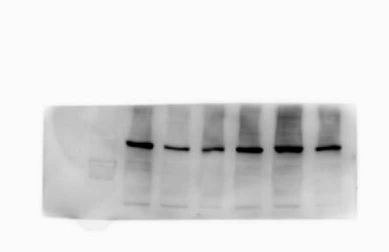

IREB2

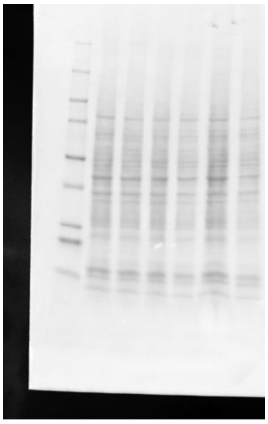

Ponceau S

Gel Loading Order (same for all blots):

1. Ladder
2. DMSO
3. 10 nM O/A
4. 0.6 μM FB231
5. 2.5 μM FB231
6. 10 μM FB231
7. 2.5 μM FB231 + 10 nM O/A

Figure S3: Activators + BafA treatment repl. 1 - representative

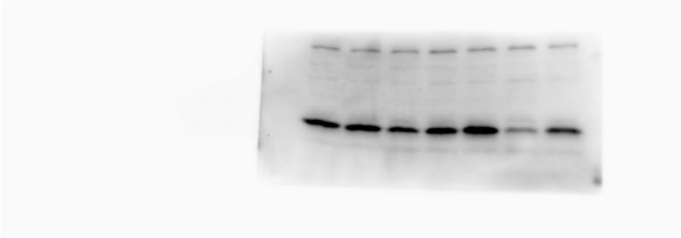

COX4I2

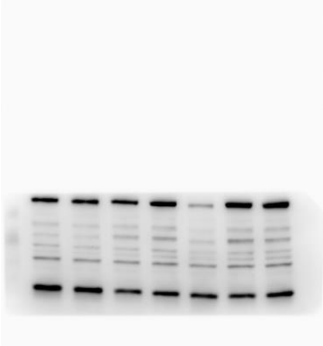

FECH

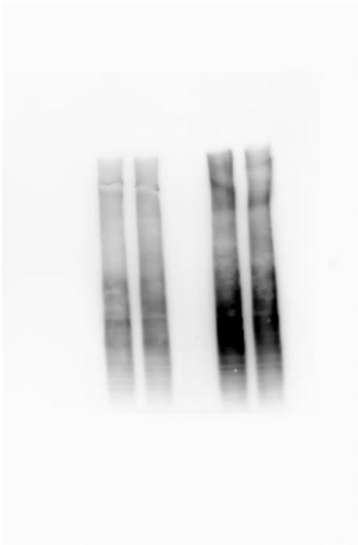

pUb

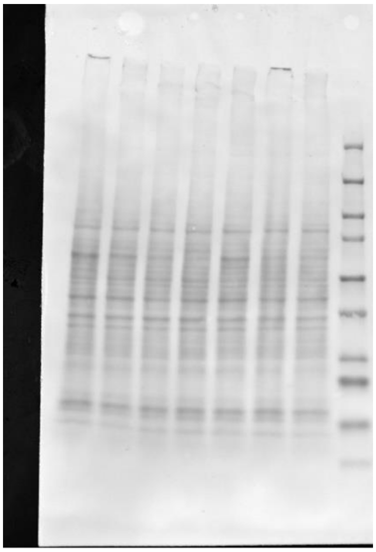

Ponceau S

Gel Loading Order (same for all blots):

1. DMSO
2. 2.5 µM MTK458
3. 2.5 µM MTK458 + 10 nM O/A
4. 2.5 µM MTK458 + 10 nM O/A + 100 nM BafA
5. 2.5 µM FB231
6. 2.5 µM FB231 + 10 nM O/A
7. 2.5 µM FB231 + 10 nM O/A + 100 nM BafA
8. Ladder

Figure S3: Activators + BafA treatment repl. 2

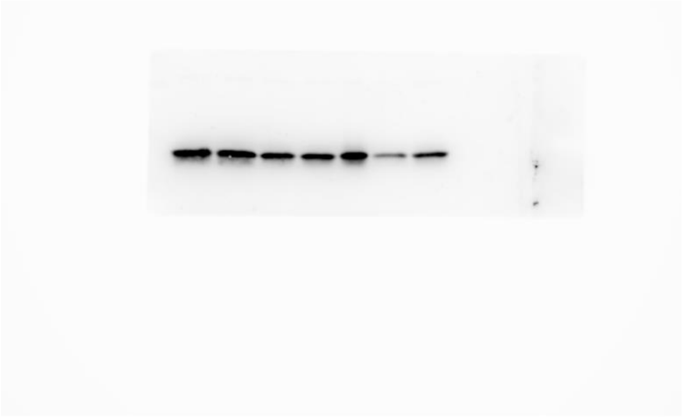

COX4I2

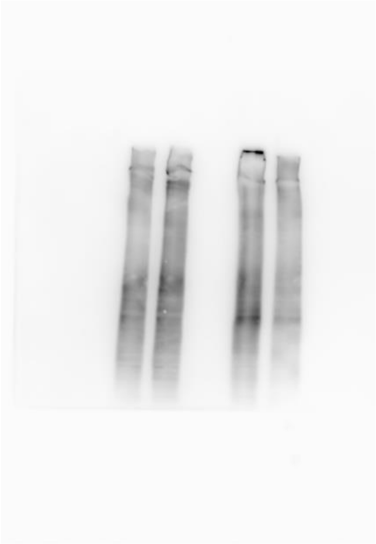

pUb

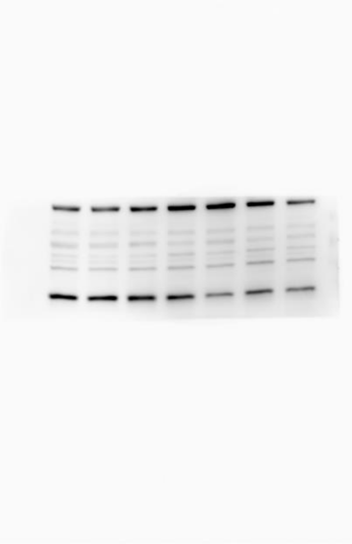

FECH

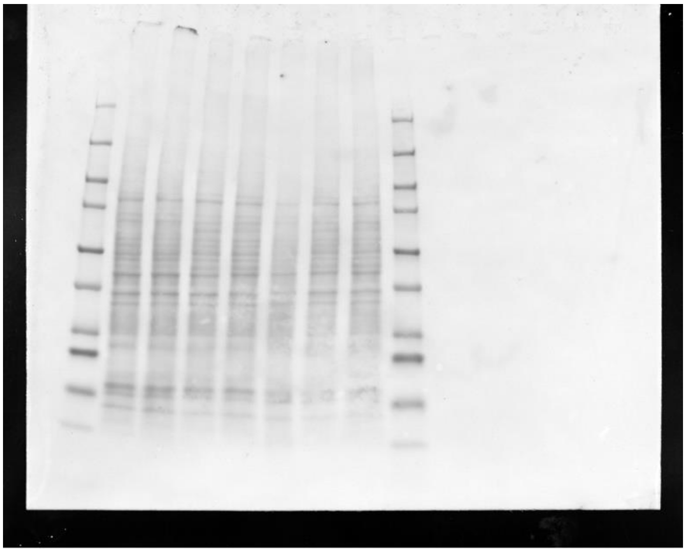

Ponceau S

Gel Loading Order (same for all blots):

1. Ladder
2. DMSO
3. 2.5 µM MTK458
4. 2.5 µM MTK458 + 10 nM O/A
5. 2.5 µM MTK458 + 10 nM O/A + 100 nM BafA
6. 2.5 µM FB231
7. 2.5 µM FB231 + 10 nM O/A
8. 2.5 µM FB231 + 10 nM O/A + 100 nM BafA
9. Ladder

Figure S6A-B: Mass Spec Compound off-targets – repl 1

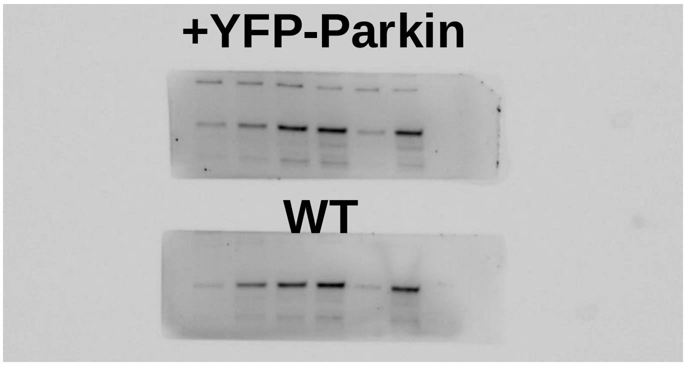

IFRD1

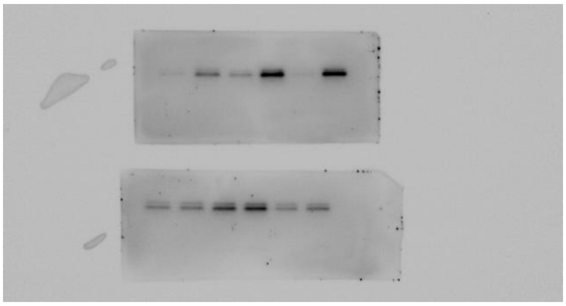

ATF3

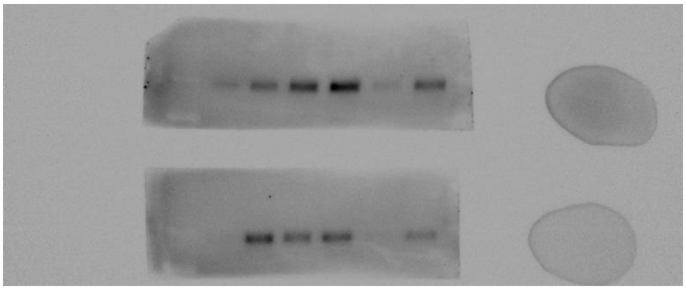

ATF4

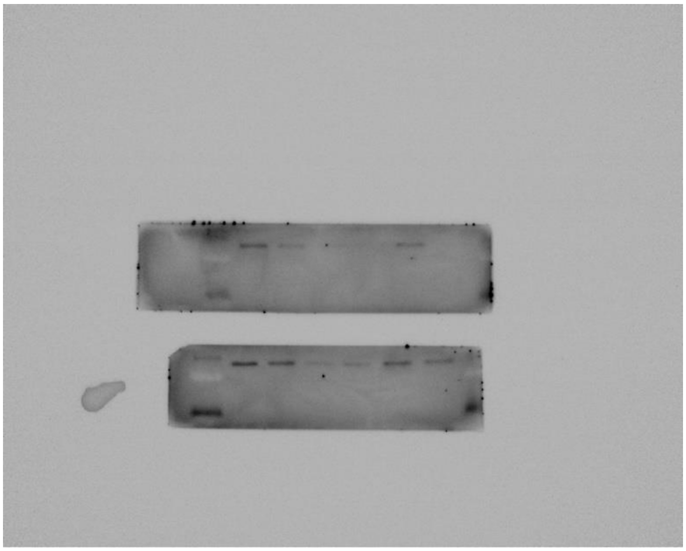

FECH

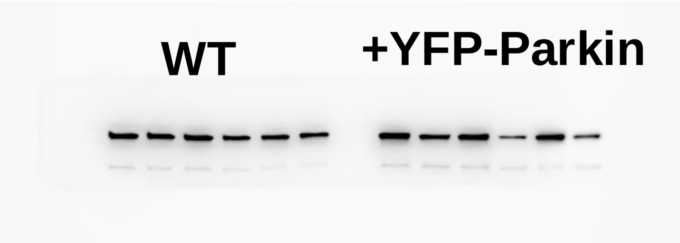

ATP5A

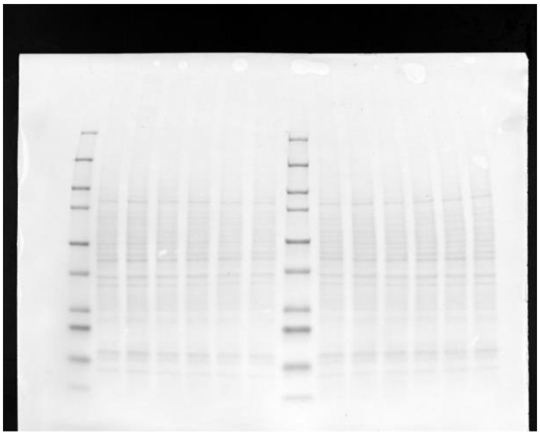

Ponceau S

Gel Loading Order (same for all blots):

1. Ladder
2. DMSO
3. +10 nM O/A
4. 10 µM FB231
5. 10 µM FB231 + 10 nM O/A
6. 5 µM MTK458
7. 5 µM MTK458 + 10 nM O/A

Figure S6A-B: Mass Spec Compound off-targets – repl 2

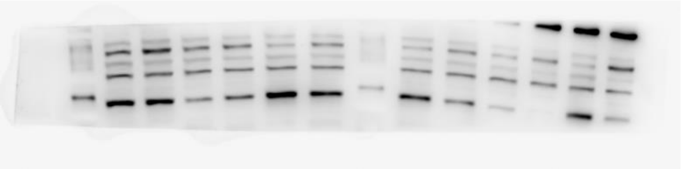

FECH

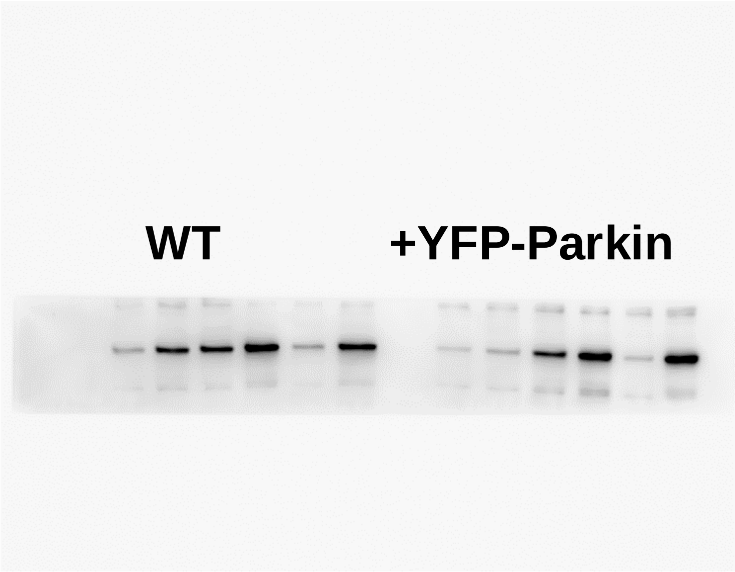

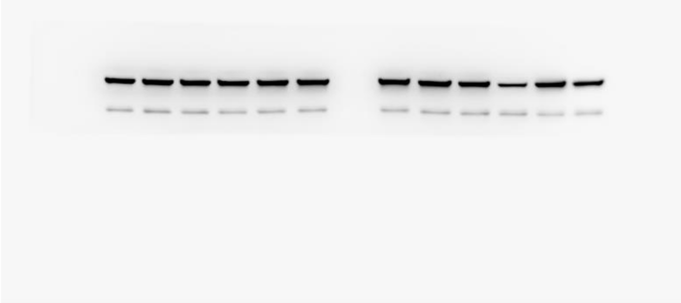

ATP5A

IFRD1

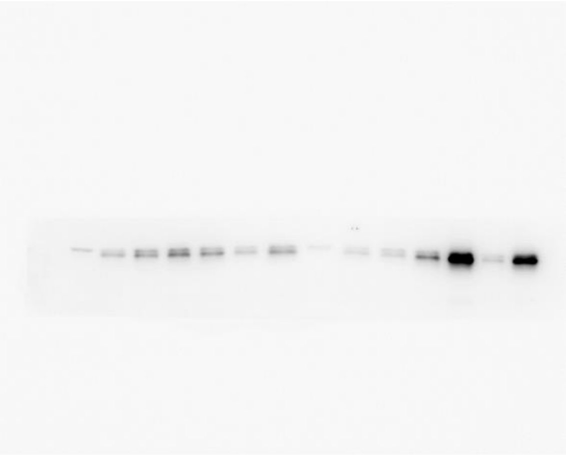

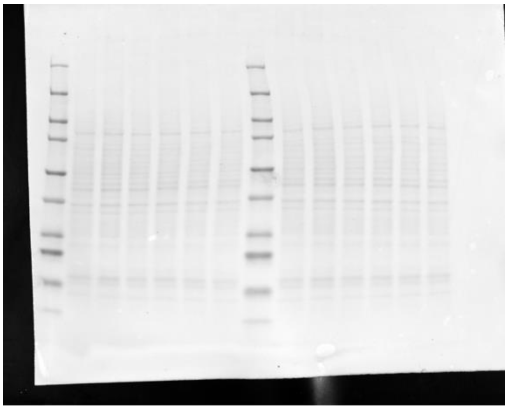

Ponceau S

ATF3

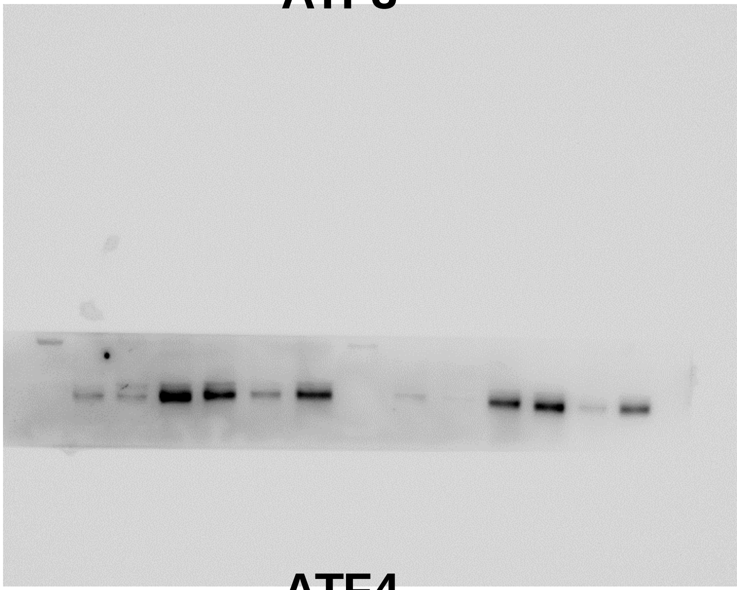

ATF4

Gel Loading Order (same for all blots):

1. Ladder
2. DMSO
3. +10 nM O/A
4. 10 μM FB231
5. 10 μM FB231 + 10 nM O/A
6. 5 μM MTK458
7. 5 μM MTK458 + 10 nM O/A

Figure S6A-B: Mass Spec Compound off-targets – repl 3 (representative)

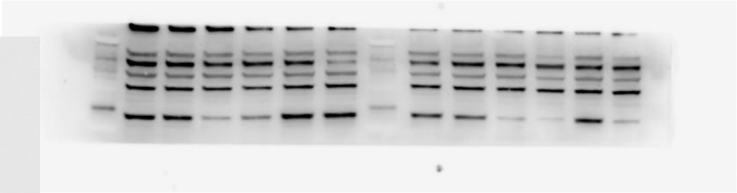

FECH

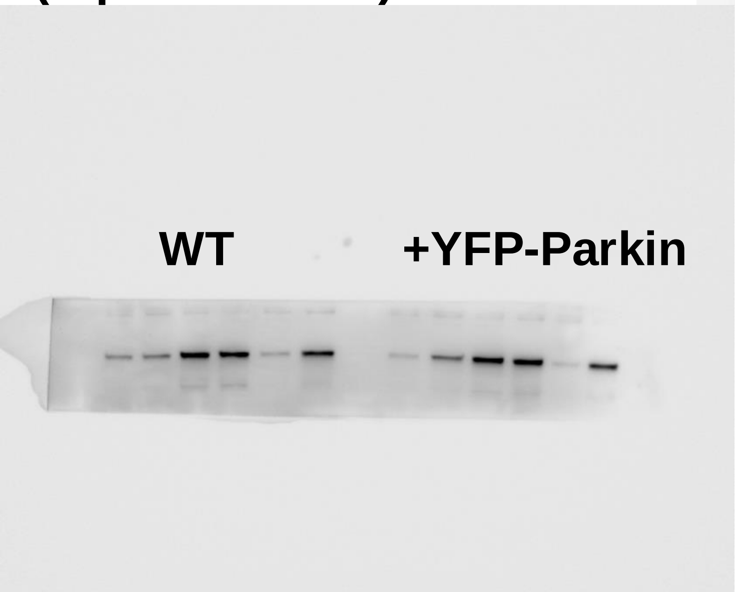

IFRD1

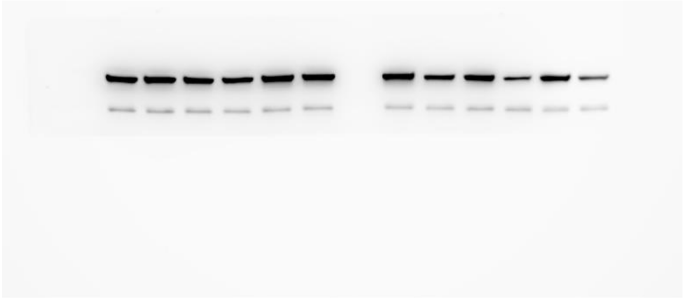

ATP5A

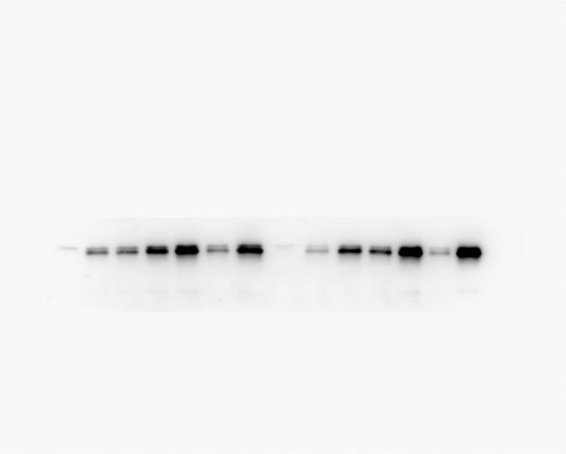

ATF3

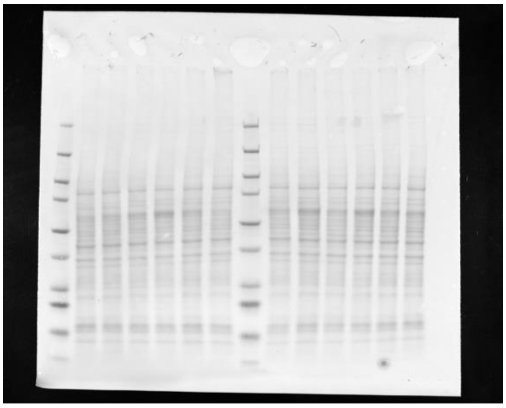

Ponceau S

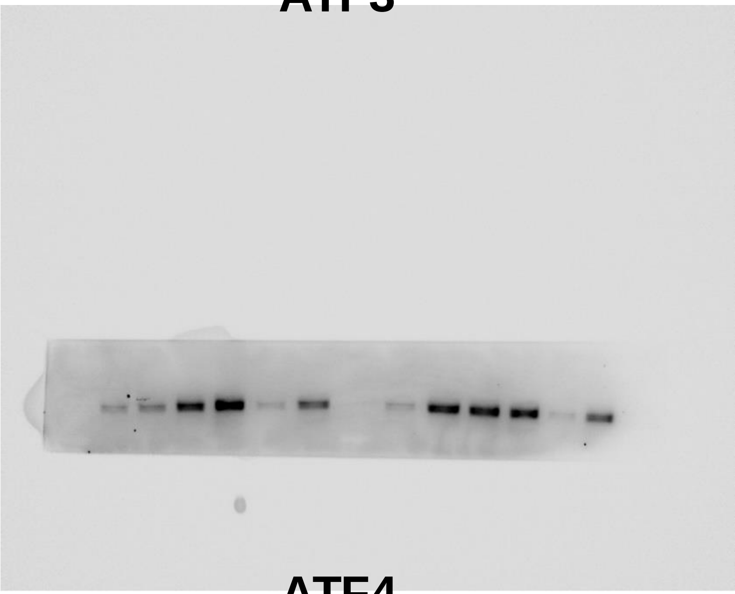

ATF4

Gel Loading Order (same for all blots):

1. Ladder
2. DMSO
3. +10 nM O/A
4. 10 µM FB231
5. 10 µM FB231 + 10 nM O/A
6. 5 µM MTK458
7. 5 µM MTK458 + 10 nM O/A

Figure S6E: Activator titration off-targets: +YFP-Parkin HeLa S3 – Repl. 1 (representative)

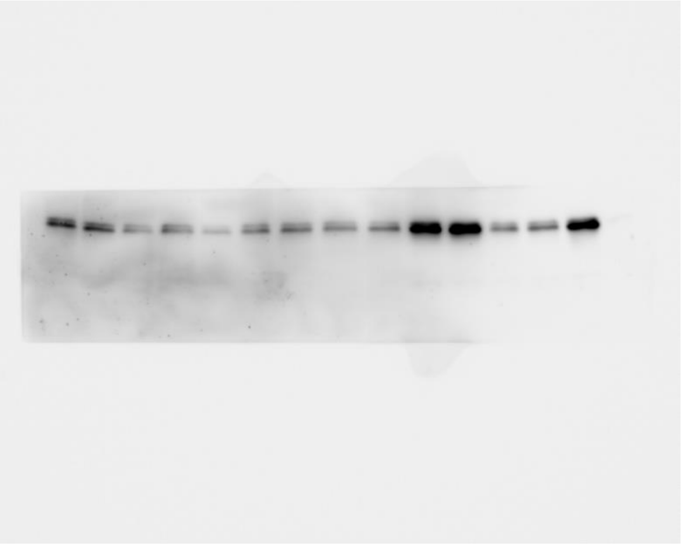

ATF3

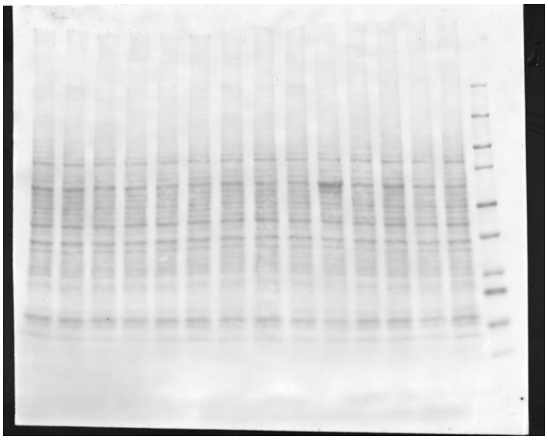

Ponceau S

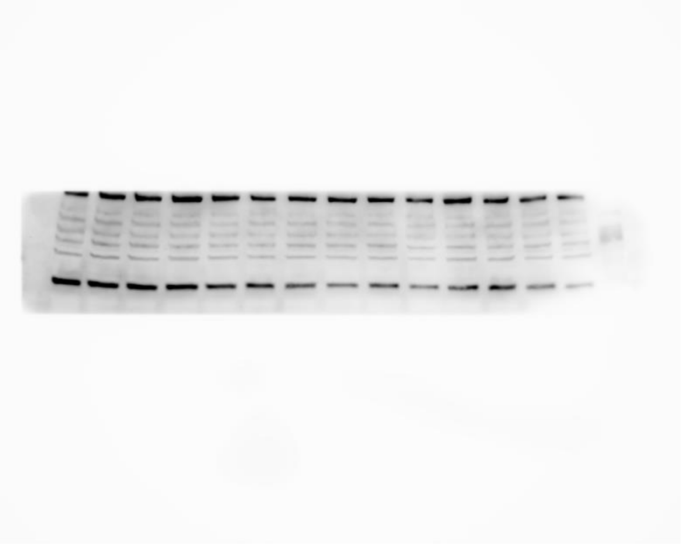

FECH

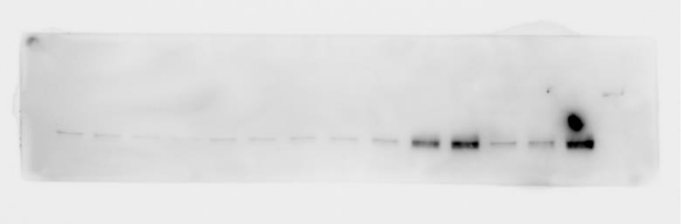

ATF4

Gel Loading Order (same for all blots):

1. DMSO
2. +10 nM O/A
3. 0.6 μM MTK458
4. 2.5 μM MTK458
5. 10 μM MTK458
6. 0.16 μM FB231
7. 0.6 μM FB231
8. 2.5 μM FB231
9. 0.6 μM MTK458 + 10 nM O/A
10. 2.5 μM MTK458 + 10 nM O/A
11. 10 μM MTK458 + 10 nM O/A
12. 0.16 μM FB231 + 10 nM O/A
13. 0.6 μM FB231 + 10 nM O/A
14. 2.5 μM FB231 + 10 nM O/A
15. Ladder

Figure S6E: Activator titration off-targets: +YFP-Parkin
HeLa S3 – Repl. 2

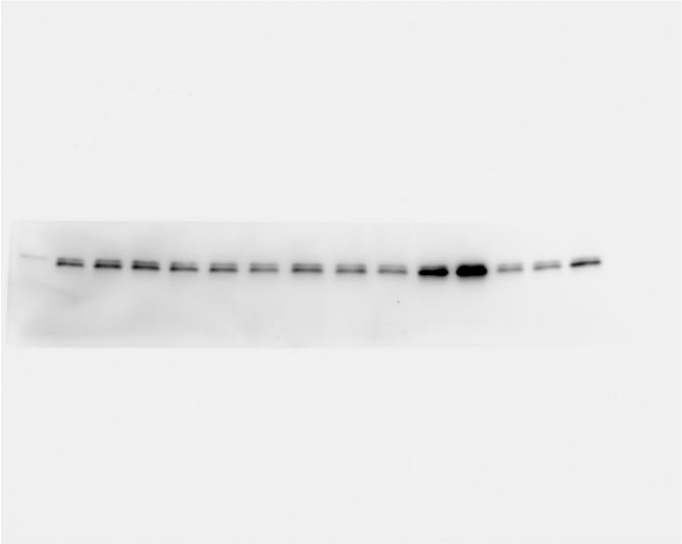

ATF3

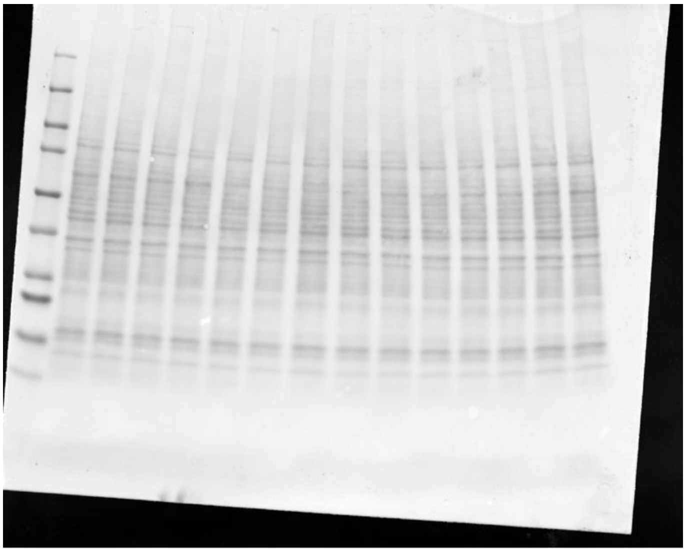

Ponceau S

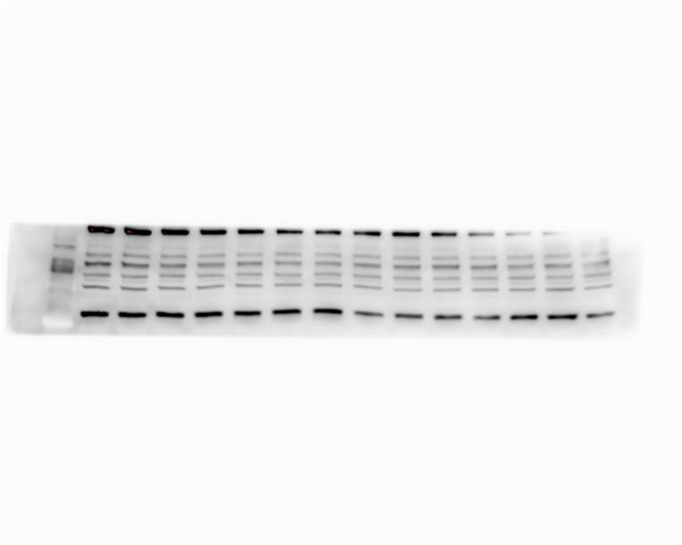

FECH

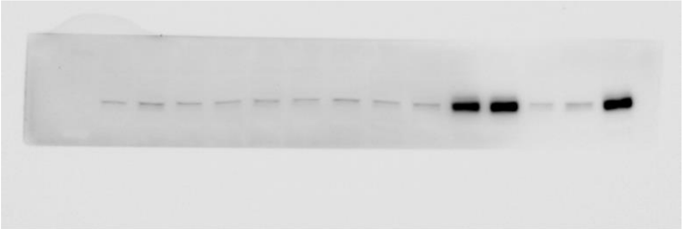

ATF4

Gel Loading Order (same for all blots):

1. Ladder
2. DMSO
3. +10 nM O/A
4. 0.6 μM MTK458
5. 2.5 μM MTK458
6. 10 μM MTK458
7. 0.16 μM FB231
8. 0.6 μM FB231
9. 2.5 μM FB231
10. 0.6 μM MTK458 + 10 nM O/A
11. 2.5 μM MTK458 + 10 nM O/A
12. 10 μM MTK458 + 10 nM O/A
13. 0.16 μM FB231 + 10 nM O/A
14. 0.6 μM FB231 + 10 nM O/A
15. 2.5 μM FB231 + 10 nM O/A

Fig S7A: Compound dose escalation mitophagy and stress – FB231 Repl. 1

+YFP-Parkin PINK1KO HeLa S3

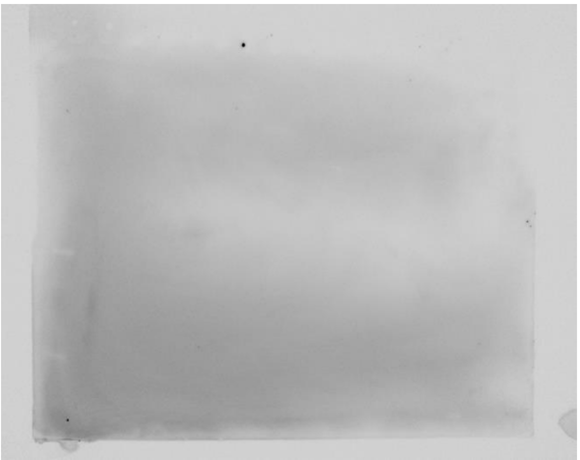

pUb

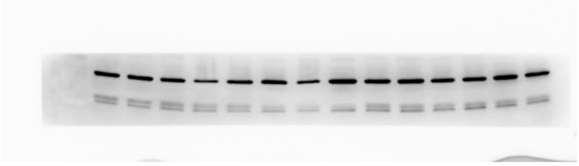

ATP5A

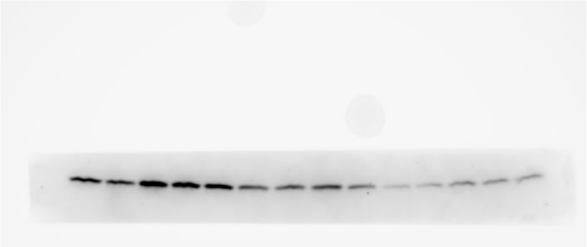

COX4I2

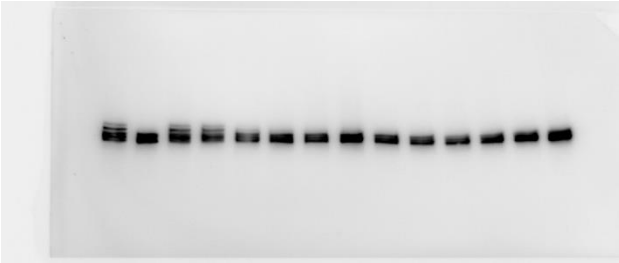

OPA1

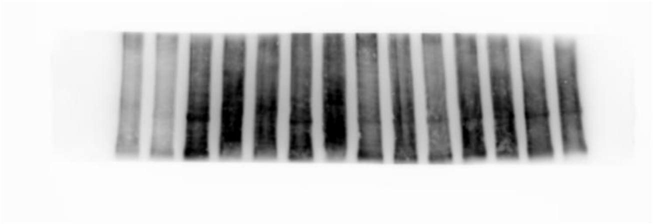

PARKIN

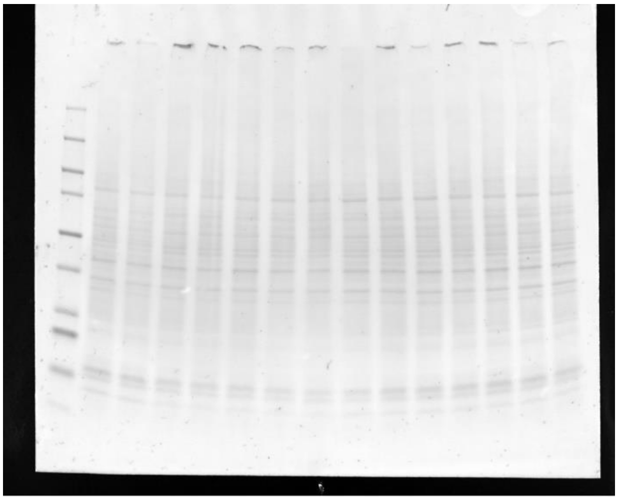

Ponceau S

Gel Loading Order (same for all blots):

1. Ladder
2. DMSO
3. +10 nM O/A
4. 0.3 μM FB231
5. 0.6 μM FB231
6. 1.25 μM FB231
7. 2.5 μM FB231
8. 5 μM FB231
9. 10 μM FB231
10. 0.3 μM FB231 + 10 nM O/A
11. 0.6 μM FB231 + 10 nM O/A
12. 1.25 μM FB231 + 10 nM O/A
13. 2.5 μM FB231 + 10 nM O/A
14. 5 μM FB231 + 10 nM O/A
15. 10 μM FB231 + 10 nM O/A

Fig S7A: Compound dose escalation (continued)–
FB231 PINK1KO repl 1

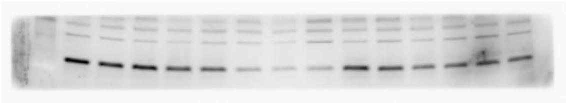

FECH

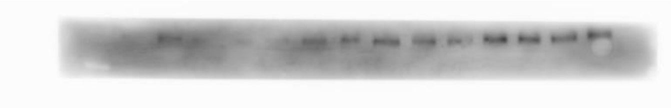

ATF4

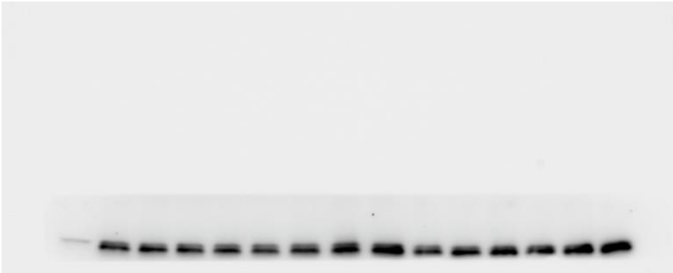

ATF3

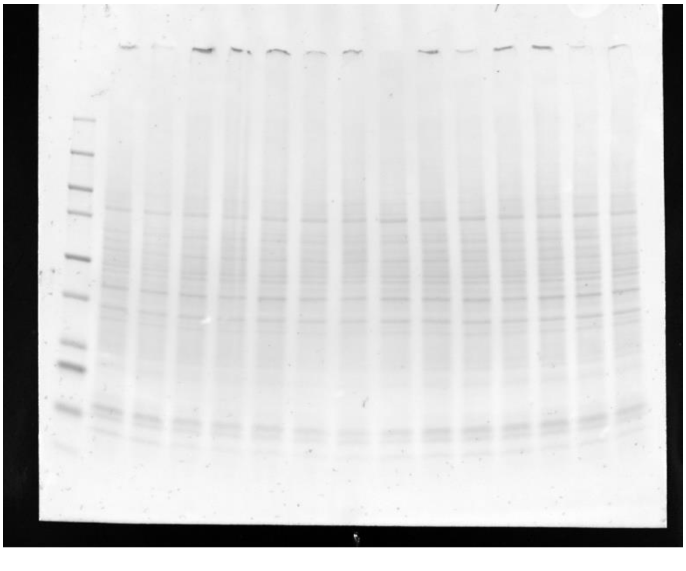

Ponceau S

Gel Loading Order (same for all blots):

1. Ladder
2. DMSO
3. +10 nM O/A
4. 0.3 μM FB231
5. 0.6 μM FB231
6. 1.25 μM FB231
7. 2.5 μM FB231
8. 5 μM FB231
9. 10 μM FB231
10. 0.3 μM FB231 + 10 nM O/A
11. 0.6 μM FB231 + 10 nM O/A
12. 1.25 μM FB231 + 10 nM O/A
13. 2.5 μM FB231 + 10 nM O/A
14. 5 μM FB231 + 10 nM O/A
15. 10 μM FB231 + 10 nM O/A

Figure S7A: Compound dose escalation mitophagy and stress – FB231 Repl. 2 (representative)

+YFP-Parkin/PINK1KO

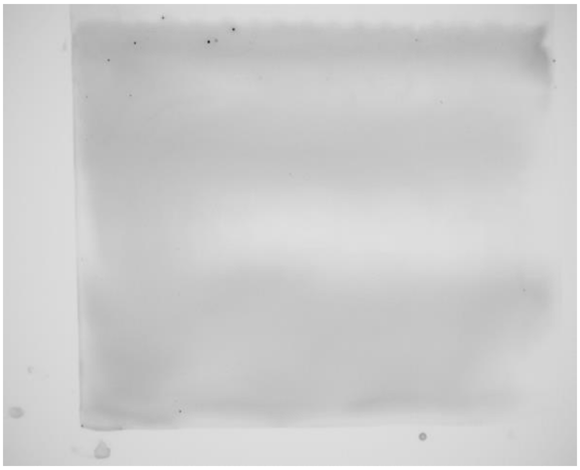

pUb

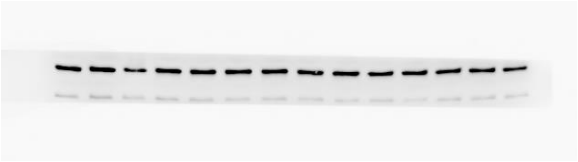

ATP5A

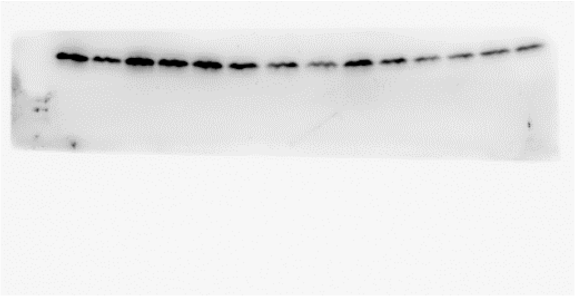

COX4I2

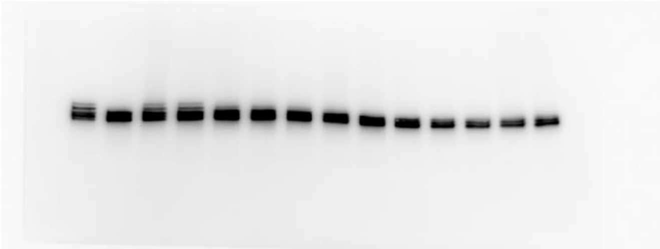

OPA1

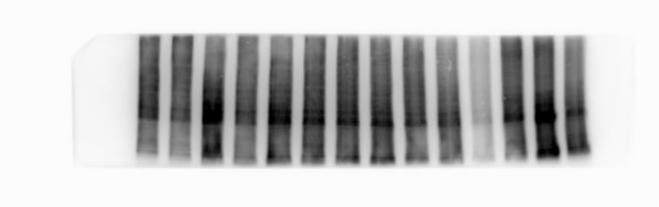

PARKIN

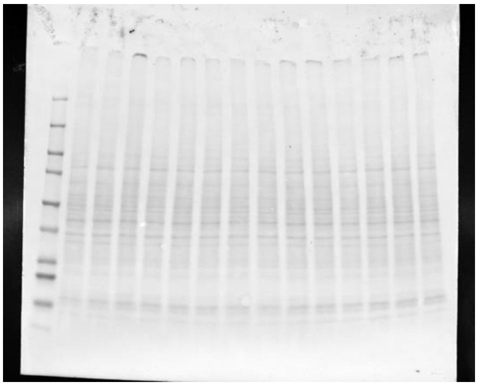

Ponceau S

Gel Loading Order (same for all blots):

1. Ladder
2. DMSO
3. +10 nM O/A
4. 0.3 μM FB231
5. 0.6 μM FB231
6. 1.25 μM FB231
7. 2.5 μM FB231
8. 5 μM FB231
9. 10 μM FB231
10. 0.3 μM FB231 + 10 nM O/A
11. 0.6 μM FB231 + 10 nM O/A
12. 1.25 μM FB231 + 10 nM O/A
13. 2.5 μM FB231 + 10 nM O/A
14. 5 μM FB231 + 10 nM O/A
15. 10 μM FB231 + 10 nM O/A

Fig S7A: Compound dose escalation (continued) – FB231 PINK1KO repl 2

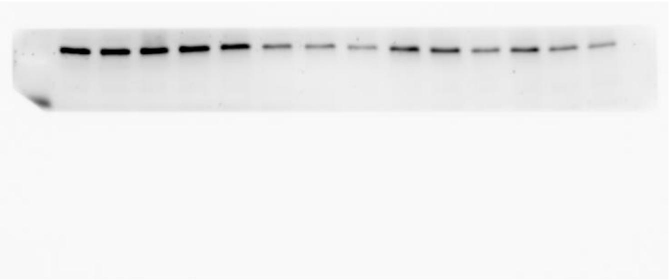

FECH

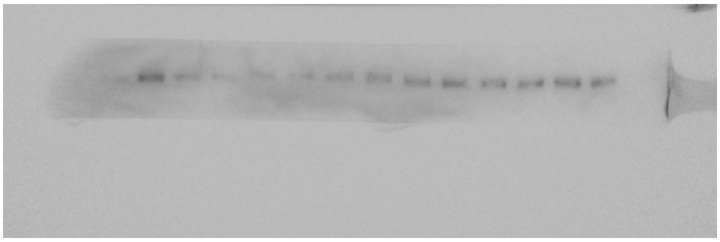

ATF4

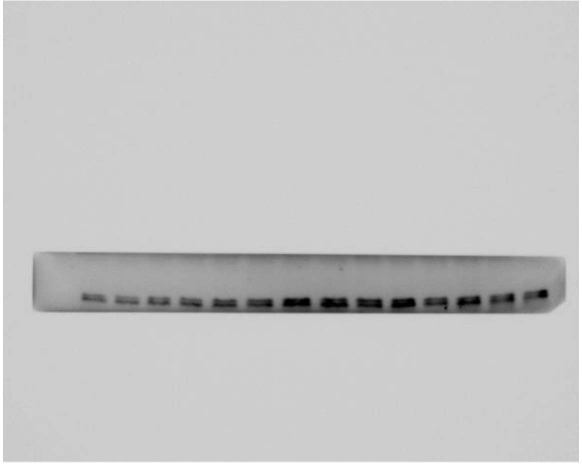

ATF3

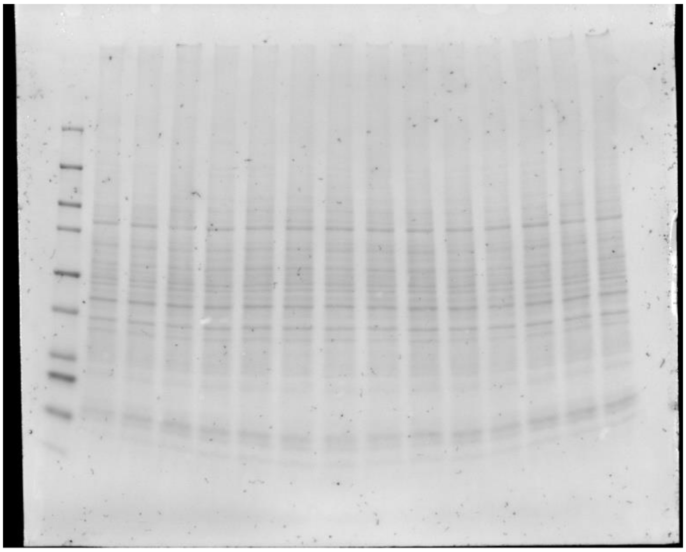

Ponceau S

Gel Loading Order (same for all blots):

1. Ladder
2. DMSO
3. +10 nM O/A
4. 0.3 μM FB231
5. 0.6 μM FB231
6. 1.25 μM FB231
7. 2.5 μM FB231
8. 5 μM FB231
9. 10 μM FB231
10. 0.3 μM FB231 + 10 nM O/A
11. 0.6 μM FB231 + 10 nM O/A
12. 1.25 μM FB231 + 10 nM O/A
13. 2.5 μM FB231 + 10 nM O/A
14. 5 μM FB231 + 10 nM O/A
15. 10 μM FB231 + 10 nM O/A

Fig S7C: ATF3 in WT, +YFP-Parkin, +YFP-Parkin/PINK1KO HeLa cells – repl 1 (representative)

ATF3

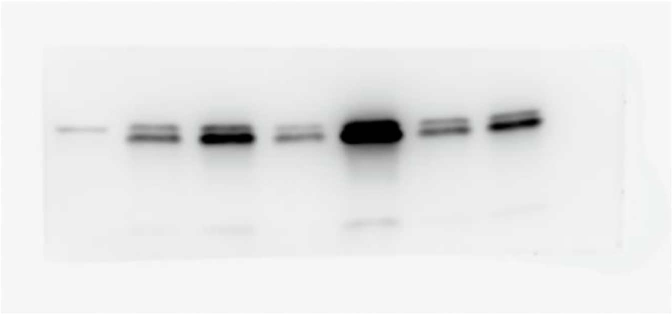

Gel Loading Order (same for all blots):

1. Ladder
2. WT HeLa DMSO
3. WT HeLa +100 nM O/A
4. YFP-Parkin HeLa DMSO
5. YFP-Parkin HeLa +100 nM O/A
6. YFP-Parkin HeLa/PINK1KO DMSO
7. YFP-Parkin HeLa/PINK1KO +100 nM O/A

Ponceau S

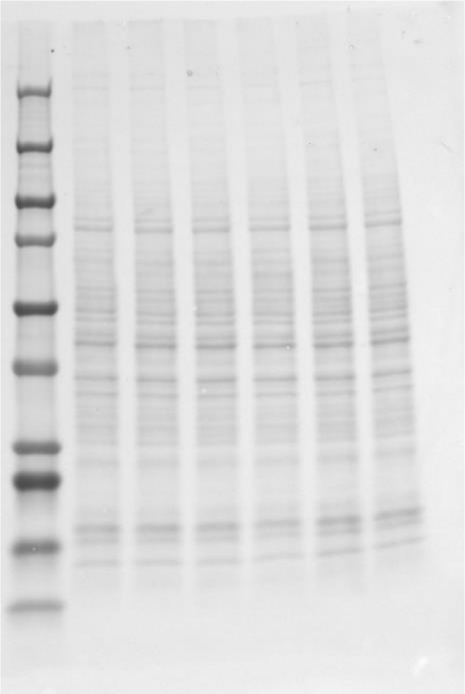

Fig S7C: ATF3 in WT, +YFP-Parkin, +YFP-Parkin/PINK1KO HeLa cells – repl 2

ATF3

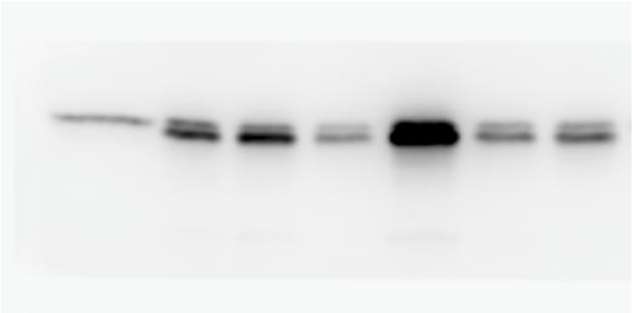

Gel Loading Order (same for all blots):

1. Ladder
2. WT HeLa DMSO
3. WT HeLa +100 nM O/A
4. YFP-Parkin HeLa DMSO
5. YFP-Parkin HeLa +100 nM O/A
6. YFP-Parkin HeLa/PINK1KO DMSO
7. YFP-Parkin HeLa/PINK1KO +100 nM O/A

Ponceau S

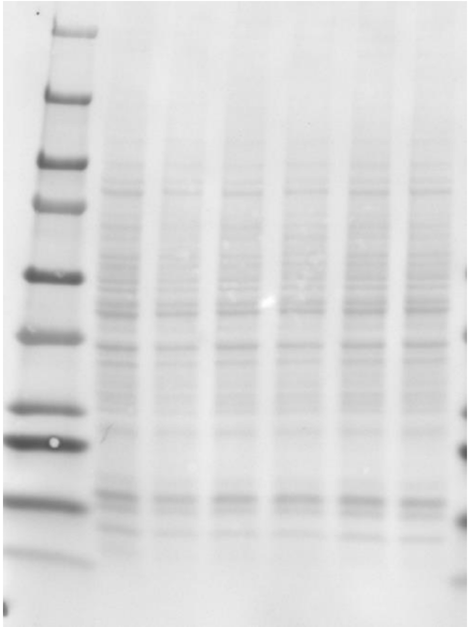

Fig S7C: ATF3 in WT, +YFP-Parkin, +YFP-Parkin/PINK1KO HeLa cells – repl 3

ATF3

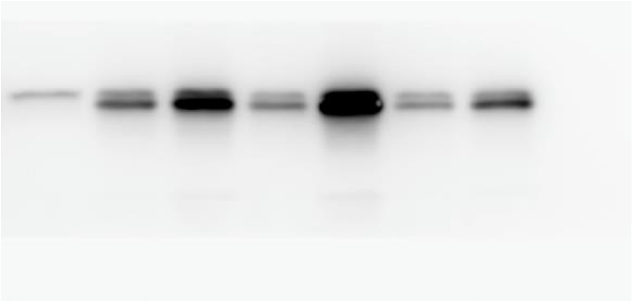

Gel Loading Order (same for all blots):

1. Ladder
2. WT HeLa DMSO
3. WT HeLa +100 nM O/A
4. YFP-Parkin HeLa DMSO
5. YFP-Parkin HeLa +100 nM O/A
6. YFP-Parkin HeLa/PINK1KO DMSO
7. YFP-Parkin HeLa/PINK1KO +100 nM O/A

Ponceau S

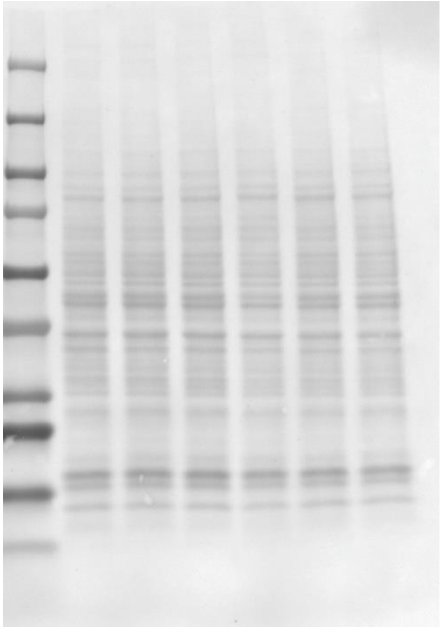

Figure S7E: DELE1-HA in endogenous DELE1-HA knock-in HEK293T cells

Repl 1 (representative)

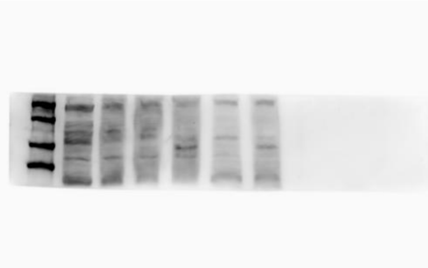

DELE1-HA

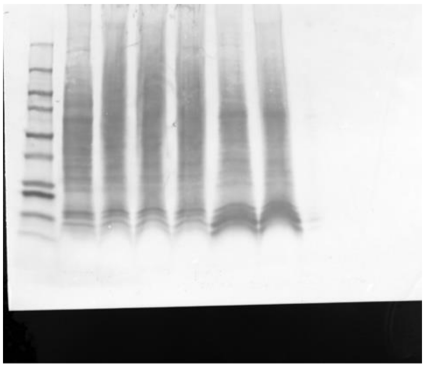

Ponceau S

Repl 2

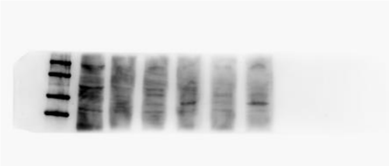

DELE1-HA

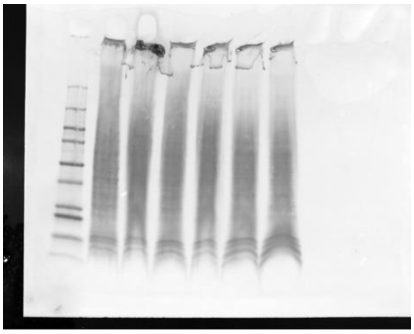

Ponceau S

Repl 3

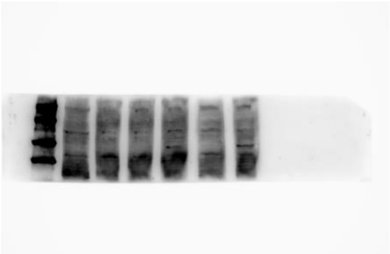

DELE1-HA

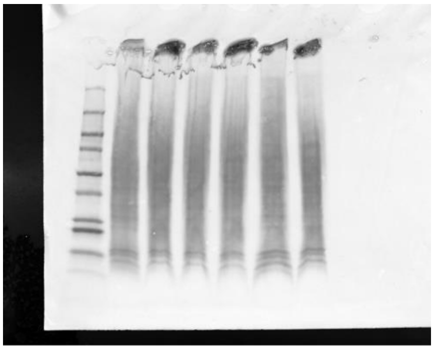

Ponceau S

Gel Loading Order (same for all blots):

1. Ladder
2. DMSO
3. +10 nM O/A
4. 10 µM FB231
5. 10 µM FB231 + 10 nM O/A
6. 10 µM MTK458
7. 10 µM MTK458 + 10 nM O/A
8. Blank
9. Blank
10. Blank

Ab Full Blots

YFP-Parkin/mt-Keima HeLa

Left Lane: DMSO
Right Lane: 100nM OA

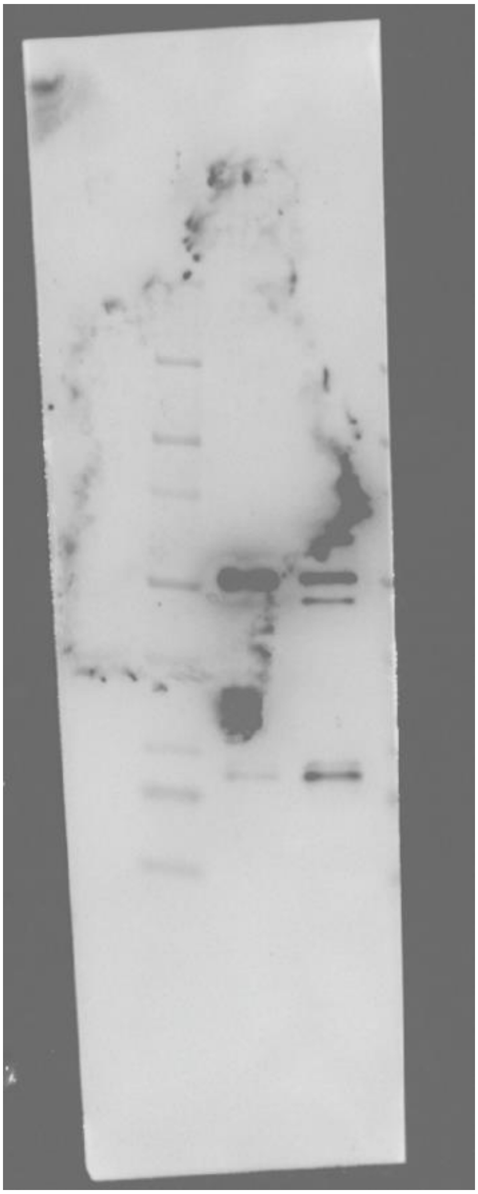

ATF3

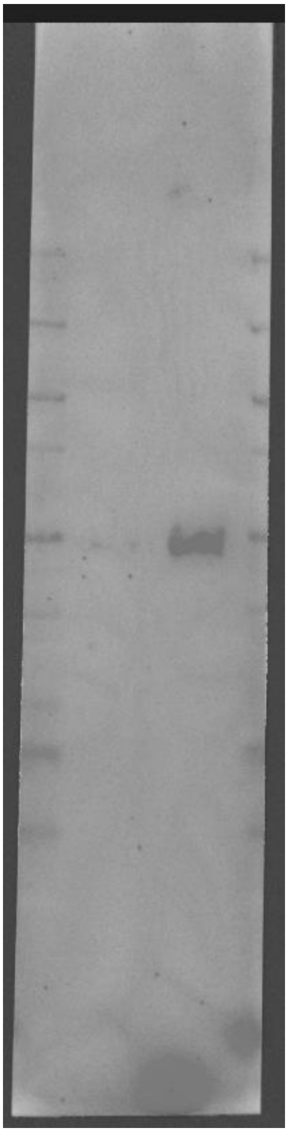

ATF4

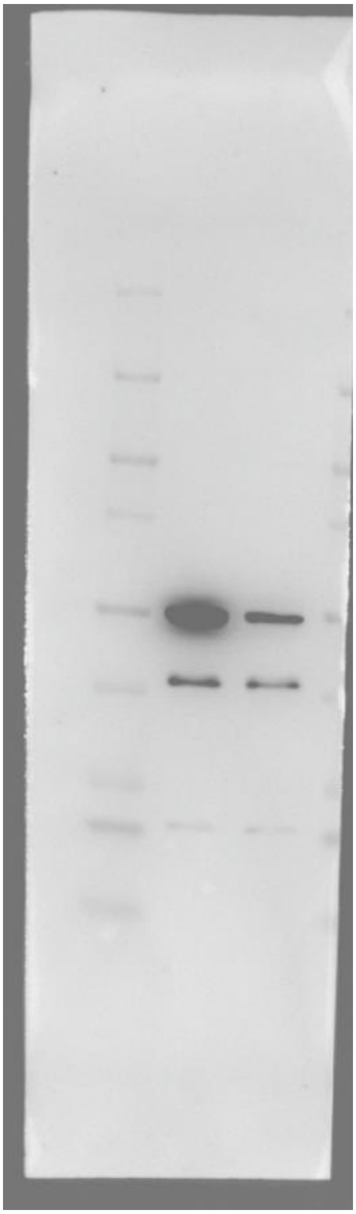

ATP5a

YFP-Parkin/mt-Keima HeLa

Left Lane: DMSO
Right Lane: 100nM OA

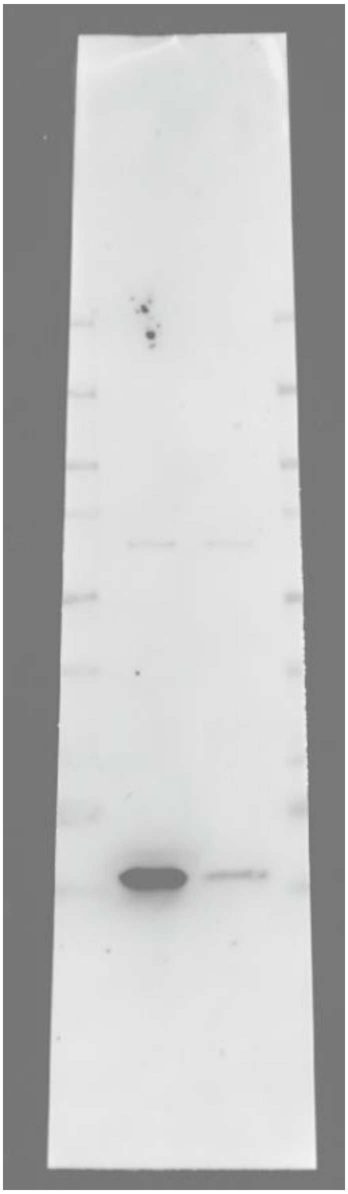

COX4I2

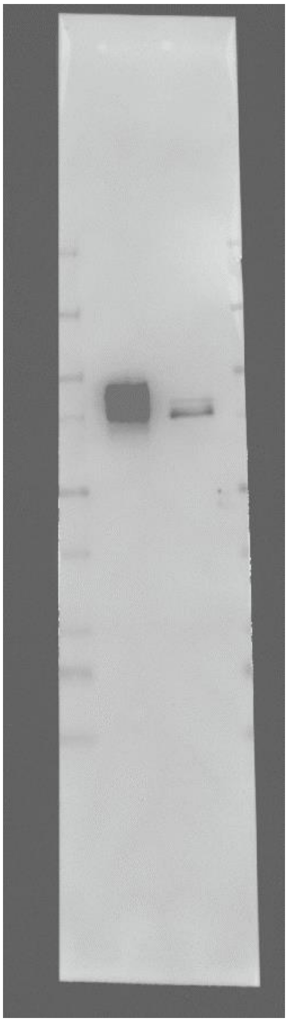

OPA1

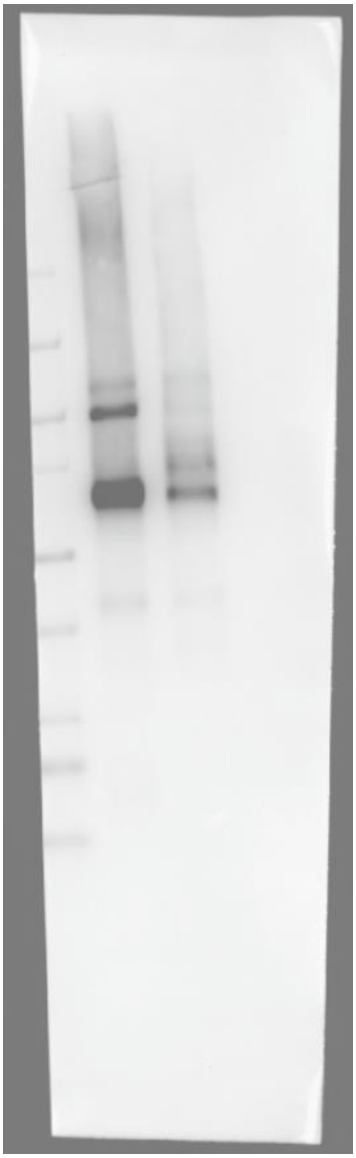

PARKIN

YFP-Parkin/mt-Keima HeLa

Left Lane: DMSO
Right Lane: 100nM OA

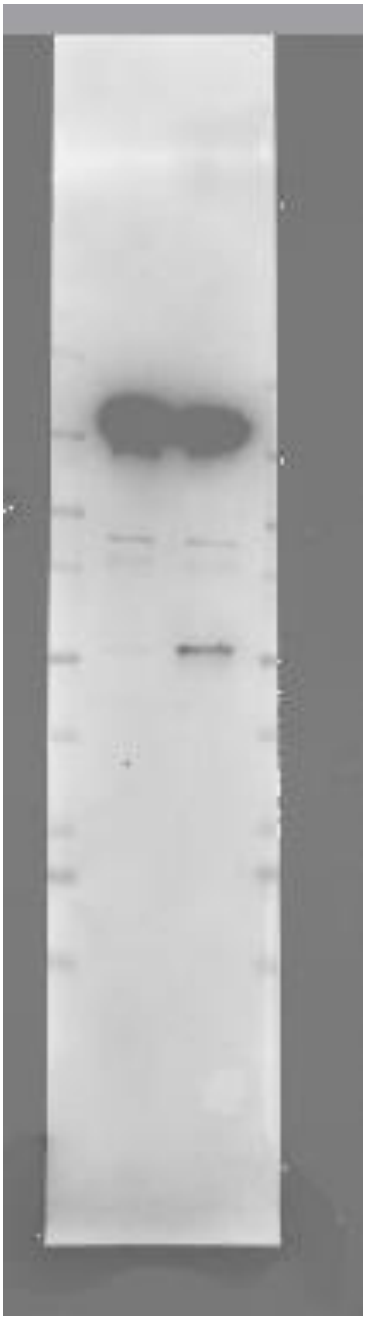

IFRD1

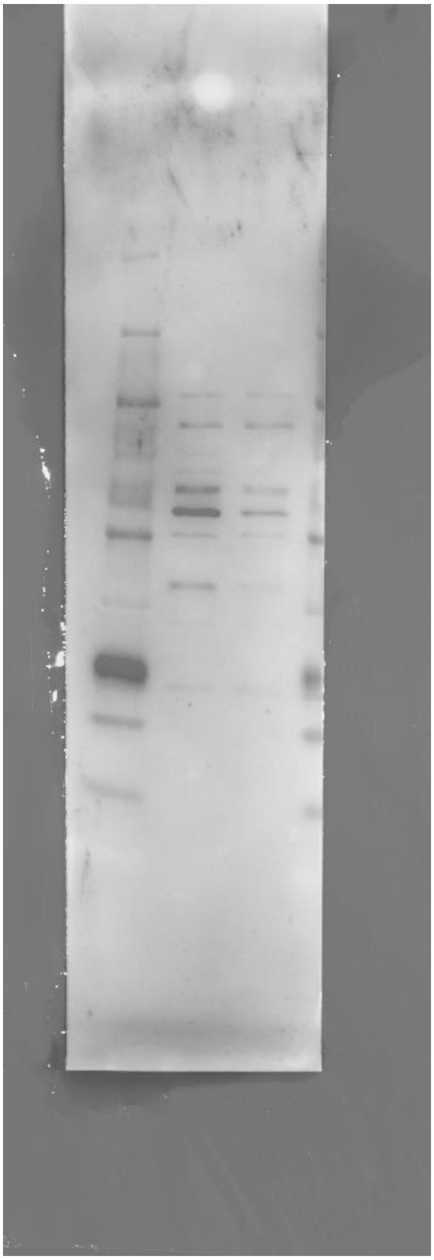

FECH

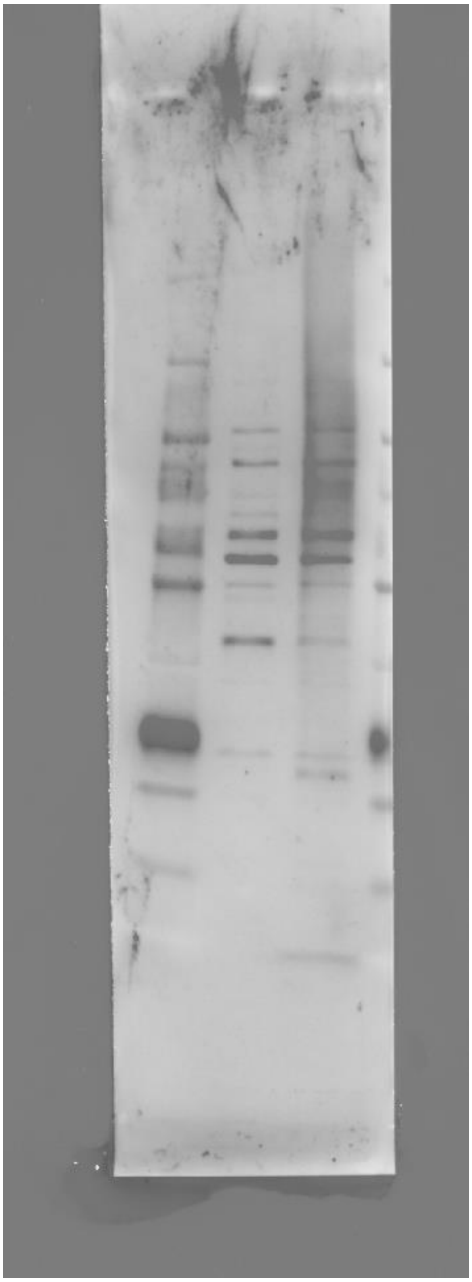

pUb

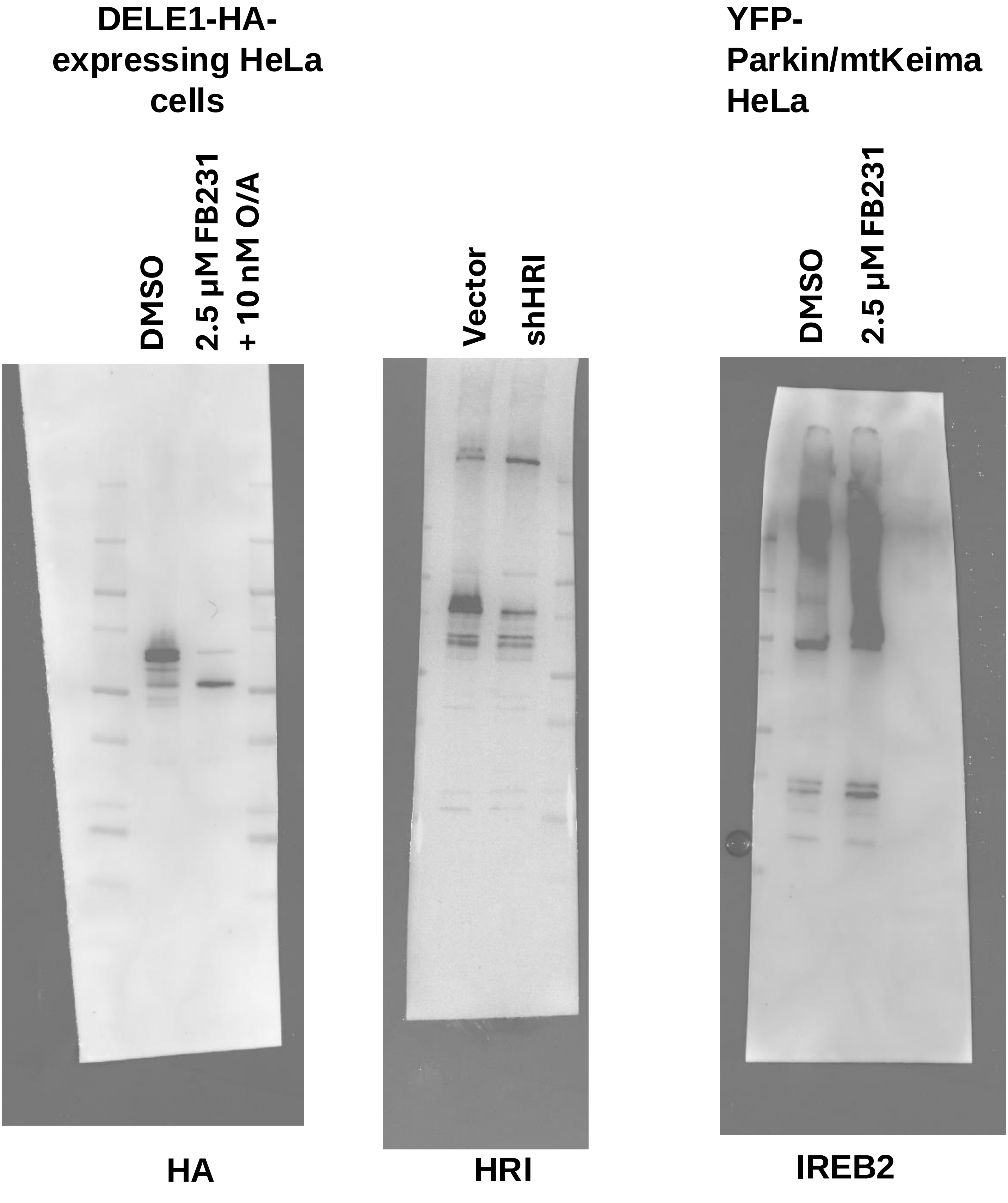
DELE1-HA-expressing HeLa cells
DMSO
2.5 μM FB231 + 10 nM O/A
Vector
shHRI
YFP-Parkin/mtKeima HeLa
DMSO
2.5 μM FB231
HA
HRI
IREB2

Other Supplementary Materials for this manuscript include the following:

Table S5: Global proteomics data for cells treated with FB231 or MTK458, related to Figure 4

Table S6: Source data for all experiments presented in this manuscript.

Code S1: Code for running an interactive simulation of strong and weak mitochondrial toxins on mitochondrial function and mitophagy
